# Y Fuse? Sex Chromosome Fusions in Fishes and Reptiles

**DOI:** 10.1371/journal.pgen.1005237

**Published:** 2015-05-20

**Authors:** Matthew W. Pennell, Mark Kirkpatrick, Sarah P. Otto, Jana C. Vamosi, Catherine L. Peichel, Nicole Valenzuela, Jun Kitano

**Affiliations:** 1 Institute for Bioinformatics and Evolutionary Studies, University of Idaho, Moscow, Idaho, United States of America; 2 Department of Integrative Biology, University of Texas, Austin, Austin, Texas, United States of America; 3 Department of Zoology, University of British Columbia, Vancouver, British Columbia, Canada; 4 Department of Biological Sciences, University of Calgary, Calgary, Alberta, Canada; 5 Divisions of Basic Sciences and Human Biology, Fred Hutchinson Cancer Research Center, Seattle, Washington, United States of America; 6 Department of Ecology, Evolution and Organismal Biology, Iowa State University, Ames, Iowa, United States of America; 7 Ecological Genetics Laboratory, National Institute of Genetics, Mishima, Shizuoka, Japan; University of Wisconsin—Madison, UNITED STATES

## Abstract

Chromosomal fusion plays a recurring role in the evolution of adaptations and reproductive isolation among species, yet little is known of the evolutionary drivers of chromosomal fusions. Because sex chromosomes (X and Y in male heterogametic systems, Z and W in female heterogametic systems) differ in their selective, mutational, and demographic environments, those differences provide a unique opportunity to dissect the evolutionary forces that drive chromosomal fusions. We estimate the rate at which fusions between sex chromosomes and autosomes become established across the phylogenies of both fishes and squamate reptiles. Both the incidence among extant species and the establishment rate of Y-autosome fusions is much higher than for X-autosome, Z-autosome, or W-autosome fusions. Using population genetic models, we show that this pattern cannot be reconciled with many standard explanations for the spread of fusions. In particular, direct selection acting on fusions or sexually antagonistic selection cannot, on their own, account for the predominance of Y-autosome fusions. The most plausible explanation for the observed data seems to be (a) that fusions are slightly deleterious, and (b) that the mutation rate is male-biased or the reproductive sex ratio is female-biased. We identify other combinations of evolutionary forces that might in principle account for the data although they appear less likely. Our results shed light on the processes that drive structural changes throughout the genome.

## Author Summary

Chromosome number is a basic feature of the eukaryotic genome that has important consequences for recombination, segregation, and other processes. Despite a century of research on the evolution of karyotype, however, we still have little understanding of the evolutionary forces that enable chromosomal fusions and fissions to become established. Here, we compare the rates of chromosomal fusions between sex chromosomes (X, Y, Z, and W chromosomes) and autosomes. We find that these fusions more frequently involve the Y chromosome than other sex chromosomes in fishes and squamate reptiles. To account for these observations, we conduct theoretical analyses and find that the most likely explanation for this pattern is that fusions have deleterious effects, and further that mutation rates and/or sex ratios are biased. Improving our knowledge of the evolutionary mechanisms driving sex chromosome-autosome fusions provides a richer understanding of the forces that shape chromosomes generally.

## Introduction

The number of chromosomes is one of the most fundamental features of a eukaryotic genome. Chromosome number varies, both between closely related species and within species, and such variation can contribute to divergent adaptation and speciation [1–5]. Shifts in chromosome number typically result from a reciprocal translocation between two acrocentric chromosomes, bringing together two linkage groups (“fusions” as reinterpreted by [6]) or by splitting a metacentric chromosome into two (“fissions”). Although genetic drift, selection for changes in recombination rate, and meiotic drive are thought to play a role [7,8], the evolutionary forces that allow fusions and fissions to fix within a population remain obscure.

Sex chromosome evolution offers a unique glimpse into these forces. The X and Y chromosomes of male-heterogametic species (as in mammals) and the Z and W chromosomes of female-heterogametic species (as in birds) differ in many aspects of their evolutionary environments. While Y and W chromosomes are often thought to be evolutionarily similar, Y chromosomes spend all of their evolutionary history in males, while W chromosomes spend none. X and Z chromosomes also differ: X chromosomes spend 1/3 of their evolutionary history in males, while Z chromosomes spend 2/3 of their history in males. Consequently, the four types of sex chromosomes vary in how selection acts on them, in their effective population sizes, in their mutation rates, and in how meiotic drive acts on them [9–12]. All of these factors might play a role in the evolution of chromosomal rearrangements, and so differences in rates of rearrangement among sex chromosomes offer clues to what evolutionary conditions favor changes in genome structure.

Structurally, sex chromosomes are the most rapidly evolving parts of the genome in many groups of animals [2,11,13–15]. In some taxa, such as fishes and squamate reptiles, both XY and ZW sex determination is found among closely related species (and even among populations within a species) [14,16]. Further, fusions between sex chromosomes and autosomes are relatively easy to detect from karyotypic data, and a large number of such fusions have been discovered [2,17]. Thus there are many phylogenetically independent events, providing the opportunity to test whether fusions involving the four different types of sex chromosomes are equally likely to occur and/or establish within a species.

**Fig 1:**
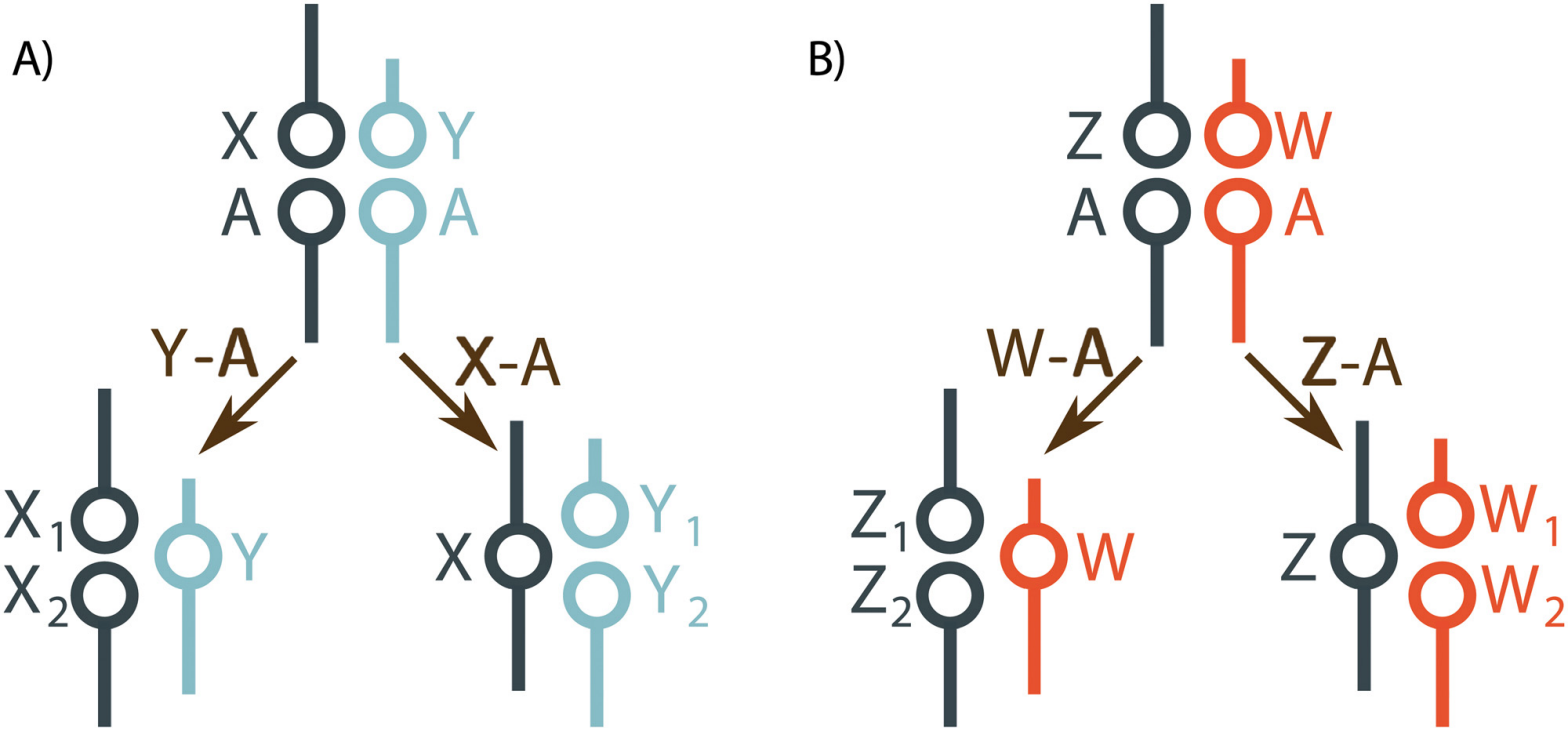
Sex chromosome-autosome fusions create multiple sex chromosome systems. (A) In XY systems, X-autosome (X-A) and Y-autosome fusions (Y-A) make XY_1_Y_2_ and X_1_X_2_Y systems, respectively. (B) In ZW systems, Z-autosome (Z-A) and W-autosome fusions (W-A) make ZW_1_W_2_ and Z_1_Z_2_W systems, respectively.

A fusion between a sex chromosome and an autosome is usually detected because it creates an odd number of chromosomes in one sex (Fig 1) [2,18]. With XY sex determination, a Y-autosome fusion creates an X_1_X_2_Y system, with the unfused homologue segregating as a neo-X chromosome. Likewise, X-autosome fusions generate XY_1_Y_2_ systems, Z-autosome fusions generate ZW_1_W_2_ systems, and W-autosome fusions generate Z_1_Z_2_W systems. These neo-sex chromosome systems can often be identified by light microscopy, without molecular cloning or linkage mapping. This has enabled cytogenetic studies to identify many species with sex chromosome-autosome fusions [2,19–22]. These data have yet to be used to estimate rates of different types of sex chromosome-autosome fusions.

Three main evolutionary forces have been thought to be important to the establishment of fusions. The first is direct selection. While chromosome rearrangements are often considered deleterious [1,23], chromosomal translocations may alter the expression of genes near the breakpoint [18,24], which may sometimes be beneficial [3,5]. A second mechanism that has been proposed to establish fusions is sexually antagonistic selection at an autosomal locus [25]. A fusion with a sex chromosome can cause an allele that is beneficial in one sex to spend most or all of its evolutionary life in that sex. Meiotic drive is a third force. During female meiosis in animals, one of the meiotic products goes into the egg, while the others are discarded in the polar bodies. In some species, female meiotic drive preferentially transmits fused chromosomes to eggs, while unfused chromosomes go into polar bodies [26,27]. This situation favors X-autosome fusions because they experience female meiosis in two out of every three generations. In other species, female meiotic drive preferentially transmits unfused chromosomes, which selects against X-autosome fusions [21]. Limited data suggests that male meiosis in mammals can also favor the transmission of fused chromosomes [28,29]. While these evolutionary forces are known to affect the spread of sex chromosome-autosome fusions, it is unknown how they shape the relative establishment rates of fusions with different sex chromosomes.

We begin this study by analyzing a large new data set that includes information on the sex determination system and karyotypes across the tree of life [17]. We focus on fishes and squamate reptiles because these taxa include many independent origins of XY and ZW systems [19,20], allowing us to assess differences in the rates of fusions. We find that Y-autosome fusions become established at a much higher rate than any of the other three types of sex chromosome-autosome fusions. This then motivates us to develop an integrated body of analytic models that predict the relative establishment rates for the different types of fusions. The models incorporate a large number of potentially important factors: deleterious and beneficial fitness effects, sexually antagonistic selection, female meiotic drive, genetic drift, sex-biased mutation rates, and biased sex ratios. We find that the data cannot be explained by models of selection unless there is also some mechanism generating a difference between the sexes, including sex-biased mutation rates, biased sex ratios, or sex-specific selection (including meiotic drive). A particularly plausible explanation is that fusions are slightly deleterious, fix by drift, and occur more frequently in males.

## Results

### Sex chromosome-autosome fusions often involve the Y in fishes and squamates

We begin by analyzing the patterns of chromosome fusions in vertebrates, based on our recent compilation of sex chromosome data [17]. Hereafter, we refer to the fusion between a Y chromosome and an autosome as Y-A fusion, and similarly for other sex chromosomes. Examining the raw counts (Table 1), two interesting patterns emerge.

**Table 1:** Observed number of species with multiple sex chromosome systems in vertebrates.

Taxa	Y-A fusion (X_1_X_2_Y)	X-A fusion (XY_1_Y_2_)	W-A fusion (Z_1_Z_2_W)	Z-A fusion (ZW_1_W_2_)	XY systems^§^	ZW systems^§^
Fish*	42	3	0^★^	2^★^	109	38
Amphibians	1	0	0	0	29	16
Reptiles	40	0	2	4	120	240
Birds	-	-	0	3	0	192
Mammals	18^#^	24^#^	-	-	467	0

Only X_1_X_2_Y, XY_1_Y_2_, Z_1_Z_2_W, and ZW_1_W_2_ systems are counted here.

*Erythrinus erythrinus was counted as a Y-A fusion (B-D sub-populations), although unfused chromosomes also exist in this species [50].

^★^In addition, *Ancistrus* sp.2 exhibits both W-A and Z-A fusions (Z_1_Z_2_W_1_W_2_)

^#^ In addition, *Ornithorhynchus anatinus* (X_1_X_2_X_3_X_4_X_5_Y_1_Y_2_Y_3_Y_4_Y_5_) and *Tachyglossus aculeatus* (X_1_X_2_X_3_X_4_X_5_Y_1_Y_2_Y_3_Y_4_) exhibit both Y-A and X-A fusions.

^§^XO systems (*n* = 12 in fishes, *n* = 3 in mammals), ZO systems (*n* = 3 in fishes), and WO systems (*n* = 1 in amphibians) are not included, nor are cases with multiple segregating sex determining mechanisms (*n* = 8 in mammals).

First, there are more species with Y-A fusions (101 species) than with X-A fusions (27 species). The excess of Y-A fusions over X-A fusions is particularly strong in fishes and squamate reptiles, while the numbers are closer to equality in mammals (Table 1). Second, sex chromosomes in XY lineages are more often fused than those in ZW lineages (Table 1). In fishes, 41% (45/109) of XY species have fused sex chromosomes, whereas only 5% (2/38) of ZW species do (Fisher’s exact test *P* < 0.001). In reptiles, 33% (40/120) of XY species have fusions, whereas only 3% (6/240) of ZW species do (Fisher’s exact test *P* < 0.001). Such counts, however, do not take into consideration the phylogenetic relationships among species.

**Fig 2:**
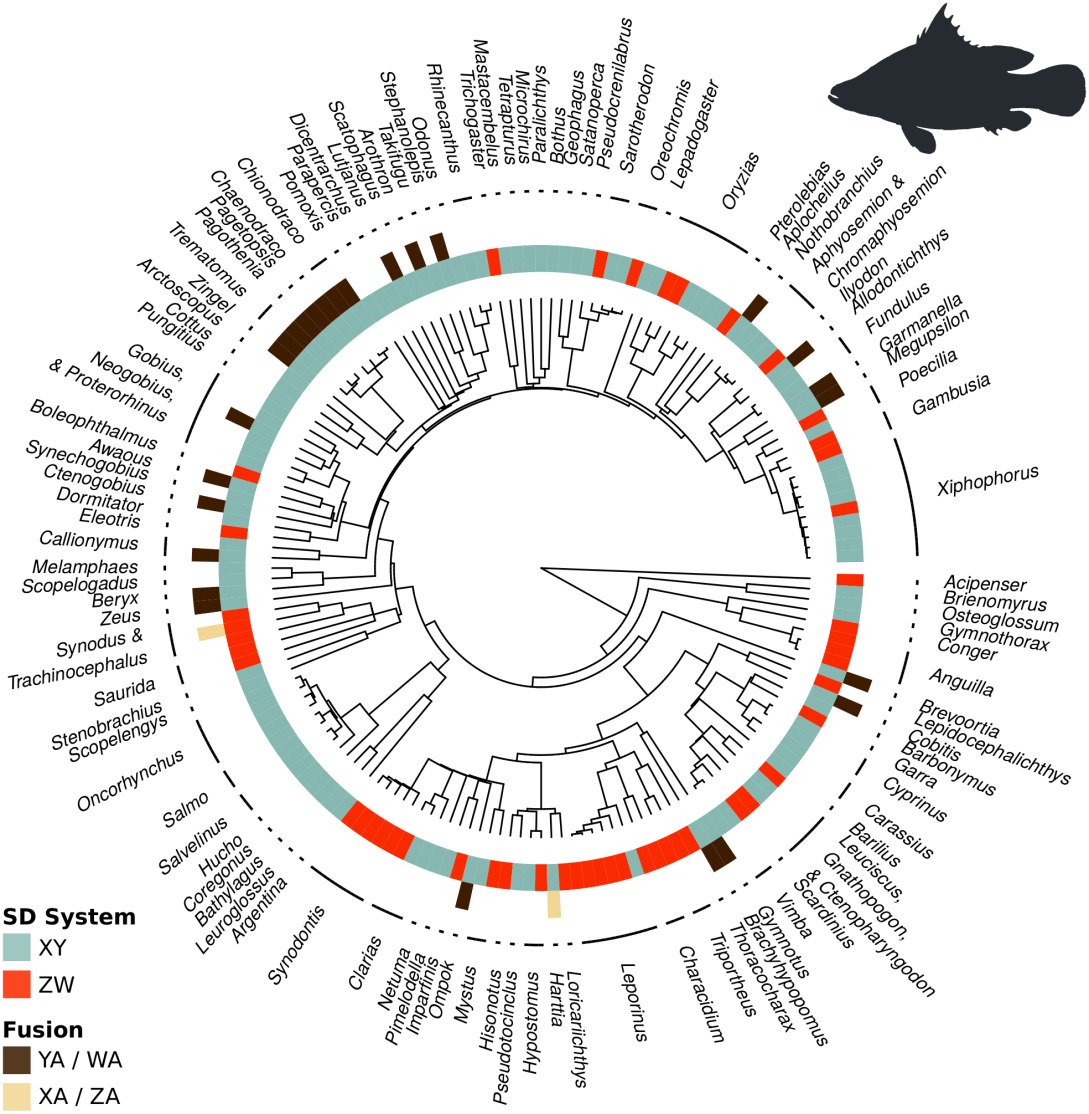
Sex chromosome fusions (outer circle) and sex determination system (inner circle) mapped onto the phylogenetic tree of fishes. The vast majority of fusions occur in XY systems (aqua) and involve Y-A fusions (brown).

**Fig 3:**
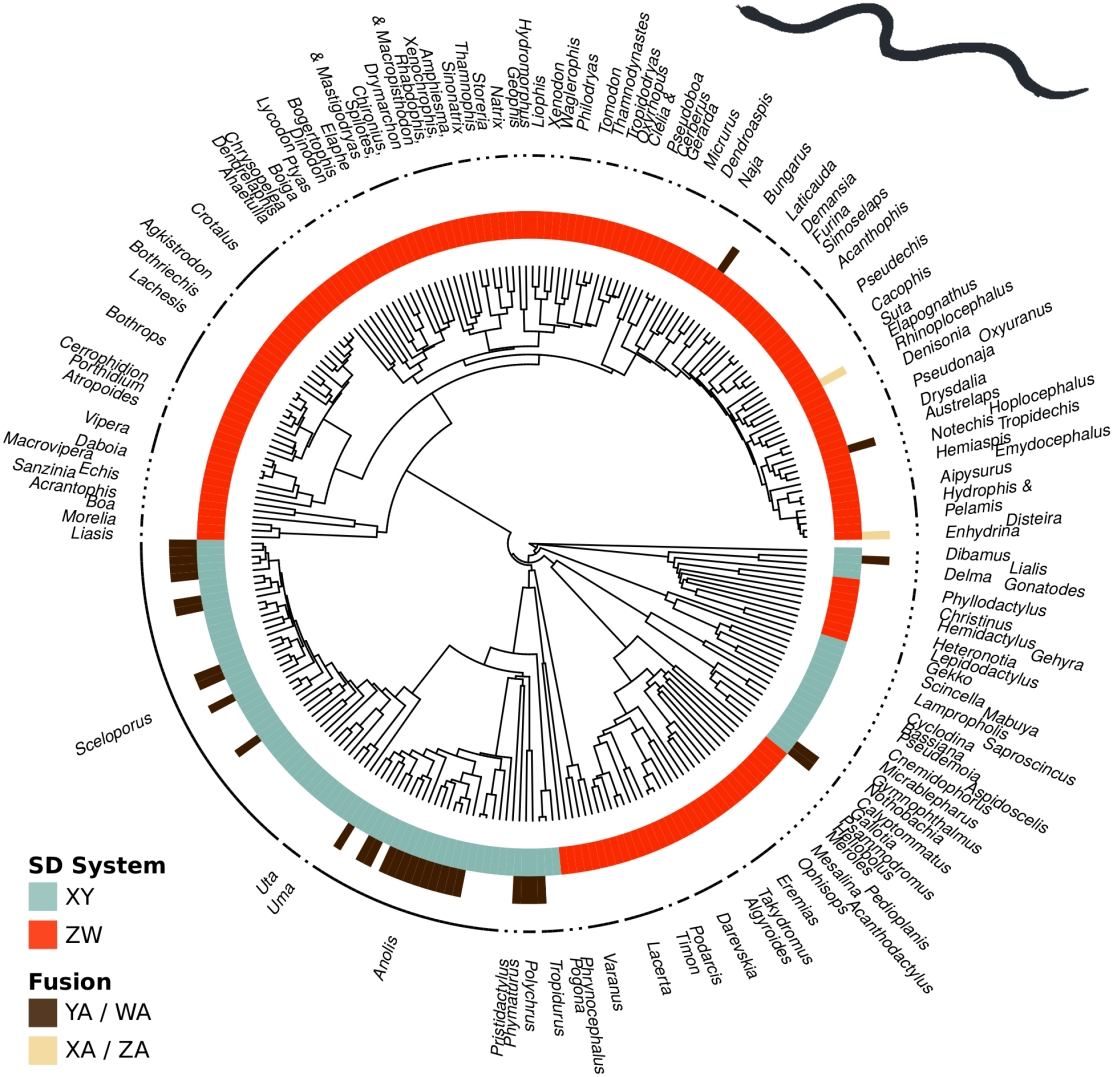
Sex chromosome fusions (outer circle) and sex determination system (inner circle) mapped onto the phylogenetic tree of squamate reptiles. The vast majority of fusions occur in XY systems (aqua) and involve Y-A fusions (brown).

To assess the relative rates of the establishment of fusions, we mapped fusion status onto the phylogenetic trees of fishes (Fig 2) and squamate reptiles (Fig 3). This resulted in datasets containing 163 species of fishes and 261 species of squamate reptiles. We then estimated transition rates between the chromosomal states using Markov chain Monte Carlo (MCMC) methods (see Methods for details).

**Fig 4:**
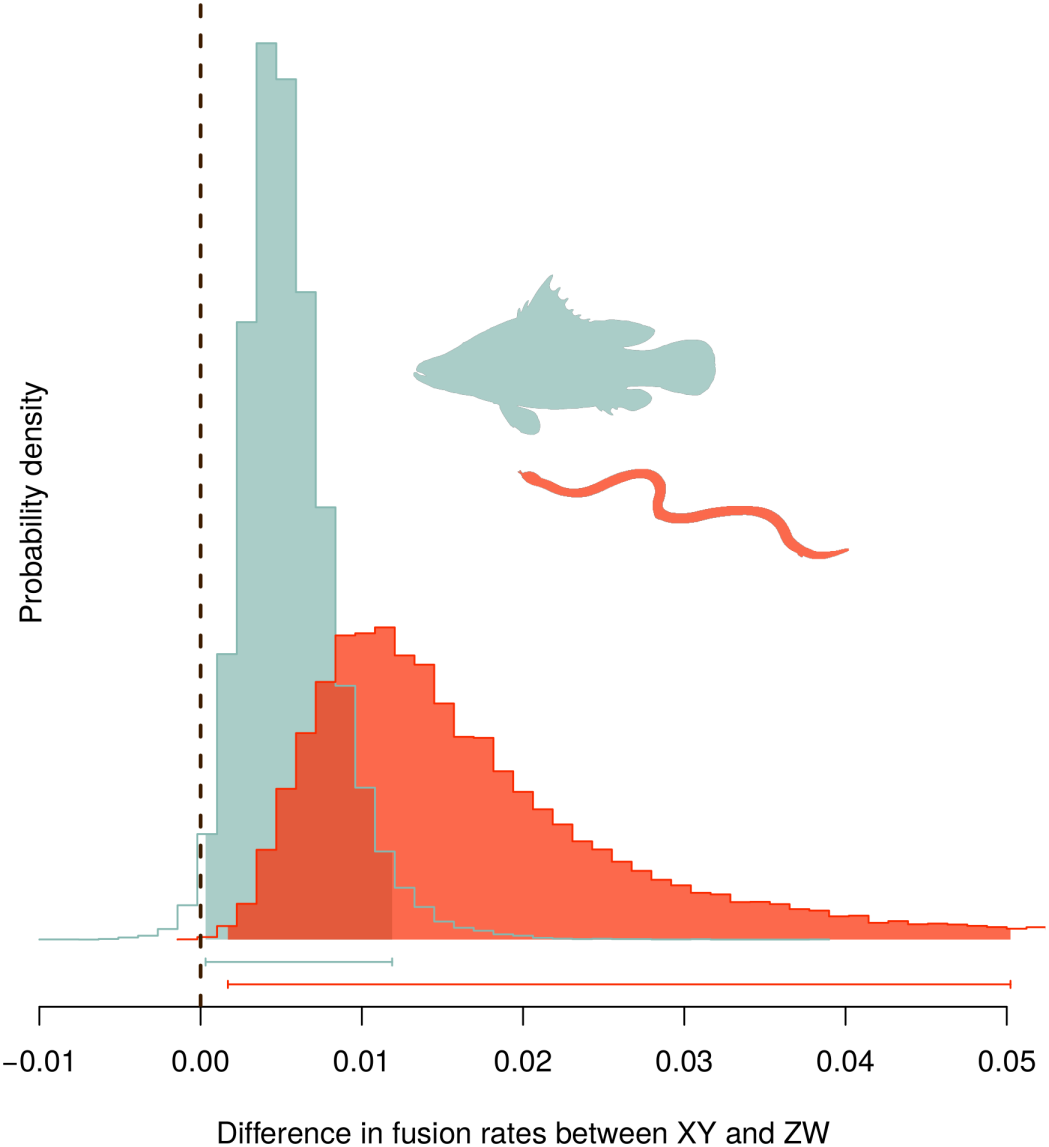
Posterior probability density of the difference in fixation rates of fusions between autosomes and sex chromosomes (rates in XY species minus in ZW species). The plot illustrates the difference in fusion rates over the last 40,000 steps of an MCMC chain, with the 95% credibility intervals shown by the horizontal bars below the figure.

We first examined whether XY and ZW systems differ in the rate of fusions. In fish, 98.6% of the posterior probability density suggests that fusions occur at a higher rate in XY than in ZW lineages (Fig 4). In squamates, 99.9% of the posterior probability density supports this conclusion (Fig 4). These analyses are based on a reduced model where fissions were allowed to occur at an equal rate in XY and ZW systems, although similar results are obtained if we allow both fusion and fission rates to differ between sex determining systems (S1 and S2 Figs).

We next asked if fusion rates differ for the four types of sex chromosomes (see S1 Text). We found that Y-A fusions establish at a higher rate than other sex chromosomes, even when accounting for the shared evolutionary history among taxa (S3 Fig for fish and S4 Fig for squamates).

### Theoretical analysis

To evaluate the plausibility of various mechanisms to explain the excess of fusions involving Y chromosomes, we compared the rate of establishment of different sex chromosome-autosome fusions under various evolutionary scenarios. The core results are derived in S1 Text, where we present expressions for the rates at which fusions with the four types of sex chromosomes are established. These results follow the standard population genetic practice (e.g., [9]) of modeling establishment rates as the product of the rate of appearance and fixation probability for mutations of interest (here fusions), explicitly allowing for sex-biased mutation rates and biased sex ratios.

To facilitate comparison to the data, we focus on the establishment rates for Y-A, Z-A, and W-A fusions relative to the rate of X-A fusions. We begin by studying the neutral case, where selection is absent. We allow, however, for sex-biased mutation rates and biased sex ratios among breeding individuals. We then ask how these neutral results are altered by the three main evolutionary forces thought to impact the rate of fusions: direct selection, meiotic drive, and sexually antagonistic selection.

#### Neutral case

We first consider the case without any selection or drive in the model. The overall establishment rates for fusions are given by the mutation rates generating each type of fusion (S1 Text, equation (A6)). Interestingly, the sex ratio does not enter into these results. Among newborns, each copy of a particular sex chromosome has an equal chance of being the progenitor of the entire population of that sex chromosome at some distant point in the future, regardless of subsequent changes in the survival and reproductive success of males versus females, which is a standard result in population genetics.

Sex-biased mutation alters the relative frequencies at which different types of neutral fusions arise and become fixed. Empirically, the sexes differ in the rate at which fusions arise: data from humans indicates that reciprocal translocations are predominantly paternal in origin [6,30–34]. If mutation is male-biased but does not depend on the type of chromosome (that is, the X and Y chromosomes in a male are equally likely to fuse), then Y-A fusions will become fixed most frequently (see eq. A7 in S1 Text). In this case, however, Z-A fusions would be almost as common as Y-A fusions (at least 2/3 as common, see eq. A7), which is not seen in the data (Figs 2 and 3). Thus the hypothesis that sex chromosome-autosome fusions are selectively neutral does not appear consistent with the observed data.

#### Direct fitness effects

**Fig 5:**
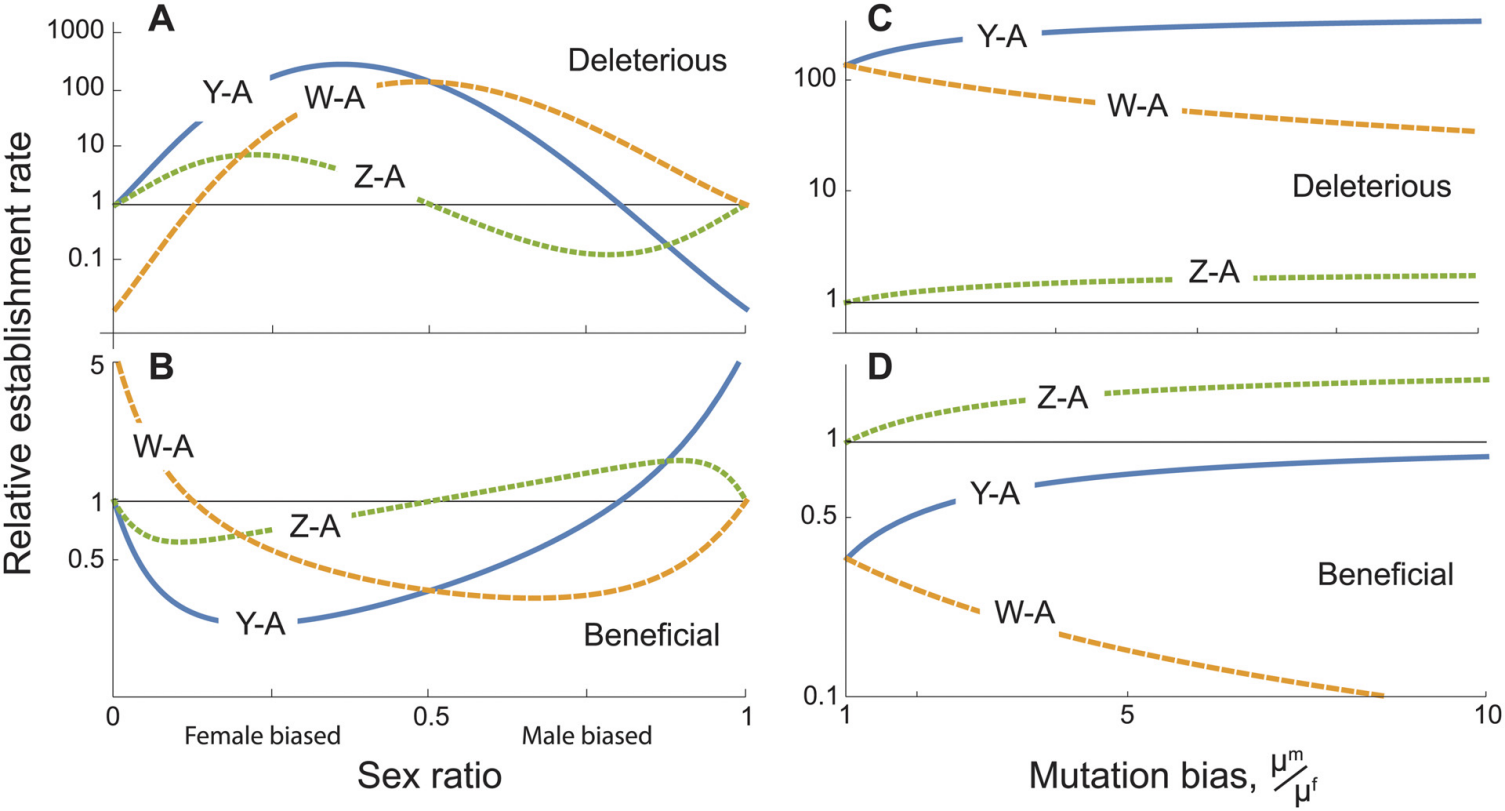
Establishment rates of sex chromosome-autosome fusions under direct selection, relative to the rate for X-A fusions. (*A*), (*B*) Effect of sex ratio bias among reproductive adults, *N* ^*m*^/(*N* ^*f*^ + *N* ^*m*^), assuming *μ* ^*m*^ = *μ* ^*f*^. (*C*), (*D*) Effect of the relative mutation rate for fusions in males versus females, *μ* ^*m*^/*μ* ^*f*^, assuming *N* ^*f*^ = *N* ^*m*^. Mutations are deleterious (*s* = -0.0003) in panels (*A*), (*C*) and beneficial (s = 0.0003) in panels (*B*), (*D*). Parameters: *N* ^*f*^ + *N* ^*m*^ = 10000.

We next ask how relative establishment rates depend on the direct fitness effects of a fusion (S1 Text). We begin by assuming that the fusion has an additive effect on fitness and that all else is equal (unbiased sex ratios and mutation rates, and equal fitness effects for all types of fusions). The establishment rates of X-A fusions and Z-A fusions are then equal, as are the rates of Y-A and W-A fusions (equation A.5 and A.6 in S1 Text, Fig 5). In this case, the rate at which fusions involving a Y or W chromosome establish relative to fusions involving a X or Z is $\left(1+e^{-4sN^{sex}}+e^{-2sN^{sex}}\right)/3$, where *N* ^*sex*^ is the number of reproductive adults of each sex and *s* is the fitness effect of the fusion. Thus, deleterious fusions (*s* < 0) are much more likely to involve the Y or W chromosome, because of the smaller population size of these chromosomes (Fig 5A and 5C). Conversely, beneficial fusions are more likely to involve X or Z chromosomes because they are more numerous and so more often the targets of beneficial fusions (Fig 5B and 5D).

However, direct selection alone cannot produce the observed pattern in which Y-A fusions are more common than W-A fusions. Similarly, direct selection, on its own, cannot explain why fusions in XY lineages are more common than in ZW lineages. To account for the observed data, therefore, we must invoke a combination of direct selection and sex biases, either in the sex ratio or in the mutation rate.

We use the term *reproductive sex ratio* to refer to the fraction of males among the adult population that can successfully reproduce, excluding individuals that either fail to survive or breed (see S1 Text for further details). Sexual selection is often stronger in males, which decreases the number of potentially reproducing males and leads to a female-biased reproductive sex ratio [35]. This situation will make Y-A fusions more common than any other type if fusions are deleterious (Fig 5A). That is because deleterious fusions, which are established by drift, will fix most often in sex chromosomes that have the smallest effective population size. By contrast, if fusions are beneficial, then Y-A fusions are unlikely to be the most common type of fusion (Fig 5B), because of their small effective population size. An exception to that conclusion occurs when there is an extremely male-biased sex ratio, with many fewer breeding females than males, which is considered rare in natural populations.

A second asymmetry that may account for the data is sex-biased mutation. As in the neutral case, we find that Y-A fusions will be most common when they are deleterious if they arise more often in males than females (blue, Fig 5B).

These results strictly apply only when the fusion has an additive effect on fitness, but more general results for arbitrary dominance derived in the supplemental *Mathematica* package (S2 and S3 Texts) show that the relative frequencies of establishment for the different types of fusions are robust to changes in dominance. Among other results, S2 Text shows that underdominant selection on fusions cannot explain the preponderance of Y-A fusions, because Y-A fusions always remain heterozygous and would be expected to suffer the attendant fitness disadvantage generated by underdominance.

In sum, the observed data are consistent with the hypothesis that fusions are deleterious and further that sex ratios are female-biased or mutation rates are male-biased. Under this hypothesis, fusions join the list of mechanisms that contribute to degeneration of Y chromosomes [36].

#### Meiotic drive

We next consider meiotic drive, which is thought to favor fused autosomes in some species of mammals and unfused chromosomes in others [26,27]. If meiotic drive is weak, we can treat it as a form of direct selection, and so equations (A4) and (A5) in S1 Text continue to apply. For clarity, we focus here on meiotic drive in females. (The results apply to meiotic drive in males if we interchange the sexes and the sex chromosomes, e.g., drive in ZW females becoming equivalent to drive in XY males.) For females who are heterozygous for the fusion, we denote the relative probability that they transmit the fusion to an egg as (1 + *f*). If unfused chromosomes are preferentially transmitted to the egg, *f* is negative. Averaging over the sexes, the effect of weak meiotic drive on an X-A fusion is equivalent to direct selection with a coefficient *s*_*X*_ = 2*f* /3. (The factor of 2/3 appears because drive acts only when the fusion is in a female). Thus when female meiotic drive favors fused chromosomes, the probability that an X-A fusion fixes is higher than the probability for a Y-A fusion, which never experiences female meiotic drive (*s*_*Y*_ = 0). In ZW systems, a W-A fusion is always carried by females and so benefits in every generation when drive favors fused chromosomes (*s*_*W*_ = *f*), while Z-A fusions enjoy that advantage only one generation out of every three (*s*_*Z*_ = *f*/3). Finally, to find the relative rates that these fusions establish we take into account how the numbers of each chromosome type affects the rate that fusions enter the population (S1 Text).

Even with unbiased mutation rates and sex ratios, Y-A fusions are expected to establish at the highest rate, followed by W-A fusions, Z-A fusions, and finally X-A fusions if female meiotic drive favors unfused chromosomes. The relative rankings are reversed if female meiotic drive favors fused chromosomes. Thus the observed excess of Y-A fusions can be explained by meiotic drive in females if unfused chromosomes benefit from drive more often than fused chromosomes.

Meiotic drive in males rather than in females can also establish Y-A fusions more often than X-A fusions, as long as drive favors fusions. Under these conditions, however, Z-A fusions will establish even more often (because there are three times as many Z chromosomes as Y chromosomes, and the Z spends 2/3 of its time in males). Thus, male meiotic drive alone cannot account for the excess of Y-A fusions over any other type of fusion, all else being equal.

These effects of meiotic drive are robust to modest sex biases in mutation rates and the reproductive sex ratio. Large biases can, however, cause the relative order of establishment rates to switch in a manner that is qualitatively similar to that seen previously for fusions with direct fitness effects (see graphs in the S2 Text).

In sum, meiotic drive by itself does not seem a likely explanation for the observed excess of Y-A fusions. Only female meiotic drive that consistently favors unfused chromosomes could generate that pattern. Data from mammals, however, suggest that female meiotic drive favors fused chromosomes in some lineages, but unfused chromosomes in other lineages [26,27].

#### Sexually antagonistic selection

To study fusions driven by sexually antagonistic selection, we developed a model that allows for sex-differences in selection (S1 Text). We assume that an autosomal locus segregates for alleles whose frequencies are at equilibrium before the fusion appears. This equilibrium only occurs under some fitness values [37], and the following results apply only when those conditions are met.

The fixation probability of a newly arisen fusion depends on several factors: which chromosome fuses with the autosome, whether the fusion originates in a male or a female, and which of the two alleles is captured by the fusion. We assume that fusions capture one of these two alleles randomly, in proportion to its frequency. We also assume that, once fused, the sexually antagonistic locus and the sex-determining region are completely linked. When drift is weak relative to selection, fusions establish primarily when they happen to capture the allele favored in the sex in which the fused chromosome spends the most time, i.e., Y-A and Z-A fusions that capture a male-beneficial allele, and X-A and W-A fusions that capture a female-beneficial allele.

Interestingly, if all else is equal (specifically, no sex biases in mutation rates or the reproductive sex ratio), the establishment rate of fusions is equal for all types of sex chromosomes (equation A10). Sexually antagonistic selection tends to favour Y-A fusions and W-A fusions more strongly than X-A and Z-A fusions because these chromosomes are consistently found in a single sex [25]. This advantage, however, is exactly balanced by the lower rate that such fusions originate in the population because there are fewer Y and W chromosomes than X and Z chromosomes. Consequently, sexually antagonistic selection alone causes no difference in establishment rates.

**Fig 6:**
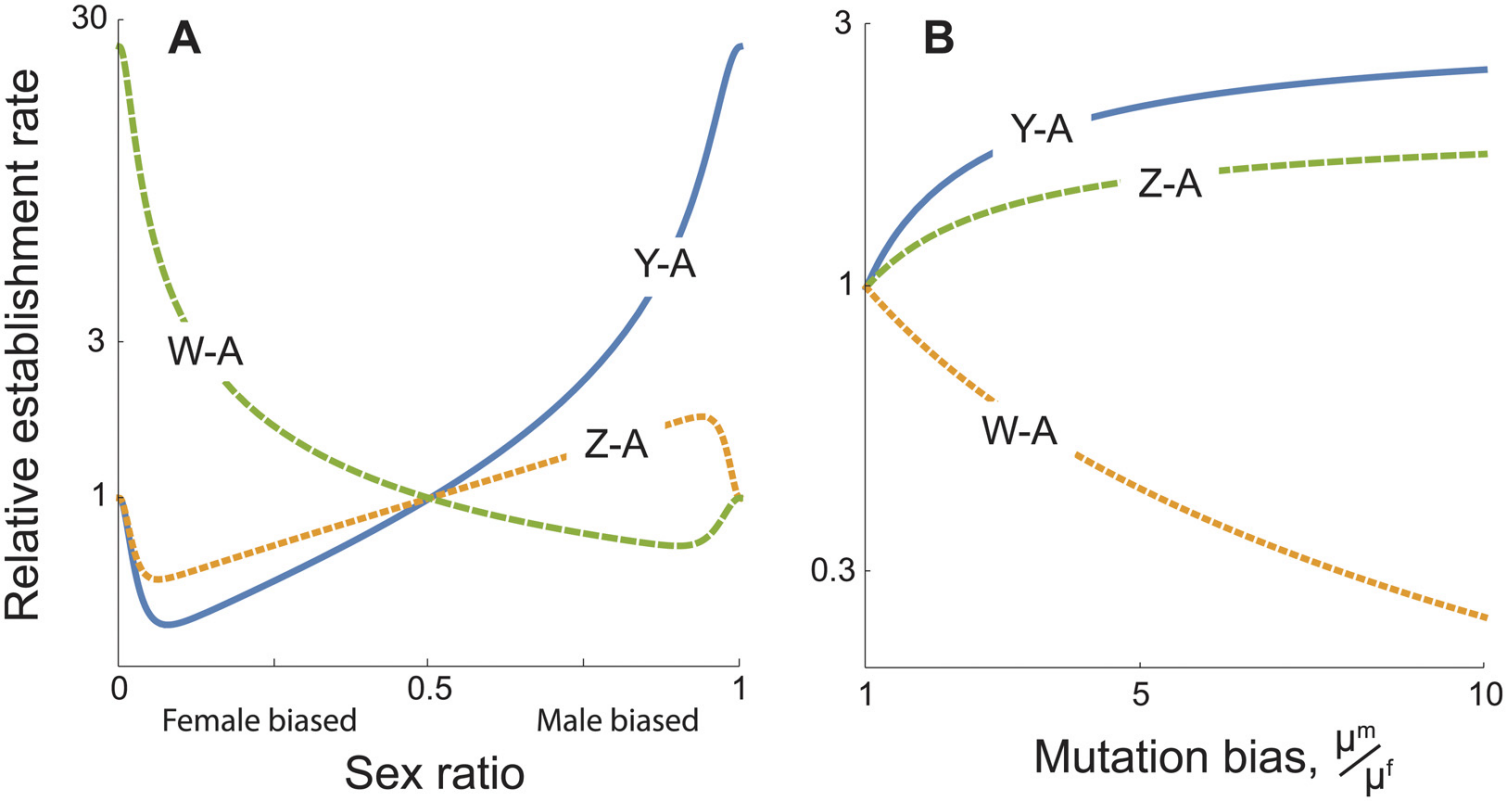
Establishment rates of sex chromosome-autosome fusions as a result of sexually antagonistic selection, relative to the rate for X-A fusions. The fusion is assumed to be neutral except for the effects of the sexually antagonistic allele that it captures. The fittest allele in each sex has a 10% advantage when homozygous and a 9% advantage when heterozygous (results are robust to these exact numbers). (*A*) Effect of sex ratio bias among reproductive adults, *N* ^*m*^ / (*N* ^*f*^ + *N* ^*m*^), assuming *μ* ^*m*^ = *μ* ^*f*^. (*B*) Effect of the relative mutation rate for fusions in males versus females, *μ* ^*m*^ / *μ* ^*f*^, assuming *N* ^*f*^ = *N* ^*m*^. Parameters: *N* ^*f*^ + *N* ^*m*^ = 10000.

To explain the observed excess of Y-A fusions by sexually antagonistic selection thus requires that the sexes differ in the mutation rate of fusions and/or in reproductive sex ratio (eq. A11). Again, Y-A fusions will be particularly common if fusions originate more frequently in males. If the mutation rates are equal in males and females, however, then Y-A fusions will only be more common than X-A fusions if the reproductive sex ratio is male-biased (that is, more males than females reproduce), which is atypical. These conditions are illustrated in Fig 6. In general, if there is a combination of sex-biased mutation rates and biased reproductive sex ratios, Y-A fusions become established most frequently due to sexually antagonistic selection as long as *μ* ^*m*^ *N* ^*m*^ > *μ* ^*f*^ *N* ^*f*^, where *μ* ^*f*^ and *μ* ^*m*^ are the female and male mutation rates, and *N* ^*f*^ and *N* ^*m*^ are the effective population sizes of females and males. When this condition is met, fusions also arise more often in XY lineages than in ZW lineages.

## Discussion

### Sex chromosome-autosome fusions are Y-biased in fishes and squamate reptiles

A major finding in our study is that Y-autosome fusions occur more frequently than other sex chromosome fusions in vertebrates, particularly in fishes and squamate reptiles. In amphibians, only one species in the database has multiple sex chromosomes, and it involves a Y-A fusion (Table 1). Because mammals and birds have only male heterogametic (XY) and female heterogametic (ZW) systems, respectively, we cannot use these taxa to conduct phylogenetic tests of the association between fusions and XY or ZW systems. We note, however, that there are many more known mammalian species with fusions, but only three avian species (Table 1). These data are consistent with our conclusion that fusions occur at a higher rate in XY than in ZW lineages.

Interestingly, however, mammals have roughly as many species with X-A fusions as with Y-A fusions. This suggests that evolutionary forces acting on fusions in mammals may be different from those in fish and reptiles. In particular, the form of female meiotic drive appears to vary among mammals, with drive favoring fused chromosomes in some species and unfused chromosomes in [26,27]. This leads to a pattern in which species with X-A fusions tend to have metacentric chromosomes (i.e., drive generally favors fused chromosomes), while species with Y-A fusions tend to have acrocentric chromosomes (i.e., drive generally favors unfused chromosomes) [21]. It is necessary to further examine the correlation between the frequencies of acrocentric (or metacentric) chromosomes and the types of fusions in many taxa.

Invertebrates provide a promising system for further phylogenetic analyses, with sex chromosome variation in several groups [2,13,17,38]. In Diptera there are seven ZW species 986 XY species, and 42 XO species in the Tree of Sex database [17]. Among these, there is a preponderance of fusions involving the Y: six Y-A fusions, one X-A fusion, and one species with both. Looking across all the invertebrates in the Tree of Sex database, there are many more cases of Y-A fusions (247 species) than X-A fusions (32 species), W-A fusions (8 species), and Z-A fusions (4 species); an additional 69 species have both X-A and Y-A fusions. While these data are consistent with the idea that Y-A fusions establish at a higher rate among invertebrates, a proper phylogenetic analysis is needed. A recent analysis of jumping spiders found only Y-A fusions (involving between four and seven independent events) among species that had both X and Y chromosomes [2,22]. Several X-A fusions were also identified, but these occurred only in species lacking a Y. Similar analyses in other groups of invertebrates promise to shed more light on sex chromosome evolution.

### Accounting for the high rate of Y-A fusions

Our theoretical analyses clarify the conditions under which fusions involving the Y chromosome are more likely to become established. Interestingly, several plausible explanations fail to account for the data. Neutral fusions could account for an excess of Y-A over X-A fusions if fusions arise more often in males, but under such conditions the theory predicts that Z-A fusions should also be common, which contradicts the data (Table 1, Figs 2 and 3). Likewise, beneficial fusions cannot explain the data, as they would tend to favor the accumulation of fusions involving the X or Z, which provide more abundant targets for new fusions than the Y or W. Furthermore, hypotheses in which fusions are established because they capture sexually antagonistic alleles also fail, because the smaller population sizes of Y and W sex chromosomes decreases the rate at which these types of fusions arise, counterbalancing the advantage they gain when capturing sexually antagonistic alleles. To account for the preponderance of Y-A fusions thus requires more complicated explanations, involving both selection and sex biases. We consider three plausible explanations below.

#### Deleterious fusions with a sex biased mutation rate or reproductive sex ratio

Chromosomal fusions may often have deleterious effects because fusions can lead to the loss of genetic material, alter gene expression, or increase the rate of segregation errors [18,23]. Because the Y and W chromosomes have smaller effective population sizes than Z and W chromosomes, deleterious Y-A and W-A fusions are expected to fix more frequently than deleterious X-A and Z-A fusions.

To account for the excess of Y-A over W-A fusions, however, requires some sort of sex bias. One promising candidate is sexual selection, which often increases the variance in reproductive success of males relative to females (Bateman’s principle) [35]. If fewer males than females reproduce successfully, the effective population size would be further reduced for the Y (but not for the W, carried by females) [10,39]. As a consequence, we expect Y-A fusions to be even more frequent in polygynous mating systems (Fig 5A).

Another promising candidate is a male-biased mutation rate. Studies in humans suggest that reciprocal translocations, a common route to fusions, are more often of paternal origin than maternal [30–32]. That said, Robertsonian fusions (a translocation between two acrocentric chromosomes resulting in a fused metacentric chromosome) are more often maternal in origin [40,41], but this pattern may be confounded by female meiotic drive favoring the transmission of metacentric fusions in humans [26]. While data from other species is needed, a preponderance of Y-A fusions can be explained if fusions primarily have slightly deleterious effects and also arise more often in males (Fig 5C). Of the three hypotheses we propose here, therefore, this appears to be most likely, given that the required conditions may be more often found in nature than those required for the other explanations as described below.

#### Meiotic drive

Because meiotic drive is often sex specific, it can break the symmetry between Y-A and W-A chromosomes and account for the high frequency of Y-A fusions. To do so requires female meiotic drive that selects against fused chromosomes, eliminating Z-A, W-A, and X-A fusions as they pass through female meiosis. Several cases of meiotic drive against fused chromosomes have been reported in mammals, for example in mice [26,27]. On the other hand, female meiotic drive favors fused chromosomes in humans [26], while male meiotic drive favors fused chromosomes in the common shrew [28,29].

Because the nature of meiotic drive varies among taxa, it seems unlikely that one particular form—female meiotic drive against fusions—is sufficiently widespread to explain the preponderance of Y-A fusions across vertebrates, particularly among fish (Fig 2) and squamate reptiles (Fig 3). Nevertheless, meiotic drive likely plays an important role in some taxa and may underlie the variation among mammals in rates of X-A and Y-A fusions [21].

#### Sexually antagonistic selection with a sex-biased mutation rate

Sexually antagonistic selection is generally considered a key evolutionary factor in the turnover of sex chromosomes [25,42,43]. Our models, however, indicate that fusions involving the Y will be no more common than those involving other sex chromosomes once we take into consideration the rate that Y fusions appear in the population and the fitness they gain by capturing a male-beneficial allele. In order to break the symmetry, we must again invoke either a male-biased mutation rate and/or a biased reproductive sex ratio. In this case, however, the sex ratio must be male-biased. That will cause less drift among males than females and so establish Y-A fusions more frequently than W-A fusions. Sexual selection, however, typically generates the opposite sex ratio bias. Consequently, sexually antagonistic selection requires even stronger male-biased mutation to explain the preponderance of Y-A fusions, compared to an explanation based on deleterious fusions.

### Other considerations

Other evolutionary forces not considered in this study may be important to the evolution of sex chromosome-autosome fusions. For example, we ignored inbreeding and spatial structure in our models. We also did not consider fusions that capture alleles held polymorphic by heterozygote advantage, but the fate of fusions is unaffected by such loci [25] unless there is inbreeding [44]. Furthermore, it is plausible that fusions may be more likely to involve some sex chromosomes for reasons that are independent of sex. For example, Y and W chromosomes often accumulate repetitive elements [13,38], which could make them more prone to fusion through nonhomologous recombination. X-A and Z-A fusions may also appear more ephemeral because the neo-Y and neo-W chromosomes that they generate could be lost without substantial fitness reductions due to masking in the hemizygous sex, leading to a loss of the multiple sex chromosome systems that we have used to detect fusions.

Alternatively, the Y and W may be less likely to be captured by a fusion when they are diminutive in size relative to the X and Z. Similarly, direct selection on fusions may be chromosome specific. For example, deletions and changes to gene expression may be less problematic on degenerated Y and Z chromosomes. While our analytical results allow for mutation rates and fitness effects to depend on the specific chromosome involved (S1 Text), our figures and conclusions were drawn assuming that there were only sex-specific and not chromosome-specific effects. As more data emerge about chromosome-specific mutation rates and selection, the analytical results can guide refinements to these conclusions.

## Methods

### Analysis of patterns of sex chromosome-autosome fusions in vertebrates

We compiled lists of species with multiple sex chromosome systems (X_1_X_2_Y, XY_1_Y_2_, ZW_1_W_2_, and Z_1_Z_2_W systems) from the Tree of Sex database [17]. Although X_1_X_2_Y systems (or ZW_1_W_2_ systems) can also arise from species with XO (or ZO) systems through a reciprocal translocation between an X (or a Z) and an autosome [2,20], XO or ZO systems are rare in vertebrates [17] (Table 1). In addition, although fission of sex chromosomes can also create multiple sex chromosome systems [2,20], such fissions are also rare in vertebrates [18,20,21]. We therefore focus this discussion on fusions, although the data analysis allowed fissions as well as fusions (S1 Text).

We address two questions with our empirical analyses. First, do Y-A (W-A) fusions occur at different rates than X-A (Z-A) fusions? Second, are there differences in rates of fusion between male and female heterogametic lineages? For both questions, we first simply tabulated the numbers in the database and computed Fisher’s exact test. This ignores phylogenetic non-independence but allowed us to use all of the available data.

To gain a better estimate of the rates at which fusions with different chromosomes get established, we fit phylogenetic models to the fusion data. We first matched sex chromosome systems from the fish dataset to a recent time-calibrated phylogeny of teleosts [45], containing 7811 species (we note that a small number of species were removed from the published phylogeny due to errors discovered after publication; M. Alfaro, personal communication). We matched the data of sex chromosome systems from squamates to the squamate phylogeny [46,47] using genetic data from 4161 species. In order to maximize overlap between the trait data and the species, we used an approximate matching algorithm for unmatched species: 1) retain all species that occur in both the tree and the dataset; 2) replace an unmatched species in the tree with a randomly selected unmatched species in the dataset from the same genus as long as this did not result in more than two representatives from the genus (this assumes monophyly of genera but avoids determining node order for nodes not in the original trees). We then pruned down the phylogeny down to those tips with data assignments.

In a first set of analyses, we fit a four-state Markov model (following [48]): 1) male heterogametic unfused; 2) male heterogametic fused; 3) female heterogametic unfused; 4) female heterogametic fused. We assumed that the probability of a fusion or fission event did not depend on whether the sex chromosomes were highly differentiated (heteromorphic) or not (homomorphic). To reduce model complexity, we first identified parameters for which little information exists in the data and that are similar biologically to other model parameters. We then used likelihood ratio tests to determine whether keeping these parameters distinct significantly improved the likelihood of the observed data (see S1 Text for details).

We fit the best supported models using a MCMC approach, as implemented in the diversitree R package [49], to estimate the posterior probability that XY fusions occurred at a greater rate of ZW fusions. We set broad exponential priors on all parameters (mean = 0.05). We ran the MCMC for 50,000 generations and removed the first 10,000 for burn-in. To accommodate auto-correlation between parameters, we calculated the difference between the rate of XY fusion and ZW fusion across the posterior distribution.

In a second set of analyses, we repeated these procedures, considering X-A, Y-A, Z-A, and W-A fusions separately. Code to reproduce all empirical analyses is available at https://github.com/mwpennell/fuse.

## Supporting Information

S1 Text Details of theoretical and phylogenetic analyses. (PDF)

S2 Text PDF file of supplementary *Mathematica* file of theoretical analysis. (PDF)

S3 Text Supplementary *Mathematica* file of theoretical analysis. (NB)

S1 Fig Fusion rate differences between XY and ZW systems (alternate model). Posterior estimate of the rate difference between XY and ZW fusions (*q*_*XY*.*XYF*_—*q*_*ZW*.*ZWF*_) in squamate reptiles when we allow the fission rates *q*_*XYF*.*XY*_ and *q*_ZWF.ZW_ to differ is shown.
(PDF)

S2 Fig Fusion residency time in squamates. Posterior estimate of the rate difference between XY and ZW fusions (*q*_*XY*.*XYF*_—*q*_*ZW*.*ZWF*_) in squamate reptiles when we allow the fission rates *q*_*XYF*.*XY*_ and *q*_*ZWF*.*ZW*_ to differ is shown.
(PDF)

S3 Fig Comparison of Y-autosome and X-/Z-autosome fusion rates (fish). Posterior estimate of the rate difference between YA and XA/ZA fusions in fish is shown. When the estimate is greater than zero, this means that the YA fusion rates are higher than those of the other chromosomes.
(PDF)

S4 Fig Comparison of Y-autosome and W-/Z-autosome fusion rates (squamates). Posterior estimate of the rate difference between YA and WA/ZA fusions in squamate reptiles is shown. When the estimate is greater than zero, this means that the YA fusion rates are higher than those of the other chromosomes.
(PDF)
