# Supplementary material for: Y Fuse? Sex Chromosome Fusions in Fishes and Reptiles
Source: PLoS Genet. 2015 May 20;11(5):e1005237. doi: 10.1371/journal.pgen.1005237 (PMC4439076; doi:10.1371/journal.pgen.1005237)
Supplement: S1 Text — (PDF) [file pgen.1005237.s001.pdf]

APPENDICES FOR

Y fuse? Sex chromosome fusions in fishes and reptiles

Matthew W. Pennell[†,1], Mark Kirkpatrick[†,2], Sarah P. Otto[†,3], Jana C. Vamosi[4], Catherine L. Peichel[5], Nicole Valenzuela[6], and Jun Kitano[7,*]

† *These authors contributed equally*
1 *Institute for Bioinformatics and Evolutionary Studies, University of Idaho, Moscow, ID 83844, USA*
2 *Department of Integrative Biology, University of Texas, Austin, TX 78712, USA*
3 *Department of Zoology, University of British Columbia, Vancouver, BC V6J 3S7, Canada*
4 *Department of Biological Sciences, University of Calgary, Calgary, AB T2N 1N4, Canada*
5 *Divison of Human Biology, Fred Hutchinson Cancer Research Center, Seattle, WA 98109, USA*
6 *Department of Ecology, Evolution and Organismal Biology, Iowa State University, Ames, IA 50011, USA*
7 *Ecological Genetics Laboratory, National Institute of Genetics, Mishima, Shizuoka 411-8540, Japan*

*jkitano@lab.nig.ac.jp

APPENDIX 1: DETAILS OF THEORETICAL ANALYSES

Here we describe the theoretical models discussed in the main text. Full details of the derivations as well as further exploration of model assumptions can be found in the supplemental *Mathematica* notebook. We consider two main types of models: those that include direct selection on fusions and those that include sexually antagonistic selection. The direct selection models can also be used to consider the special cases of neutral evolution, weak selection, and meiotic drive. For all cases we allow for the possibility of differences between chromosomes in both effective population sizes and mutation rates. Our approach follows standard procedures in population genetics for calculating the rate of establishment as the product of appearance rates and fixation rates (see Charlesworth et al., 1987; Charlesworth, 1994; Kirkpatrick and Hall, 2004).

Direct selection

We track the rate of appearance and fixation of a sex-autosome fusion, where the rate at which mutation generates a fusion between a sex chromosome and an autosome is μ_C^{sex} per gamete per generation for chromosome C ($C = X, Y, Z,$ or W) in males ($sex = m$) and females ($sex = f$). We assume that, at birth, the population is of constant total size N, consisting of an equal number ($N/2$) of males and females. Not all of these individuals survive and many will be unable to attract a mate or breed, particularly in the presence of strong sexual selection. Considering only individuals that survive both natural and sexual selection, there are N^f females and N^m males, where we assume that the successful females or males are equivalent and expected to have a Poisson distributed

number of offspring (we refer to these as "reproductive" individuals, although technically some may still fail to leave offspring by chance, as described by the Poisson distribution). We assume throughout that N^f and N^m are sufficiently large and stable that we can ignore stochasticity in the sex ratio.

The effective population sizes of Y and W chromosomes are then $N_{e,Y} = N^m$ and $N_{e,W} = N^f$, respectively, while the effective population sizes of X and Z chromosomes equal:

$$N_{e,X} = \frac{9N^f N^m}{N^f + 2N^m} \tag{A.1a}$$

$$N_{e,Z} = \frac{9N^f N^m}{2N^f + N^m} \tag{A.1b}$$

(Wright, 1933; see also Caballero, 1995 and Laporte and Charlesworth, 2002, for extensions to non-Poisson distributions). Note that the above equations define the effective number of chromosomes, not the effective number of individuals.

Once the fusion appears, we approximate its fixation rate using Kimura's (1962) diffusion approximation. In the supplemental *Mathematica* notebook, we allow for arbitrary levels of dominance of the fusion (including underdominance). Dominance has little effect on which type of fusion is expected to become established most frequently. Hence, we focus here on the simpler additive case, where the fixation probability of a fusion is:

$$P_C = \frac{1 - \exp[-2s_C N_{e,C} p]}{1 - \exp[-2N_{e,C} s_C]} \tag{A.2}$$

where s_C is the selection coefficient acting directly upon individuals carrying the fusion when rare (as heterozygotes), p is the initial frequency of the fusion, and $N_{e,C}$ is the relevant effective population size of the chromosome C. (Recall that $N_{e,C}$ is the effective number of chromosomes, not individuals, which is why '2' rather than the standard '4' for diploids appears in Equation A.2.) We also assume that selection on the fusion is sufficiently weak that the selection coefficient can be taken as the average over many generations, accounting for the time spent in each sex:

$$s_X = \frac{2}{3}s_X^f + \frac{1}{3}s_X^m \tag{A.3a}$$

$$s_Y = s_Y^m \tag{A.3b}$$

$$s_Z = \frac{1}{3}s_Z^f + \frac{2}{3}s_Z^m \tag{A.3c}$$

$$s_W = s_W^f \tag{A.3d}$$

Below, we consider both the rate at which fusions originate and the rate at which they fix, for fusions involving different sex chromosomes.

Y-A FUSIONS — Y-A fusions appear in the population at rate $\frac{N}{2}\mu_Y^m$. The probability that the fusion fixes is the chance that the fusion survives natural and sexual selection in the first generation to be present among the adult males of the population, $N^m/(N/2)$, times the probability that the fusion will be the ultimate ancestor of the Y chromosomes among the descendants after some long period of time, given by (A.2) for the $C = Y$ chromosome with $N_{e,Y} = N^m$ and $p = 1/N^m$. Multiplying the mutation rate by the fixation probability, the overall establishment probability for a Y-A fusion is

$$\begin{aligned} R_Y &= N^m \mu_Y^m P_Y \\ &= N^m \mu_Y^m \frac{1 - \exp[-2s_Y]}{1 - \exp[-2N^m s_Y]} \end{aligned} \tag{A.4}$$

We note that (A.4) is a standard result for the establishment of a mutation in a haploid model (e.g., Charlesworth et al., 1987; Kirkpatrick and Hall, 2004), although we have used (A.2) to consider the the fixation probability of either beneficial or deleterious fusions and have used additional subscripts and superscripts to allow us to consider sex biased mutation rates and reproductive sex ratios. For (A.4) and the following equations, the establishment rate does not depend on the population size at birth (N), at least not to leading order; the same is true—N cancels—if we allow the sex ratio at birth to be biased rather than set equal to $N/2$.

X-A FUSIONS — X-A fusions appear in the population at rate $2\frac{N}{2}\mu_X^f$ among females and at rate $\frac{N}{2}\mu_X^m$ among males, where the former expression accounts for the fact that females carry two X chromosomes. A fusion arising in a female has a chance $N^f/\frac{N}{2}$ of surviving to reproduce. The probability that the fusion will be the ultimate ancestor of the X chromosomes after some long period of time is then given by (A.2) for $C = X$, with $N_{e,X}$ given by (A.1a) and $p = \frac{2}{3}/(2N^f)$ accounting for the fact that $\frac{2}{3}$ of the X chromosomes in the next generation come from these mothers, among whom the fusion is at initial frequency $1/(2N^f)$. A similar calculation applies to males, so that the net establishment rate is approximately:

$$R_X = 2N^f \mu_X^f \frac{1 - \exp[-2s_X N_{e,X} \frac{2}{3}\frac{1}{2N^f}]}{1 - \exp[-2N_{e,X} s_X]} + N^m \mu_X^m \frac{1 - \exp[-2s_X N_{e,X} \frac{1}{3}\frac{1}{N^m}]}{1 - \exp[-2N_{e,X} s_X]} \tag{A.5}$$

W-A FUSIONS — The establishment rate of W-A fusions, R_W, is derived as for Y-A fusions, giving (A.4) but with m replaced by f and Y replaced by W.

Z-A FUSIONS — The establishment rate of Z-A fusions, R_Z, is derived as for X-A fusions, giving (A.5) but with m and f interchanged and X replaced by Z.

Neutral fusions — When selection is negligible, the above formulae can be simplified substantially. In the limit for neutral fusions ($s_C = 0$), the net establishment rate equals the rate at which each type of fusion arises:

$$R_Y = \mu_Y, \tag{A.6a}$$

$$R_X = \frac{2}{3}\mu_X^f + \frac{1}{3}\mu_X^m, \tag{A.6b}$$

$$R_W = \mu_W, \tag{A.6c}$$

$$R_Z = \frac{2}{3}\mu_Z^m + \frac{1}{3}\mu_Z^f. \tag{A.6d}$$

We note that these are also standard results (e.g., Kirkpatrick and Hall, 2004; Vicoso and Charlesworth, 2009). Observe that the reproductive population sizes of males (N^m) and females (N^f) are irrelevant to the relative rate of fusion establishment when there is no direct selection on the fusions. A neutral fusion is less likely to survive and reproduce if it first appears in the sex with the lower reproductive population size, but if it does, then it has a higher chance of being the progenitor chromosome; these effects exactly cancel out.

Weak selection — The relative establishment rates also simplify substantially when selection is very weak: $|\theta| << 1$, where $\theta = 4(N^m + N^f)s_C$. To leading order in θ, the establishment rate for each type of fusion, measured relative to the rate of X-A fusions, is:

$$\frac{R_Y}{R_X} = \frac{3\alpha}{2+\alpha}\left(1 + \theta\frac{1-4\gamma}{2(1+\gamma)(2+\gamma)}\right), \tag{A.7a}$$

$$\frac{R_W}{R_X} = \frac{3}{2+\alpha}\left(1 - \theta\frac{\gamma(7-\gamma)}{4(1+\gamma)(2+\gamma)}\right), \tag{A.7b}$$

$$\frac{R_Z}{R_X} = \frac{2\alpha+1}{2+\alpha}\left(1 + \theta\frac{9\gamma(1-\gamma)}{4(1+\gamma)(2+\gamma)(1+2\gamma)}\right), \tag{A.7c}$$

where fusions arise in males at a rate $\alpha = \mu^m/\mu^f$ times that in females and the number of reproductive females is $\gamma = N^f/N^m$ times the number of males (so that the sex ratio $N^m/(N^m + N^f) = 1/(\gamma+1)$). In the absence of a sex bias in the mutation rate ($\alpha = 1$) or number of reproductive individuals ($\gamma = 1$), we find that

$$\frac{R_Y}{R_X} = \frac{R_W}{R_X} = 1 - \frac{\theta}{4}$$

and

$$\frac{R_Z}{R_X} = 1.$$

This confirms that direct selection alone cannot explain the predominance of Y-A fusions, because we would expect W-A fusions to establish at similar rates.

Similarly, the overall rate at which fusions arise in XY systems versus ZW systems is the sum of the rates for the component chromosomes, keeping only leading order terms in θ:

$$\frac{R_X + R_Y}{R_Z + R_W} = \frac{1+2\alpha}{\alpha+2} + \frac{\theta\gamma}{(1+\gamma)}\left[\left(\frac{3\alpha}{2+\alpha}\right)\left(\frac{1-4\gamma}{4\gamma(2+\gamma)}\right) + \left(\frac{3(1+2\alpha)}{(2+\alpha)^2}\right)\left(\frac{7-\gamma}{8(2+\gamma)}\right) - \left(\frac{(1+2\alpha)^2}{(2+\alpha)^2}\right)\left(\frac{9(1-\gamma)}{8(2+\gamma)(1+2\gamma)}\right)\right]. \quad \text{(A.8)}$$

Sex-antagonistic selection

Consider an autosomal locus with selection acting in opposite directions in males and females, with allele A_0 favored in males and allele A_1 in females. If selection is weak, the allele frequency q_i of allele A_i is approximately the same in males and females. Given the sex-specific fitness of genotype ij, W_{ij}^{sex}, we can then define the selection coefficient favoring allele A_i in a particular sex as $s_i^{sex} = (W_{i.}^{sex}/\overline{W}^{sex}) - 1$. Here $W_{i.}^{sex}$ is the marginal fitness of A_i in that sex ($W_{i.}^{sex} = q_0 W_{i0}^{sex} + q_1 W_{i1}^{sex}$), and $\overline{W}^{sex}$ is the mean fitness ($\overline{W}^{sex} = q_0 W_{0.} + q_1 W_{1.}$).

Following similar logic used to derive equations (A.4) and (A.5), fusions bearing allele A_i arise with the Y chromosome and are found in a reproductive male at rate $q_i \mu_Y^m N^m$ or arise with the W and are found in a reproductive female at rate $q_i \mu_W^f N^f$. Similarly, the rate at which X-A fusions or Z-A fusions bearing allele A_i originate is $q_i(2\mu_X^f N^f + \mu_X^m N^m)$ or $q_i(\mu_Z^f N^f + 2\mu_Z^m N^m)$, respectively. If we assume selection is weak, we can average over the time the chromosome spends in a female and a male to obtain the strength of selection acting on a fusion bearing allele A_i: $s_{X,i} = \frac{2}{3}s_i^f + \frac{1}{3}s_i^m$ for an X-A fusion, $s_{Y,i} = s_i^m$ for a Y-A fusion, $s_{Z,i} = \frac{1}{3}s_i^f + \frac{2}{3}s_i^m$ for a Z-A fusion, and $s_{W,i} = s_i^f$ for a W-A fusion.

Because the X and W are more often found in females, the fixation probability of an X-A or W-A fusion is much higher if it captures the female-benefit allele A_1 than if it captures the male-benefit allele (and *vice versa* for Y-A and Z-A fusions). Using $2s_C N_{e,C} p$ to approximate the fixation probability (A.2) for a beneficial fusion initially at frequency p, the fixation probability of an X-A fusion is approximately $P_X = 2s_{X,1}N_{e,X}p$ when it captures allele A_1 and zero otherwise. Similarly, $P_W = 2s_{W,1}N_{e,W}p$ when a W-A fusion captures A_1, $P_Y = 2s_{Y,0}N_{e,Y}p$ when a Y-A fusion captures A_0, and $P_Z = 2s_{Z,0}N_{e,Z}p$ when a Z-A fusion captures A_0.

Multiplying together the rate that fusions originate in each sex times their fixation probability (accounting for the initial frequency in that sex), we get the rate at which fusions are expected to become established for each sex chromosome:

$$R_Y = q_0 \mu_Y N^m \left(2 s_0^m\right), \tag{A.9a}$$

$$R_X = 2 q_1 \frac{9 N^f N^m}{N^f + 2N^m} \left(\frac{2}{3}\mu_X^f + \frac{1}{3}\mu_X^m\right)\left(\frac{2}{3}s_1^f + \frac{1}{3}s_1^m\right), \tag{A.9b}$$

$$R_W = q_1 \mu_W N^f \left(2 s_1^f\right), \tag{A.9c}$$

$$R_Z = 2 q_0 \frac{9 N^f N^m}{2N^f + N^m} \left(\frac{1}{3}\mu_Z^f + \frac{2}{3}\mu_Z^m\right)\left(\frac{1}{3}s_0^f + \frac{2}{3}s_0^m\right). \tag{A.9d}$$

At an autosomal locus subject to sexually antagonistic selection, each allele has spent half of its time in males and half in females, rising in frequency in one sex and falling in the other sex. Consequently, to remain at equilibrium over the longer term, the selection coefficients for each allele as measured at this equilibrium must balance across the sexes, with $s_0^f = -s_0^m$ and $s_1^f = -s_1^m$ (see formal proof in the supplemental *Mathematica* notebook). Furthermore, the fitness definitions imply that $q_0 s_0^{sex} + q_1 s_1^{sex}$ must equal zero:

$$q_0 s_0^{sex} + q_1 s_1^{sex} = \frac{q_0 W_{0.}^{sex} + q_1 W_{1.}^{sex}}{\overline{W}^{sex}} - 1 = \frac{\overline{W}^{sex}}{\overline{W}^{sex}} - 1 = 0.$$

Using these relationships to substitute for s_i^f and q_1, we find:

$$R_Y = 2 s_0^m q_0 (\mu_Y N^m), \tag{A.10a}$$

$$R_X = 2 s_0^m q_0 \left(\frac{(2\mu_X^f + \mu_X^m) N^f N^m}{N^f + 2N^m}\right), \tag{A.10b}$$

$$R_W = 2 s_0^m q_0 (\mu_W N^f), \tag{A.10c}$$

$$R_Z = 2 s_0^m q_0 \left(\frac{(\mu_Z^f + 2\mu_Z^f) N^f N^m}{2N^f + N^m}\right). \tag{A.10d}$$

Thus, with equal mutation rates and equal numbers of reproductive individuals of the two sexes, the establishment rates all equal one another. Otherwise, recalling that $\alpha = \mu^m/\mu^f$ and $\gamma = N^f/N^m$, the establishment rates relative to the rate of X-A fusions become:

$$\frac{R_Y}{R_X} = \frac{\alpha(2+\gamma)}{\gamma(2+\alpha)}, \tag{A.11a}$$

$$\frac{R_W}{R_X} = \frac{2+\gamma}{2+\alpha}, \tag{A.11b}$$

$$\frac{R_Z}{R_X} = \frac{(1+2\alpha)(2+\gamma)}{(1+2\gamma)(2+\alpha)}, \tag{A.11c}$$

Consequently, Y-A fusions are expected to predominate (with $R_Y > \max[R_X, R_W, R_Z]$) if and only if $\alpha > \gamma$.

APPENDIX 2: DETAILS OF PHYLOGENETIC ANALYSES

To investigate the relative rates of different types of fusions across our two focal groups–teleost fishes and squamate reptiles–we fit multiple phylogenetic models to our karyotype dataset. We first matched the available karyotype data to the fish (Rabosky et al., 2013) and squamate (Pyron et al., 2013; Pyron and Burbrink, 2014) phylogenies (using an approximate matching algorithm described in the main text). This resulted in phylogenetic comparative datasets containing 163 species of fish and 261 squamate species. We conducted two separate types of analyses on both groups. First, we examined differences between XY and ZW systems; here, we treat X-autosome and Y-autosome fusions as equivalent (see below), and likewise, Z-autosome and W-autosome fusions. Results from this first analysis are presented in the main text. Second, we investigated autosomal fusion rates for all types of sex chromosomes individually (i.e., Y-, X-, W-, and Z-autosome fusions). While the second analysis provides more detailed resolution, some of the states are rarely observed (and in some cases, not at all). All analyses were performed using the R package DIVERSITREE (FitzJohn, 2012), and code to reproduce all results can be found at `https://github.com/mwpennell/fuse/analysis`.

Fusion rates in XY vs. ZW systems

Using a Markov model (Pagel, 1994), we considered transitions among the following states:

- XY: Male heterogametic unfused
- XY_F: Male heterogametic fused (XXY or XYY)
- ZW: Female heterogametic unfused
- ZW_F: Female heterogametic fused (ZZW or ZWW)

allowing transitions between all states with $q_{A.B}$ representing the transition rate between states A and B. We then used likelihood ratio tests to restrict the model in order to improve our ability to estimate the parameters of interest.

We first imposed the biologically reasonable constraint that prior to becoming XY_F (or ZW_F), a lineage must first be XY (or ZW); e.g., the transition rate from female heterogametic unfused to male heterogametic fused $q_{ZW.XY_F}$ would be zero. These restrictions did not lead to a significant decline in likelihood for either squamates or fish and was accepted.

Next, we proposed a model in which the rate of switching the heterogametic sex, going from a XY to a ZW system and *vice versa*, did not depend on whether the lineage contained a fused sex chromosome or not (e.g., $q_{XY_F.ZW} = q_{XY.ZW}$). In both fish and squamates, this restriction was acceptable using likelihood ratio tests.

In the next step, we proposed a model in which the rate of chromosomal fission, going from a fused sex chromosome system to an unfused system of the same type, was the same for XY and ZW systems. In fish, a likelihood ratio test favored the more restricted model, whereas in squamates, the more general model (where $q_{XY_F.XY} \neq q_{ZW_F.ZW}$) was favored ($p = 0.012$). The support for the more general model in squamates stems from the scarcity of ZW fusions in the data; there is little information to reliably estimate the transition rate from fused female heterogametic to unfused female heterogametic ($q_{ZW_F.ZW}$) using maximum likelihood (see below). We therefore took slightly different approaches when analyzing the two clades.

For fish, we compared the resulting model ($q_{XY_F.XY} = q_{ZW_F.ZW}, q_{ZW.XY_F} = q_{XY.ZW_F} = 0, q_{XY_F.ZW} = q_{XY_Z.ZW}, q_{ZW_F.XY} = q_{ZW.XY}$) to an even more reduced model in which the XY and ZW fusion rates were set to be equal ($q_{XY.XY_F} = q_{ZW.ZW_F}$). We found the rate difference to be highly significant ($p = 0.014$) using a likelihood ratio test. To better accomodate uncertainty in the estimate, we ran a Bayesian analysis (described in the text), and this too supported our conclusion that XY fusions occur at a higher rate than ZW fusions (98.6% of the posterior probability supported this and the 95% credibility interval for the difference in rates did not overlap with zero; Figure 4 in the main text).

For the squamate data, we took two approaches. First, we assumed that the 'equal fission rates model' was indeed reasonable and performed the same analysis as in fish. Using a likelihood ratio test, the difference in fusion rates for XY and ZW was found to be highly significant ($p = 0.003$). The same was true for the Bayesian analysis (99.9% of the posterior probability distribution supported this conclusion; Figure 4 in the main text). Second, we used a Bayesian MCMC to fit a model in which the fission rate $q_{ZW_F.ZW}$ was estimated independently of $q_{XY_F.XY}$. For this model the support for the difference between XY and ZW fusion rates was not as strong (92.0% of the posterior probability supported $q_{XY.XY_F} > q_{ZW.ZW_F}$; Figure A.1).

As mentioned above, the squamate data contain very little information about fission rates, especially from ZW_F to ZW. The likelihood approach has difficulty distinguishing between two explanations for the lack of fused ZW chromosomes: rare ZW fusions or common ZW fissions. Nevertheless, there is a strong signal that ZW fusions should be less common, which we confirmed by considering residency times t_R, the average

evolutionary duration of a fused state. For XY fusions,

$$t_{R,XY_F} = \frac{q_{XY.XY_F}}{q_{XY.XY_F} + q_{XY_F.XY}} \tag{A.12}$$

and for ZW fusions

$$t_{R,ZW_F} = \frac{q_{ZW.ZW_F}}{q_{ZW.ZW_F} + q_{ZW_F.ZW}} \tag{A.13}$$

Using a Bayesian analysis, we found very strong support for the residency time being greater for XY fusions than ZW fusions (99.8% of the posterior probability supported $t_{R,XY_F} > t_{R,ZW_F}$; Figure A.2). In the absence of direct information about fission rates for fused ZW chromosomes, we conclude that the data is more parsimoniously explained by rare ZW fusions, while acknowledging that rapid ZW fission rates may also explain the data for squamates.

Comparing fusion rates between chromosomes

Rather than classifying the states as male/female heterogametic unfused/fused, we separated out the different types of fusions (e.g., classifying X-autosome [XA] and Y-autosome [YA] fusions as different states). This allowed us to assess whether the patterns we observed were driven by an overabundance of autosomal fusions with the Y chromosome. After matching the data to the tree, we did not have any records of WA fusions in fish while in squamates, XA fusions were absent. We thus considered models with only three fused states (for fish: XA, YA, and ZA; for squamates: YA, WA, and ZA)

For both the fish and the squamates, we again restricted the model via a nested series of likelihood ratio tests. For both clades, we found it to be statistically justifiable to assume that: a) transitions from one fused state directly to another fused state were impossible; b) prior to becoming fused, a lineage had to be in the corresponding unfused state; and c) fission rates were constrained to be equal ($q_{XY_F.XY} = q_{ZW_F.ZW}$). This allowed us to reliably evaluate whether the fusion rates differed by chromosome.

For the fish, using likelihood ratio tests, we found YA fusions to be significantly higher than XA fusions ($p = 0.016$) and ZA fusions ($p = 0.035$), but that XA and ZA fusion rates were not significantly different ($p = 0.658$). Again, WA fusions did not exist in the fish analysis so we could not compare them to other classes. We then performed a Bayesian MCMC analysis to gain a better estimate of the relevant parameters. For the purposes of this analysis, we fixed XA and ZA fusions to occur at the same rate and then compared this rate to that for YA fusion. We found that YA fusions occur at a much higher rate than XA/ZA fusions (Figure A.3; 99.5% of the posterior distribution supported this conclusion).

For the squamate analysis, YA fusions also occured at a higher rate than WA fusions ($p < 0.001$) and ZA fusions ($p < 0.001$). WA and ZA fusions rates were not significantly different from one another ($p \approx 1$). As with the fish, for the Bayesian analysis we set WA and ZA fusion rates to be equal and estimated the difference between YA fusions

and other type of fusions. 99.9% of the posterior probability distribution supported YA fusions occuring at a higher rate than fusions on other chromosomes (Figure A.4).

Taken together, these results strongly suggest that the difference between XY and ZW fusion rates is driven almost entirely by the very high rates of autosomal fusions involving the Y chromosome relative to the other sex chromosomes.

REFERENCES

Caballero, A. 1995. On the effective size of populations with separate sexes, with particular refenc to sex-linked genes. Genetics 139:1007–1011.

Charlesworth, B. 1994. The effect of background selection against deleterious mutations on weakly selected, linked variants. Genetical research 63:213–227.

Charlesworth, B., J. A. Coyne, and N. H. Barton. 1987. The relative rates of evolution of sex chromosomes and autosomes. American Naturalist Pages 113–146.

FitzJohn, R. G. 2012. Diversitree: comparative phylogenetic analyses of diversification in R. Methods in Ecology and Evolution 3:1084–1092.

Kimura, M. 1962. On the probability of fixation of mutant genes in a population. Genetics 47:713–719.

Kirkpatrick, M. and D. W. Hall. 2004. Sexual selection and sex linkage. Evolution 58:683–691.

Laporte, V. and B. Charlesworth. 2002. Effective population size and population subdivision in demographically structured populations. Genetics 162:501–519.

Pagel, M. 1994. Detecting correlated evolution on phylogenies: A general method for the comparative analysis of discrete characters. Proceedings of the Royal Society of London. Series B: Biological Sciences 255:37–45.

Pyron, R. A. and F. T. Burbrink. 2014. Early origin of viviparity and multiple reversions to oviparity in squamate reptiles. Ecology Letters 17:13–21.

Pyron, R. A., F. T. Burbrink, and J. J. Wiens. 2013. A phylogeny and revised classification of squamata, including 4161 species of lizards and snakes. BMC Evolutionary Biology 13:93.

Rabosky, D. L., F. Santini, J. Eastman, S. A. Smith, B. Sidlauskus, J. Chang, and M. E. Alfaro. 2013. Rates of speciation and morphological evolution are correlated across the largest vertebrate radiation. Nature Communications 4:1958.

Vicoso, B. and B. Charlesworth. 2009. Effective population size and the faster-X effect: an extended model. Evolution 63:2413–2426.

Wright, S. 1933. Inbreeding and homozygosis. Proceedings of the National Academy of Sciences, USA 19:411–419.
