# Supplementary material for: Y Fuse? Sex Chromosome Fusions in Fishes and Reptiles
Source: PLoS Genet. 2015 May 20;11(5):e1005237. doi: 10.1371/journal.pgen.1005237 (PMC4439076; doi:10.1371/journal.pgen.1005237)
Supplement: S2 Text — (PDF) [file pgen.1005237.s002.pdf]

Sup. Mat.: Y fuse? Patterns of sex chromosome fusions in fishes and reptiles

Jun Kitano, Matthew W. Pennell, Mark Kirkpatrick, Sarah P. Otto, Jana C. Vamosi, and Catherine L. Peichel.

Neutral case

Introduction and derivation

Consider fusions that originate at rate μ_C^{sex} in sex (sex = m or f) and involving chromosome C (C = X, Y, Z, or W). We assume that there is no recombination between the sex determining region (SDR) and the fusion; that is, an X-A fusion is assumed to remain X-A and not recombine to create a fused Y-A chromosome. [Such recombination events would lead to expanded chromosome regions on both sex chromosomes, if established, which would not be detected in our data sets, where fusions are only seen if they remain linked to one sex chromosome, e.g., XY1Y2.] We also assume that the sex ratio at birth of males and females is 50:50, so that N/2 is the number of males or the number of females, where N is the total population size. Differences in survival and mating success, however, lead to different reproductive population sizes for males (N_m) and females (N_f) at the time of reproduction. What then is the establishment rate of each type of fusion?

YA: YA fusions originate at rate $\frac{N}{2}\mu_Y$ (we suppress the "sex" superscript, as these are always in males) in any one of the males born into the population. Eventually, the population will descend from one of these Y chromosomes, but which one is random in this neutral scenario. Thus, the probability of fixation is $1/\frac{N}{2}$, yielding an overall establishment rate of μ_Y. An alternative way of deriving the same result is to state that, of the $\frac{N}{2}$ males born, a fusion has a chance $\frac{N_m}{N/2}$ of surviving to reproduce and of being in the "effective population" of males; one of these reproducing males will be the ancestor of the Y's ultimately, giving an overall establishment rate of $\frac{N}{2}\mu_Y\frac{N_m}{N/2}\frac{1}{N_m}=\mu_Y$.

XA: XA fusions originate at rate $2\frac{N}{2}\mu_X^f$ in females (the "2" in front comes from the two X's in females) and $\frac{N}{2}\mu_X^m$ in males. Eventually, the population will descend from one of these X chromosomes, but which one is random in this neutral scenario. Thus, the probability of fixation is $1/\frac{3N}{2}$, yielding an overall establishment rate of $\frac{2}{3}\mu_X^f+\frac{1}{3}\mu_X^m$. An alternative way of deriving the same result is to state that, of the $\frac{N}{2}$ females born, a fusion has a chance $\frac{N_f}{N/2}$ of surviving to reproduce and of being in the "effective population" of females; as the contribution of fathers and mothers is equal, we know that 2/3 of the X's will eventually descend from the mothers of this generation and the chance that one of these will be the fusion is $\frac{2}{3}\frac{1}{2N_f}$ (similarly for males); thus, among the population of reproducing X chromosomes, one will be the ancestor of the X's ultimately, giving an overall establishment rate of $2\frac{N}{2}\mu_X^f\frac{N_f}{N/2}\frac{2}{3}\frac{1}{2N_f}+\frac{N}{2}\mu_X^m\frac{N_m}{N/2}\frac{1}{3}\frac{1}{N_m}=\frac{2}{3}\mu_X^f+\frac{1}{3}\mu_X^m$.

WA: WA fusions originate at rate $\frac{N}{2}\mu_W$ in any one of the females born into the population. Eventually, the population will descend from one of these W chromosomes, but which one is random in this neutral scenario. Thus, the probability of fixation is $1/\frac{N}{2}$, yielding an overall establishment rate of μ_W.

ZA: ZA fusions originate at rate $2\frac{N}{2}\mu_Z^m$ in males (the "2" in front comes from the two Z's in males) and $\frac{N}{2}\mu_X^f$ in females. Eventually, the population will descend from one of these Z chromosomes, but which one is random in this neutral scenario. Thus, the probability of fixation is $1/\frac{3N}{2}$, yielding an overall establishment rate of $\frac{2}{3}\mu_Z^m+\frac{1}{3}\mu_X^f$.

TOTAL: If we calculate the overall rate of fusions appearing in XY species versus ZW species, we get:

$$\frac{\frac{2}{3}\mu_X^f+\frac{1}{3}\mu_X^m+\mu_Y}{\mu_W+\frac{2}{3}\mu_Z^m+\frac{1}{3}\mu_X^f};$$

Assuming that sex determines the fusion rate, not the chromosome itself, the ratio becomes:

```
OriginBySex = {μX^f → μ^f, μX^m → μ^m, μY → μ^m, μZ^m → μ^m, μZ^f → μ^f, μW → μ^f} /. μ^m → α μ^f;
%% /. OriginBySex // Simplify
```

$$\frac{1+2\alpha}{2+\alpha}$$

This is counter-intuitive. Essentially, if mutations happen much more in males ($\alpha >> 1$), then XY fusions will be more common than ZW fusions even in the neutral case because a single fusion that happens in a male will have a higher chance of fixing on the Y than on the X or Z, because of the smaller number of Y chromosomes. Conversely, if mutations happen much more in females ($\alpha << 1$), then ZW fusions will be more common than XY fusions because a single fusion that happens in a female will have a higher chance of fixing on the W than on the X or Z, because of the smaller number of W chromosomes.

Direct fitness effects of fusions [additive case]

Introduction and derivation

We now modify the above to allow the fusion to have a direct fitness effect (often deleterious), which may be different in males and females. How does this affect the rate of establishment?

In the following we approximate this establishment rate using Kimura's (1962) diffusion approximation for the fixation probability of a mutation:

$$P = \frac{1-\mathrm{Exp}[-2\, s\, N_e\, p]}{1-\mathrm{Exp}[-2\, N_e\, s]}$$

where s is the selection coefficient, p is the initial frequency of a mutation (here a fusion), and N_e is the relevant effective population size of chromosomes (not of individuals). This assumes that either the allele is additive (with heterozygous effect s) or that the fixation probability is determined while the allele is rare (see next section for other cases). Given a number of reproducing females (N_f) and males (N_m) with an expected Poisson distributed number of offspring, the effective population sizes for X and Z chromosomes are:

$$N_{e,x} = \frac{9\, N_f\, N_m}{N_f + 2\, N_m};$$

$$N_{e,z} = \frac{9\, N_f\, N_m}{2\, N_f + N_m};$$

We also assume that the selection on the fusion is weak and can be taken as the average over many generations. Specifically, for X and Z chromosomes, we take selection to be the long-term averages:

$$s_X = \frac{1}{3} s_X^m + \frac{2}{3} s_X^f$$

$$s_Z = \frac{1}{3} s_Z^f + \frac{2}{3} s_Z^m$$

which ignores the fact that an X or Z chromosome that appears in a father is more likely to be in a daughter than predicted by the overall frequency of that chromosome among all daughters (e.g., an X in fathers is 100% of the time found in daughters compared to 2/3 of the Xs, and a Z in fathers is 50% of the time found in daughters compared to 1/3 of the Zs). Ditto for sex chromosomes that appear in mothers. This assumption is not necessary for Y or W chromosomes, as these spend every generation in the same sex.

YA: YA fusions originate at rate $\frac{N}{2}\mu_Y$ (we suppress the "sex" superscript, as these are always in males) in any one of the males born into the population. Eventually, the population will descend from one of these Y chromosomes. The probability that it will be the fusion chromosome is the chance that the fusion contributes to the "effective population" of males ($\frac{N_m}{N/2}$) times the fixation probability that the fusion will be the ultimate ancestor of the Y chromosomes among the descendants after some long period of time: $P_Y = \frac{1-\mathrm{Exp}[-2\, s_Y]}{1-\mathrm{Exp}[-2\, N_m\, s_Y]}$, giving an overall establishment rate of $\frac{N}{2}\mu_Y \frac{N_m}{N/2} \frac{1-\mathrm{Exp}[-2\, s_Y]}{1-\mathrm{Exp}[-2\, N_m\, s_Y]}$. When selection is negligible, this reduces to μ_Y , as in the neutral case above.

XA: XA fusions originate at rate $2\,\frac{N}{2}\mu_X^f$ in females (the "2" in front comes from the two X's in females) and $\frac{N}{2}\mu_X^m$ in males. A fusion arising in a female has a chance $\frac{N_f}{N/2}$ of surviving to reproduce and of being in the "effective population" of females; as the contribution of fathers and mothers to the next generation is equal, the initial frequency among the pool of X chromosomes in the next generation (accounting for the fact that mothers contribute two Xs and fathers one X) among reproducing adults is p = 2/3*(freq in moms) + 1/3*(freq in dads). This gives a fixation probability when appearing in females of $P_X = \frac{1-\mathrm{Exp}\left[-2\left(N_{e,X}\right)\left(\frac{2}{3}\frac{1}{2N_f}\right)s_X\right]}{1-\mathrm{Exp}\left[-2\left(N_{e,X}\right)s_X\right]}$ where $N_{e,X} = \frac{9\, N_f\, N_m}{N_f + 2\, N_m}$ is the effective population size for a X-linked locus (similarly for males); thus, the overall establishment rate of an X-A fusion is $2\,\frac{N}{2}\mu_X^f \frac{N_f}{N/2} \frac{1-\mathrm{Exp}\left[-2\left(N_{e,X}\right)\left(\frac{2}{3}\frac{1}{2N_f}\right)s_X\right]}{1-\mathrm{Exp}\left[-2\left(N_{e,X}\right)s_X\right]} + \frac{N}{2}\mu_X^m \frac{N_m}{N/2} \frac{1-\mathrm{Exp}\left[-2\left(N_{e,X}\right)\left(\frac{1}{3}\frac{1}{N_m}\right)s_X\right]}{1-\mathrm{Exp}\left[-2\left(N_{e,X}\right)s_X\right]}$. When selection is negligible, this reduces to $\frac{2}{3}\mu_X^f + \frac{1}{3}\mu_X^m$, as in the neutral case above.

WA: WA fusions originate at rate $\frac{N}{2}\mu_Y$ in any one of the females born into the population. Eventually, the population will descend from one of these W chromosomes. The probability that it will be the fusion chromosome is the chance that the fusion contributes to the "effective population" of females ($\frac{N_f}{N/2}$) times the fixation probability that the fusion will be the ultimate ancestor of the W chromosomes among the descendants after some long period of time: $P_W = \frac{1-\mathrm{Exp}[-2\, s_W]}{1-\mathrm{Exp}\left[-2\, N_f\, s_W\right]}$, giving an overall establishment rate of $\frac{N}{2}\mu_W \frac{N_f}{N/2} \frac{1-\mathrm{Exp}[-2\, s_W]}{1-\mathrm{Exp}\left[-2\, N_f\, s_W\right]}$. When selection is negligible, this reduces to μ_W , as in the neutral case above.

ZA: ZA fusions originate at rate $\frac{N}{2}\mu_X^f$ in females and $2\,\frac{N}{2}\mu_Z^m$ in males (the "2" in front comes from the two Z's in males). A fusion arising in a female has a chance $\frac{N_f}{N/2}$ of surviving to reproduce and of being in the "effective population" of females; as the contribution of fathers and mothers to the next generation is equal, the initial frequency among the pool of Z chromosomes in the next generation (accounting for the fact that mothers contribute one Z and fathers two Zs) among reproducing adults is p = 1/3*(freq in moms) + 2/3*(freq in dads). This gives a fixation probability when appearing in females of $P_Z = \frac{1-\mathrm{Exp}\left[-2\left(N_{e,Z}\right)\left(\frac{1}{3}\frac{1}{N_f}\right)s_Z\right]}{1-\mathrm{Exp}\left[-2\left(N_{e,Z}\right)s_Z\right]}$ where $N_{e,Z} = \frac{9\, N_f\, N_m}{2\, N_f + N_m}$ is the effective population size for a Z-linked locus (similarly for males); thus, the overall establishment rate of a Z-A fusion is $\frac{N}{2}\mu_Z^f \frac{N_f}{N/2} \frac{1-\mathrm{Exp}\left[-2\left(N_{e,Z}\right)\left(\frac{1}{3}\frac{1}{N_f}\right)s_Z\right]}{1-\mathrm{Exp}\left[-2\left(N_{e,Z}\right)s_Z\right]} + 2\,\frac{N}{2}\mu_Z^m \frac{N_m}{N/2} \frac{1-\mathrm{Exp}\left[-2\left(N_{e,Z}\right)\left(\frac{2}{3}\frac{1}{2N_m}\right)s_Z\right]}{1-\mathrm{Exp}\left[-2\left(N_{e,Z}\right)s_Z\right]}$. When selection is negligible, this reduces to $\frac{2}{3}\mu_Z^m + \frac{1}{3}\mu_X^f$, as in the neutral case above.

From the above, we have the following rates of establishment:

$$R_Y = \mu_Y N_m \frac{1 - \mathrm{Exp}[-2\, s_Y]}{1 - \mathrm{Exp}[-2\, N_m\, s_Y]};$$

$$R_X = 2\,\mu_X^f N_f \frac{1 - \mathrm{Exp}\left[-2\,(N_{e,X})\left(\frac{2}{3}\frac{1}{2 N_f}\right) s_X\right]}{1 - \mathrm{Exp}[-2\,(N_{e,X})\, s_X]} + \mu_X^m N_m \frac{1 - \mathrm{Exp}\left[-2\,(N_{e,X})\left(\frac{1}{3}\frac{1}{N_m}\right) s_X\right]}{1 - \mathrm{Exp}[-2\,(N_{e,X})\, s_X]};$$

$$R_W = \mu_W N_f \frac{1 - \mathrm{Exp}[-2\, s_W]}{1 - \mathrm{Exp}[-2\, N_f\, s_W]};$$

$$R_Z = \mu_Z^f N_f \frac{1 - \mathrm{Exp}\left[-2\,(N_{e,Z})\left(\frac{1}{3}\frac{1}{N_f}\right) s_Z\right]}{1 - \mathrm{Exp}[-2\,(N_{e,Z})\, s_Z]} + 2\,\mu_Z^m N_m \frac{1 - \mathrm{Exp}\left[-2\,(N_{e,Z})\left(\frac{2}{3}\frac{1}{2 N_m}\right) s_Z\right]}{1 - \mathrm{Exp}[-2\,(N_{e,Z})\, s_Z]};$$

The ratio of Y-A origination relative to X-A establishment rates is therefore

$$\mathrm{ratio}_Y = \frac{R_Y}{R_X};$$

Similarly, the rate of origination for W-A fusions relative to X-A fusions would be:

$$\mathrm{ratio}_W = \frac{R_W}{R_X};$$

and the rate of origination for Z-A fusions relative to X-A fusions would be:

$$\mathrm{ratio}_Z = \frac{R_Z}{R_X};$$

As a check, with negligible selection we regain the neutral results:

```
weaksel = {sX -> sX * small, sY -> sY * small, sZ -> sZ * small, sW -> sW * small};
Normal[Series[{ratioY, ratioW, ratioZ} /. weaksel, {small, 0, 0}]] // Simplify
```

$$\left\{\frac{3\,\mu_Y}{2\,\mu_X^f + \mu_X^m}, \frac{3\,\mu_W}{2\,\mu_X^f + \mu_X^m}, \frac{\mu_Z^f + 2\,\mu_Z^m}{2\,\mu_X^f + \mu_X^m}\right\}$$

The ratios of fusion rates, with all else equal, are given by:

```
subsimp = {Nf → N / 2, Nm → N / 2, μ^b_ _a_ → μ, μ_a_ → μ, s^y_ _x_ → s, s_x_ → s};
{ratioY, ratioW, ratioZ} /. subsimp // FullSimplify
```

$$\left\{\frac{1}{3}\,e^{-2 N s}\left(1 + e^{N s} + e^{2 N s}\right), \frac{1}{3}\,e^{-2 N s}\left(1 + e^{N s} + e^{2 N s}\right), 1\right\}$$

```
%[[1]] - %[[2]]
```

0

Assuming that the selection coefficients are the same in both sexes and that selection is very weak relative to the population sizes, the ratios of fusion rates are given by:

```
subsimp2 = {s^y_ _x_ → s, s_x_ → s};
weakratioY = Normal[
   Series[ratioY /. subsimp2 /. weaksel /. Nm -> Nm / small /. Nf -> Nf / small /. s → s * small^2, {small, 0, 1}]] /. small → 1
```

$$\frac{3\,\mu_Y}{2\,\mu_X^f + \mu_X^m} + \frac{6\, s\, N_m\,(-4\, N_f + N_m)\,\mu_Y}{(N_f + 2\, N_m)\,\left(2\,\mu_X^f + \mu_X^m\right)}$$

Or with equal reproductive sex ratio and mutation rates:

```
1 + (% - 1 /. subsimp // Simplify)
```

$1 - N s$

```
weakratioW = Normal[
   Series[ratioW /. subsimp2 /. weaksel /. Nm -> Nm / small /. Nf -> Nf / small /. s → s * small^2, {small, 0, 1}]] /. small → 1
```

$$\frac{3\,\mu_W}{2\,\mu_X^f + \mu_X^m} + \frac{3\, s\, N_f\,(N_f - 7\, N_m)\,\mu_W}{(N_f + 2\, N_m)\,\left(2\,\mu_X^f + \mu_X^m\right)}$$

Or with equal reproductive sex ratio and mutation rates:

```
1 + (% - 1 /. subsimp // Simplify)
```

$1 - N s$

```
weakratioZ = Normal[
   Series[ratioZ /. subsimp2 /. weaksel /. Nm -> Nm / small /. Nf -> Nf / small /. s → s * small^2, {small, 0, 1}]] /. small → 1
```

$$\frac{\mu_Z^f + 2\,\mu_Z^m}{2\,\mu_X^f + \mu_X^m} - \frac{9\, s\, N_f\,(N_f - N_m)\, N_m\,\left(\mu_Z^f + 2\,\mu_Z^m\right)}{(2\, N_f + N_m)\,(N_f + 2\, N_m)\,\left(2\,\mu_X^f + \mu_X^m\right)}$$

Or with equal reproductive sex ratio and mutation rates:

```
1 + (% - 1 /. subsimp // Simplify)
```

1

Thus, with both sex-biased origination rates and different numbers of reproductive males and females, the following condition must be met for Y-A fusions to be most common:

$$\frac{3\,\mu_Y}{2\,\mu_X^f+\mu_X^m} + \frac{6\,s\,N_m\,(-4\,N_f+N_m)\,\mu_Y}{(N_f+2\,N_m)\,(2\,\mu_X^f+\mu_X^m)} > \text{Max}\left[1,\ \frac{3\,\mu_W}{2\,\mu_X^f+\mu_X^m} + \frac{3\,s\,N_f\,(N_f-7\,N_m)\,\mu_W}{(N_f+2\,N_m)\,(2\,\mu_X^f+\mu_X^m)},\ \frac{\mu_Z^f+2\,\mu_Z^m}{2\,\mu_X^f+\mu_X^m} - \frac{9\,s\,N_f\,(N_f-N_m)\,N_m\,(\mu_Z^f+2\,\mu_Z^m)}{(2\,N_f+N_m)\,(N_f+2\,N_m)\,(2\,\mu_X^f+\mu_X^m)}\right]$$

Defining $\theta = 4\,(N_f + N_m)\,s$, the above can be rewritten as:

```
Solve[{Nf + Nm == Ntot, θ == 4 (Nf + Nm) s, γ == Nf / Nm}, {s, Nf, Nm}] // Simplify // Flatten
```

$$\left\{s \to \frac{\theta}{4\,N_{tot}},\ N_f \to \frac{\gamma\,N_{tot}}{1+\gamma},\ N_m \to \frac{N_{tot}}{1+\gamma}\right\}$$

```
subsimp3 = {Nf → γ Ntot/(1 + γ), Nm → Ntot/(1 + γ), μ^m_a_ → μ^m, μ^f_a_ → μ^f, μY → μ^m, μW → μ^f, s^Y_x_ → θ/(4 Ntot), s_x_ → θ/(4 Ntot), s -> θ/(4 Ntot)} /. μ^m → α μ^f;
Collect[Factor[weakratioY /. subsimp3], θ, Factor]
```

$$\frac{3\,\alpha}{2+\alpha} - \frac{3\,\alpha\,(-1+4\,\gamma)\,\theta}{2\,(2+\alpha)\,(1+\gamma)\,(2+\gamma)}$$

which can be written as $\frac{3\,\alpha}{2+\alpha}\left(1+\theta\,\frac{(1-4\,\gamma)}{2\,(1+\gamma)\,(2+\gamma)}\right)$.

```
Collect[Factor[weakratioW /. subsimp3], θ, Factor]
```

$$\frac{3}{2+\alpha} + \frac{3\,(-7+\gamma)\,\gamma\,\theta}{4\,(2+\alpha)\,(1+\gamma)\,(2+\gamma)}$$

which can be written as $\frac{3}{2+\alpha}\left(1-\theta\,\frac{\gamma\,(7-\gamma)}{4\,(1+\gamma)\,(2+\gamma)}\right)$.

```
Collect[Factor[weakratioZ /. subsimp3], θ, Factor]
```

$$\frac{1+2\,\alpha}{2+\alpha} - \frac{9\,(1+2\,\alpha)\,(-1+\gamma)\,\gamma\,\theta}{4\,(2+\alpha)\,(1+\gamma)\,(2+\gamma)\,(1+2\,\gamma)}$$

which can be written as $\frac{1+2\,\alpha}{2+\alpha}\left(1+\theta\,\frac{9\,\gamma\,(1-\gamma)}{4\,(1+\gamma)\,(2+\gamma)\,(1+2\,\gamma)}\right)$.

In the absence of sex biases in mutation rate or in sex ratio:

```
Factor[{%%%, %%, %} /. α → 1 /. γ → 1]
```

$$\left\{\frac{4-\theta}{4},\ \frac{4-\theta}{4},\ 1\right\}$$

The relative rate of X-A and Y-A fusions to W-A and Z-A fusions:

```
weakratioWZ =
 Normal[Series[(1 + ratioY)/(ratioW + ratioZ) /. subsimp2 /. weaksel /. Nm -> Nm / small /. Nf -> Nf / small /. s → s * small^2 /. subsimp3,
   {small, 0, 1}]] /. small → 1
```

$$\frac{1+2\,\alpha}{2+\alpha} - \left(3\,\left(-2\,\alpha-\alpha^2-2\,\gamma+3\,\alpha\,\gamma+8\,\alpha^2\,\gamma-8\,\gamma^2-3\,\alpha\,\gamma^2+2\,\alpha^2\,\gamma^2+\gamma^3+2\,\alpha\,\gamma^3\right)\,\theta\right) \Big/ \left(4\,(2+\alpha)^2\,(1+\gamma)\,(2+\gamma)\,(1+2\,\gamma)\right)$$

```
(1 + 2 α)/(2 + α) + θ γ/(1 + γ) (3 α/(2 + α) (1 - 4 γ)/(4 γ (2 + γ)) + 3 (1 + 2 α)/(2 + α)^2 (7 - γ)/(8 (2 + γ)) - ((1 + 2 α)/(2 + α))^2 9 (1 - γ)/(8 (2 + γ) (1 + 2 γ))) - weakratioWZ // Factor
```

0

For beneficial fusions, we can instead assume that selection is strong relative to the population size, so that (N s>>1), the fixation probabilities are approximately ($2\,s\,N_e\,p$), and we can approximate the above rates of establishment as:

```
approxratioY = (2 sY) Nm μY / (2 (2 (Ne,x) sX/(3 Nf)) Nf μ^f_X + (2 (Ne,x) sX/(3 Nm)) Nm μ^m_X);
approxratioW = (2 sW) Nf μW / (2 (2 (Ne,x) sX/(3 Nf)) Nf μ^f_X + (2 (Ne,x) sX/(3 Nm)) Nm μ^m_X);
approxratioZ = ((2 (Ne,z) sZ/(3 Nf)) Nf μ^f_Z + 2 (2 (Ne,z) sZ/(3 Nm)) Nm μ^m_Z) / (2 (2 (Ne,x) sX/(3 Nf)) Nf μ^f_X + (2 (Ne,x) sX/(3 Nm)) Nm μ^m_X);
{approxratioY, approxratioW, approxratioZ} /. s_a_ → s // Simplify
```

$$\left\{\frac{(N_f+2\,N_m)\,\mu_Y}{3\,N_f\,\left(2\,\mu_X^f+\mu_X^m\right)},\ \frac{(N_f+2\,N_m)\,\mu_W}{3\,N_m\,\left(2\,\mu_X^f+\mu_X^m\right)},\ \frac{(N_f+2\,N_m)\,\left(\mu_Z^f+2\,\mu_Z^m\right)}{(2\,N_f+N_m)\,\left(2\,\mu_X^f+\mu_X^m\right)}\right\}$$

For beneficial mutations, if the origination rate depends only on sex and not on the chromosome itself, then Y-A fusions will not be the most common if the reproductive sex ratio is equal:

```
OriginBySex = {μ^f_X → μ^f, μ^m_X → μ^m, μY → μ^m, μ^m_Z → μ^m, μ^f_Z → μ^f, μW → μ^f} /. μ^m → α μ^f;
```

```
test = %% /. OriginBySex
```

$$\left\{\frac{\alpha\,\mu^{f}\,(N_f+2\,N_m)}{3\,(2\,\mu^{f}+\alpha\,\mu^{f})\,N_f},\ \frac{\mu^{f}\,(N_f+2\,N_m)}{3\,(2\,\mu^{f}+\alpha\,\mu^{f})\,N_m},\ \frac{(\mu^{f}+2\,\alpha\,\mu^{f})\,(N_f+2\,N_m)}{(2\,\mu^{f}+\alpha\,\mu^{f})\,(2\,N_f+N_m)}\right\}$$

```
Reduce[{test[[1]] > 1, test[[1]] > test[[2]], test[[1]] > test[[3]],
   (test[[1]] + 1) > (test[[2]] + test[[3]]), N_f > 0, N_m > 0, N_f == N_m, α > 0}]
```

False

If the reproductive sex ratio is sufficiently male biased ($N_m > 4\ N_f$) and mutations happen often enough in males ($\alpha > \frac{3\,N_f}{N_m - 4\,N_f}$), however, it is possible for beneficial mutations to account for the pattern:

```
Reduce[{test[[1]] > 1, test[[1]] > test[[2]],
  test[[1]] > test[[3]], (test[[1]] + 1) > (test[[2]] + test[[3]]), N_f > 0, N_m > 0, α > 0}]
```

$$\mu^{f} \in \text{Reals}\ \&\&\ N_f > 0\ \&\&\ N_m > 4\,N_f\ \&\&\ \alpha > -\frac{3\,N_f}{4\,N_f - N_m}$$

Of course, if origination rates also depend on the chromosome itself, then if Y-A fusions originate more often than other fusions, it is possible to account for Y-A being the most commonly observed fusion, even among beneficial fusions:

```
{approxratio_Y, approxratio_W, approxratio_Z} /. s_a_ → s // Simplify;
Reduce[{%[[1]] > 1, %[[1]] > %[[2]], %[[1]] > %[[3]], N_f > 0, N_m > 0, μ_X^f > 0, μ_X^m > 0, μ_Y > 0, μ_Z^m > 0, μ_Z^f > 0, μ_W > 0}]
```

$$N_f > 0\ \&\&\ N_m > 0\ \&\&\ \mu_W > 0\ \&\&\ \mu_Y > \frac{N_f\,\mu_W}{N_m}\ \&\&\ 0 < \mu_X^{f} < \frac{N_f\,\mu_Y + 2\,N_m\,\mu_Y}{6\,N_f}\ \&\&$$
$$0 < \mu_X^{m} < \frac{-6\,N_f\,\mu_X^{f} + N_f\,\mu_Y + 2\,N_m\,\mu_Y}{3\,N_f}\ \&\&\ 0 < \mu_Z^{f} < \frac{2\,N_f\,\mu_Y + N_m\,\mu_Y}{3\,N_f}\ \&\&\ 0 < \mu_Z^{m} < \frac{2\,N_f\,\mu_Y + N_m\,\mu_Y - 3\,N_f\,\mu_Z^{f}}{6\,N_f}$$

- **Biased sex-ratios**
- **Figure 5A**

[NOTE: A slightly different version of Figure 5A was used in the final draft, which held the total population size constant, rather than holding the number of females constant. Here, we persist in using the latter to compare it to simulations that had been run under the assumption of a constant number of females.]

```
tickset = {Automatic, Join[{{Log[0.1], 0.1}},
     Table[{Log[10^i], 10^i}, {i, 0, 3}], Flatten[Table[{Log[j 10^i], ""}, {i, -1., 3}, {j, 2, 9}], 1]]};

OriginBySex = {μ_X^f → μ^f, μ_X^m → μ^m, μ_Y → μ^m, μ_Z^m → μ^m, μ_Z^f → μ^f, μ_W → μ^f} /. μ^m → α μ^f;
```

Deleterious fusions:
[Solid = Y, Dashed = W, Short-dashed = Z, all relative to X]

```
selterm = -0.0003;
plot1 = Show[
  LogPlot[ratio_Y /. N_f → N / 2 /. N_m → 1 / γ N / 2 /. N → 10 000 /. s_X → selterm /. s_Y → selterm /. OriginBySex /. α → 1,
   {γ, 1, 10}, PlotStyle → Red, PlotRange → All, AxesOrigin → {1, Log[0.05]}],
  LogPlot[ratio_W /. N_f → N / 2 /. N_m → 1 / γ N / 2 /. N → 10 000 /. s_X → selterm /. s_W → selterm /. OriginBySex /. α → 1,
   {γ, 1, 10}, PlotStyle → {Red, Dashed}, PlotRange → All],
  LogPlot[ratio_Z /. N_f → N / 2 /. N_m → 1 / γ N / 2 /. N → 10 000 /. s_X → selterm /. s_Z → selterm /. OriginBySex /. α → 1,
   {γ, 1, 10}, PlotStyle → {Red, Dotted}, PlotRange → All],
  PlotRange → {{1, 10}, {Log[0.05], Log[300]}}, Ticks -> tickset
 ]
```

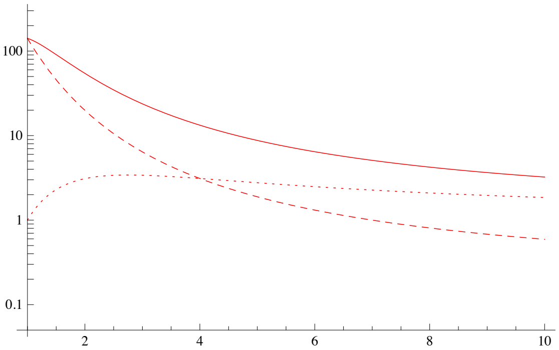

Neutral fusions:

```
selterm = 10^(-12);
plot2 = Show[
  LogPlot[ratioY /. Nf → N / 2 /. Nm → 1 / γ N / 2 /. N → 10 000 /. sX → selterm /. sY → selterm /. OriginBySex /. α → 1,
   {γ, 1, 10}, PlotStyle → Black, PlotRange → All, AxesOrigin → {1, Log[0.05]}],
  LogPlot[ratioW /. Nf → N / 2 /. Nm → 1 / γ N / 2 /. N → 10 000 /. sX → selterm /. sW → selterm /. OriginBySex /. α → 1,
   {γ, 1, 10}, PlotStyle → {Black, Dashed}, PlotRange → All],
  LogPlot[ratioZ /. Nf → N / 2 /. Nm → 1 / γ N / 2 /. N → 10 000 /. sX → selterm /. sZ → selterm /. OriginBySex /. α → 1,
   {γ, 1, 10}, PlotStyle → {Black, Dotted}, PlotRange → All],
  PlotRange → {{1, 10}, {Log[0.05], Log[300]}}, Ticks → tickset
 ]
```

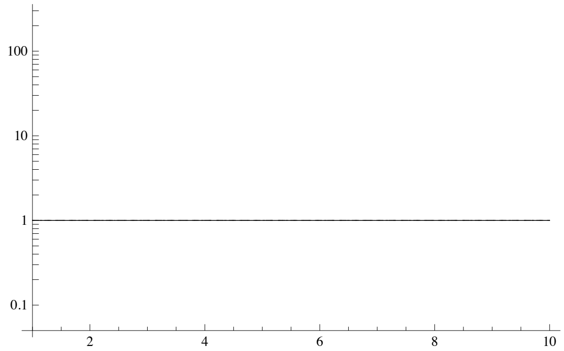

Beneficial fusions :
[Solid = Y, Dashed = W, Short-dashed = Z, all relative to X]

```
selterm = 0.0003;
plot3 = Show[
  LogPlot[ratioY /. Nf → N / 2 /. Nm → 1 / γ N / 2 /. N → 10 000 /. sX → selterm /. sY → selterm /. OriginBySex /. α → 1,
   {γ, 1, 10}, PlotStyle → Blue, PlotRange → All, AxesOrigin → {1, Log[0.05]}],
  LogPlot[ratioW /. Nf → N / 2 /. Nm → 1 / γ N / 2 /. N → 10 000 /. sX → selterm /. sW → selterm /. OriginBySex /. α → 1,
   {γ, 1, 10}, PlotStyle → {Blue, Dashed}, PlotRange → All],
  LogPlot[ratioZ /. Nf → N / 2 /. Nm → 1 / γ N / 2 /. N → 10 000 /. sX → selterm /. sZ → selterm /. OriginBySex /. α → 1,
   {γ, 1, 10}, PlotStyle → {Blue, Dotted}, PlotRange → All],
  PlotRange → {{1, 10}, {Log[0.05], Log[300]}}, Ticks → tickset
 ]
```

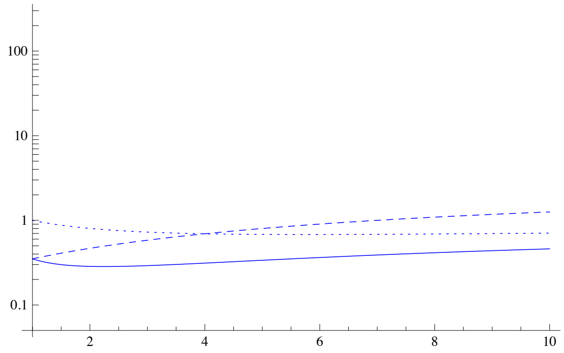

Altogether, with simulation data (see last section):
[Solid = Y, Dashed = W, Short-dashed = Z, all relative to X]

```
Show[plot1, plot2, plot3,
 ListLogPlot[{{5, 136 / (3 * 7)}, {10, 179 / (3 * 15)}}, PlotStyle → {Red, PointSize[0.02]}],
 ListLogPlot[{{5, 184 / (3 * 56)}, {10, 185 / (3 * 83)}}, PlotStyle → {Black, PointSize[0.02]}],
 ListLogPlot[{{5, 283 / (3 * 260)}, {10, 230 / (3 * 163)}}, PlotStyle → {Blue, PointSize[0.02]}],
 AxesLabel → {"Nf/Nm", "Relative establishment rate"}
]
```

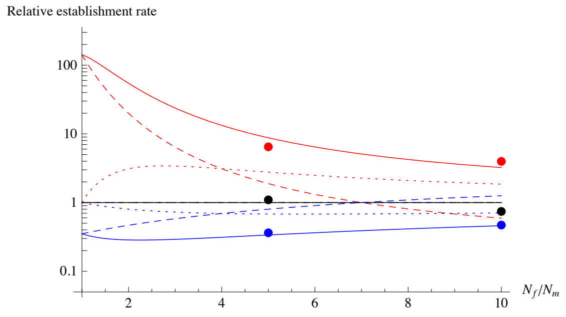

Adding 95% confidence errors based on a parametric bootstrap analysis (specifically, drawing the numerator and denominator repeatedly from a Poisson distribution given the simulated data, where the Poisson here is an excellent approximation because the number of establishments is small relative to the number of reps 10^6):

```
SeedRandom[420 573]
errorRed5 = Transpose[Join[{{5, 5},
    Quantile[Table[N[Random[PoissonDistribution[136]] / (3 * If[(ran = Random[PoissonDistribution[7]]) > 0, ran, 1])],
      {i, 1, 10 000}], {0.025, 0.975}]}]]
```

```
{{5, 3.5}, {5, 21.6667}}
```

```
errorRed10 = Transpose[Join[{{10, 10}, Quantile[
     Table[N[Random[PoissonDistribution[179]] / (3 * Random[PoissonDistribution[15]])], {i, 1, 10 000}], {0.025, 0.975}]}]]
```

```
{{10, 2.51515}, {10, 7.5}}
```

```
errorBlack5 = Transpose[Join[{{5, 5}, Quantile[
     Table[N[Random[PoissonDistribution[184]] / (3 * Random[PoissonDistribution[56]])], {i, 1, 10 000}], {0.025, 0.975}]}]]
```

```
{{5, 0.822917}, {5, 1.52137}}
```

```
errorBlack10 = Transpose[Join[{{10, 10}, Quantile[
     Table[N[Random[PoissonDistribution[185]] / (3 * Random[PoissonDistribution[83]])], {i, 1, 10 000}], {0.025, 0.975}]}]]
```

```
{{10, 0.576087}, {10, 0.975124}}
```

```
errorBlue5 = Transpose[Join[{{5, 5}, Quantile[Table[
      N[Random[PoissonDistribution[283]] / (3 * Random[PoissonDistribution[260]])], {i, 1, 10 000}], {0.025, 0.975}]}]]
```

```
{{5, 0.306931}, {5, 0.428571}}
```

```
errorBlue10 = Transpose[Join[{{10, 10}, Quantile[Table[
      N[Random[PoissonDistribution[230]] / (3 * Random[PoissonDistribution[163]])], {i, 1, 10 000}], {0.025, 0.975}]}]]
```

```
{{10, 0.38488}, {10, 0.575163}}
```

```
Show[plot1, plot2, plot3,
 ListLogPlot[{{5, 136 / (3 * 7)}, {10, 179 / (3 * 15)}}, PlotStyle → {Red, PointSize[0.02]}],
 ListLogPlot[{{5, 184 / (3 * 56)}, {10, 185 / (3 * 83)}}, PlotStyle → {Black, PointSize[0.02]}],
 ListLogPlot[{{5, 283 / (3 * 260)}, {10, 230 / (3 * 163)}}, PlotStyle → {Blue, PointSize[0.02]}],
 ListLogPlot[{errorRed5, errorRed10}, Joined → True, PlotStyle → {Red}],
 ListLogPlot[{errorBlack5, errorBlack10}, Joined → True, PlotStyle → {Black}],
 ListLogPlot[{errorBlue5, errorBlue10}, Joined → True, PlotStyle → {Blue}],
 AxesLabel → {"Nf/Nm", "Relative establishment rate"}
]
```

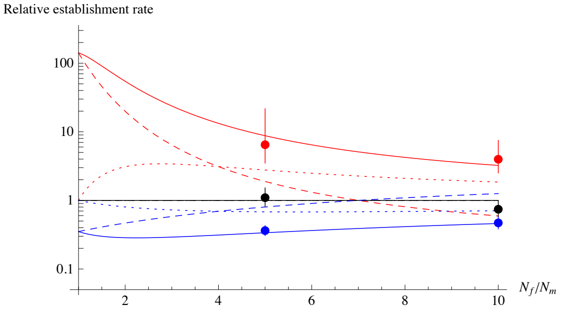

Note that the sex ratio does not matter in the neutral case (black line):

```
Normal[Series[ratioY /. Nf → N / 2 /. Nm → 1 / γ N / 2 /. N → 10 000 /. weaksel /. OriginBySex /. α → 1, {small, 0, 0}]] // Simplify
```

```
1
```

```
Normal[Series[ratioW /. Nf → N / 2 /. Nm → 1 / γ N / 2 /. N → 10 000 /. weaksel /. OriginBySex /. α → 1, {small, 0, 0}]] // Simplify
```

```
1
```

```
Normal[Series[ratioZ /. Nf → N / 2 /. Nm → 1 / γ N / 2 /. N → 10 000 /. weaksel /. OriginBySex /. α → 1, {small, 0, 0}]] // Simplify
```

```
1
```

Expectations to compare to the simulations:

[First and second sets give the expected number of fixation events out of 10^6 simulations given a single initial Y fusion and a single initial X fusion, respectively. The expected ratio of X to Y fusions is given by the third set.]

```
selterm = -0.0003;
10^6 RY / (N/2 μY) /. Nf → N / 2 /. Nm → 1 / γ N / 2 /. N → 10 000 /. sX → selterm /. sY → selterm /. OriginBySex /. α → 1 /.
  γ → {5, 10}
10^6 RX / (2 N/2 μX^f + N/2 μX^m) /. Nf → N / 2 /. Nm → 1 / γ N / 2 /. N → 10 000 /. sX → selterm /. sY → selterm /. OriginBySex /. α → 1 /.
  γ → {5, 10}
ratioY /. Nf → N / 2 /. Nm → 1 / γ N / 2 /. N → 10 000 /. sX → selterm /. sY → selterm /. OriginBySex /. α → 1 /. γ → {5, 10}
```

```
{146.008, 171.549}
```

```
{5.55192, 17.6779}
```

```
{8.76622, 3.23473}
```

```
selterm = 10^(-12.);
10^6 R_Y / (N/2 μ_Y) /. N_f → N / 2 /. N_m → 1 / γ N / 2 /. N → 10 000 /. s_X → selterm /. s_Y → selterm /. OriginBySex /. α → 1 /.
 γ → {5, 10}
10^6 R_X / (2 N/2 μ_X^f + N/2 μ_X^m) /. N_f → N / 2 /. N_m → 1 / γ N / 2 /. N → 10 000 /. s_X → selterm /. s_Y → selterm /. OriginBySex /. α → 1 /.
 γ → {5, 10}
ratio_Y /. N_f → N / 2 /. N_m → 1 / γ N / 2 /. N → 10 000 /. s_X → selterm /. s_Y → selterm /. OriginBySex /. α → 1 /. γ → {5, 10}
```

```
{199.996, 199.996}

{66.6639, 66.6706}

{1.00002, 0.999919}
```

```
selterm = 0.0003;
10^6 R_Y / (N/2 μ_Y) /. N_f → N / 2 /. N_m → 1 / γ N / 2 /. N → 10 000 /. s_X → selterm /. s_Y → selterm /. OriginBySex /. α → 1 /.
 γ → {5, 10}
10^6 R_X / (2 N/2 μ_X^f + N/2 μ_X^m) /. N_f → N / 2 /. N_m → 1 / γ N / 2 /. N → 10 000 /. s_X → selterm /. s_Y → selterm /. OriginBySex /. α → 1 /.
 γ → {5, 10}
ratio_Y /. N_f → N / 2 /. N_m → 1 / γ N / 2 /. N → 10 000 /. s_X → selterm /. s_Y → selterm /. OriginBySex /. α → 1 /. γ → {5, 10}
```

```
{265.885, 231.428}

{262.614, 167.622}

{0.337484, 0.460218}
```

- **Figure - axes in both directions (shows that N_f>N_m is needed to explain patterns)**

```
tickset =
  {Join[{{Log[0.1], 0.1}}, {{Log[1], 1}}, {{Log[10], 10}}, Flatten[Table[{Log[j 10^i], ""}, {i, -1., 3}, {j, 2, 9}], 1]],
   Join[{{Log[0.1], 0.1}}, Table[{Log[10^i], 10^i}, {i, 0, 3}],
    Flatten[Table[{Log[j 10^i], ""}, {i, -1., 3}, {j, 2, 9}], 1]]};
```

```
OriginBySex = {μ_X^f → μ^f, μ_X^m → μ^m, μ_Y → μ^m, μ_Z^m → μ^m, μ_Z^f → μ^f, μ_W → μ^f} /. μ^m → α μ^f;
```

Deleterious fusions:
[Solid = Y, Dashed = W, Short-dashed = Z, all relative to X]

```
selterm = -0.0003;
plot1 = Show[
  LogLogPlot[ratio_Y /. N_f → N / 2 /. N_m → 1 / γ N / 2 /. N → 10 000 /. s_X → selterm /. s_Y → selterm /. OriginBySex /. α → 1,
   {γ, 1 / 10, 10}, PlotStyle → Red, PlotRange → All, AxesOrigin → {Log[1], Log[0.05]}],
  LogLogPlot[ratio_W /. N_f → N / 2 /. N_m → 1 / γ N / 2 /. N → 10 000 /. s_X → selterm /. s_W → selterm /. OriginBySex /. α → 1,
   {γ, 1 / 10, 10}, PlotStyle → {Red, Dashed}, PlotRange → All],
  LogLogPlot[ratio_Z /. N_f → N / 2 /. N_m → 1 / γ N / 2 /. N → 10 000 /. s_X → selterm /. s_Z → selterm /. OriginBySex /. α → 1,
   {γ, 1 / 10, 10}, PlotStyle → {Red, Dotted}, PlotRange → All],
  PlotRange → {{Log[1 / 10], Log[10]}, {Log[0.05], Log[300]}},
  Ticks → tickset
 ]
```

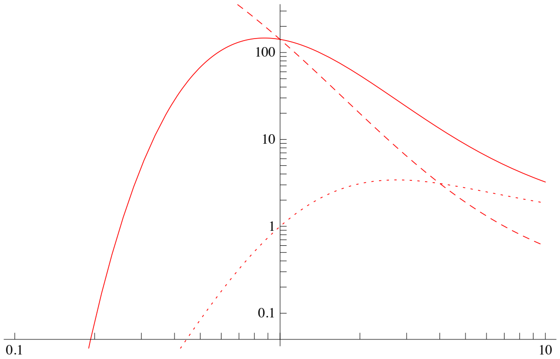

Neutral fusions:

```
selterm = 10^(-12);
plot2 = Show[
  LogLogPlot[ratioY /. Nf → N / 2 /. Nm → 1 / γ N / 2 /. N → 10 000 /. sX → selterm /. sY → selterm /. OriginBySex /. α → 1,
   {γ, 1 / 10, 10}, PlotStyle → Black, PlotRange → All, AxesOrigin → {Log[1], Log[0.05]}],
  LogLogPlot[ratioW /. Nf → N / 2 /. Nm → 1 / γ N / 2 /. N → 10 000 /. sX → selterm /. sW → selterm /. OriginBySex /. α → 1,
   {γ, 1 / 10, 10}, PlotStyle → {Black, Dashed}, PlotRange → All],
  LogLogPlot[ratioZ /. Nf → N / 2 /. Nm → 1 / γ N / 2 /. N → 10 000 /. sX → selterm /. sZ → selterm /. OriginBySex /. α → 1,
   {γ, 1 / 10, 10}, PlotStyle → {Black, Dotted}, PlotRange → All],
  PlotRange → {{Log[1 / 10], Log[10]}, {Log[0.05], Log[300]}},
  Ticks → tickset
 ]
```

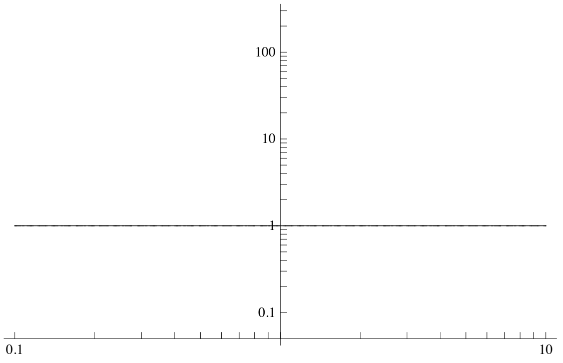

Beneficial fusions:
[Solid = Y, Dashed = W, Short-dashed = Z, all relative to X]

```
selterm = 0.0003;
plot3 = Show[
  LogLogPlot[ratioY /. Nf → N / 2 /. Nm → 1 / γ N / 2 /. N → 10 000 /. sX → selterm /. sY → selterm /. OriginBySex /. α → 1,
   {γ, 1 / 10, 10}, PlotStyle → Blue, PlotRange → All, AxesOrigin → {Log[1], Log[0.05]}],
  LogLogPlot[ratioW /. Nf → N / 2 /. Nm → 1 / γ N / 2 /. N → 10 000 /. sX → selterm /. sW → selterm /. OriginBySex /. α → 1,
   {γ, 1 / 10, 10}, PlotStyle → {Blue, Dashed}, PlotRange → All],
  LogLogPlot[ratioZ /. Nf → N / 2 /. Nm → 1 / γ N / 2 /. N → 10 000 /. sX → selterm /. sZ → selterm /. OriginBySex /. α → 1,
   {γ, 1 / 10, 10}, PlotStyle → {Blue, Dotted}, PlotRange → All],
  PlotRange → {{Log[1 / 10], Log[10]}, {Log[0.05], Log[300]}},
  Ticks → tickset
 ]
```

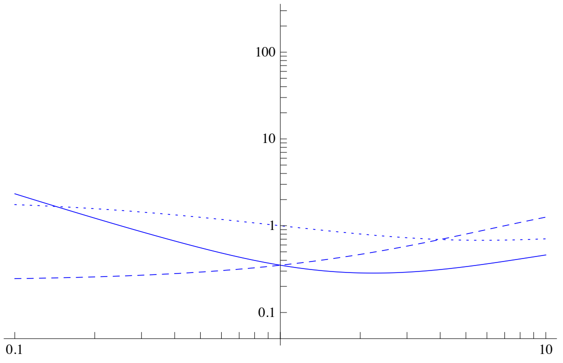

Altogether:

```
Show[plot1, plot2, plot3, AxesLabel → {"Nf/Nm", "Relative establishment rate"}]
```

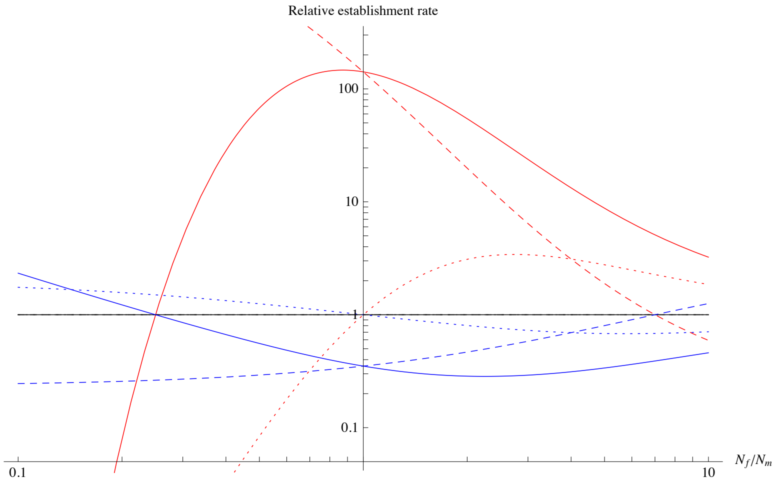

- **Figure - Net establishment rate for X-A and Y-A versus for W-A and Z-A**

Here we explore the relative ratio of XY fusions versus ZW fusions, accounting for sex ratio biases:

```
OriginBySex = {μXf → μf, μXm → μm, μY → μm, μZm → μm, μZf → μf, μW → μf} /. μm → α μf;
```

```
XYvsZW[s_, N_, γ_] =
  (ratioY + 1) / (ratioW + ratioZ) /. Nf → N / 2 /. Nm → 1 / γ N / 2 /. sX → s /. sY → s /. sW → s /. sZ → s /. OriginBySex /. α → 1 //
   Simplify;
```

As long as γ >1 and the mutations are deleterious, fusions are expected in XY lineages more often than ZW lineages:

```
selterm = -0.0003;
plot1 = Show[
  LogPlot[XYvsZW[selterm, 10 000, γ] /. OriginBySex /. α → 1, {γ, 1, 10}, PlotStyle → {Green}, PlotRange → All],
  PlotRange → {{1, 10}, {Log[0.05], Log[300]}}, Ticks -> tickset
 ]
```

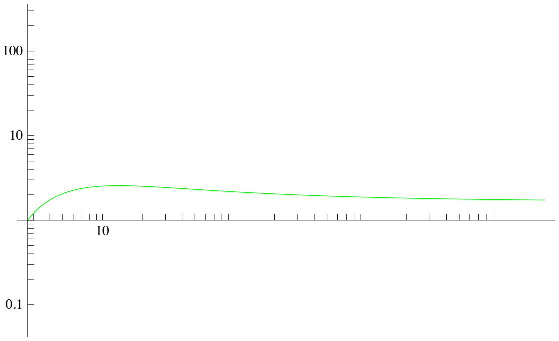

- **Sex-specific origination rates**

- **Figure 5B**

```
tickset = {Automatic, Join[{{Log[0.1], 0.1}},
    Table[{Log[10^i], 10^i}, {i, 0, 3}], Flatten[Table[{Log[j 10^i], ""}, {i, -1., 3}, {j, 2, 9}], 1]]};
```

```
OriginBySex = {μXf → μf, μXm → μm, μY → μm, μZm → μm, μZf → μf, μW → μf} /. μm → α μf;
```

Deleterious fusions:
[Solid = Y, Dashed = W, Short-dashed = Z, all relative to X]

```
selterm = -0.0003;
plot1 = Show[
  LogPlot[ratioY /. Nf → N / 2 /. Nm → N / 2 /. N → 10 000 /. sX → selterm /. sY → selterm /. OriginBySex,
   {α, 1, 10}, AxesOrigin → {1, Log[0.05]}, PlotStyle → Red],
  LogPlot[ratioW /. Nf → N / 2 /. Nm → N / 2 /. N → 10 000 /. sX → selterm /. sW → selterm /. OriginBySex,
   {α, 1, 10}, PlotStyle → {Red, Dashed}],
  LogPlot[ratioZ /. Nf → N / 2 /. Nm → N / 2 /. N → 10 000 /. sX → selterm /. sZ → selterm /. OriginBySex,
   {α, 1, 10}, PlotStyle → {Red, Dotted}],
  PlotRange → {{1, 10}, {Log[0.05], Log[300]}},
  Ticks → tickset
 ]
```

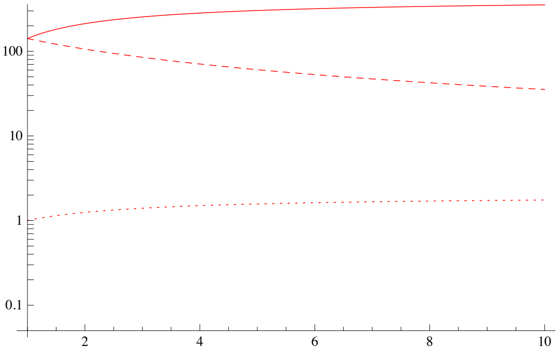

Neutral fusions:
[Solid = Y, Dashed = W, Short-dashed = Z, all relative to X]

```
selterm = 10^(-12);
plot2 = Show[
  LogPlot[ratioY /. Nf → N / 2 /. Nm → N / 2 /. N → 10 000 /. sX → selterm /. sY → selterm /. OriginBySex,
   {α, 1, 10}, AxesOrigin → {1, Log[0.05]}, PlotStyle → Black],
  LogPlot[ratioW /. Nf → N / 2 /. Nm → N / 2 /. N → 10 000 /. sX → selterm /. sW → selterm /. OriginBySex,
   {α, 1, 10}, PlotStyle → {Black, Dashed}],
  LogPlot[ratioZ /. Nf → N / 2 /. Nm → N / 2 /. N → 10 000 /. sX → selterm /. sZ → selterm /. OriginBySex,
   {α, 1, 10}, PlotStyle → {Black, Dotted}],
  PlotRange → {{1, 10}, {Log[0.05], Log[300]}},
  Ticks → tickset
 ]
```

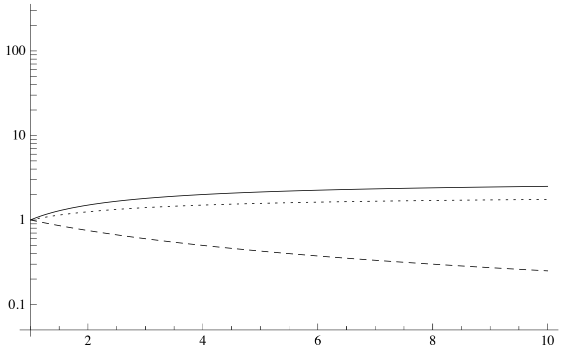

Beneficial fusions:
[Solid = Y, Dashed = W, Short-dashed = Z, all relative to X]

```
selterm = 0.0003;
plot3 = Show[
  LogPlot[ratioY /. Nf → N / 2 /. Nm → N / 2 /. N → 10 000 /. sX → selterm /. sY → selterm /. OriginBySex,
   {α, 1, 10}, AxesOrigin → {1, Log[0.05]}, PlotStyle → Blue],
  LogPlot[ratioW /. Nf → N / 2 /. Nm → N / 2 /. N → 10 000 /. sX → selterm /. sW → selterm /. OriginBySex,
   {α, 1, 10}, PlotStyle → {Blue, Dashed}],
  LogPlot[ratioZ /. Nf → N / 2 /. Nm → N / 2 /. N → 10 000 /. sX → selterm /. sZ → selterm /. OriginBySex,
   {α, 1, 10}, PlotStyle → {Blue, Dotted}],
  PlotRange → {{1, 10}, {Log[0.05], Log[300]}},
  Ticks → tickset
 ]
```

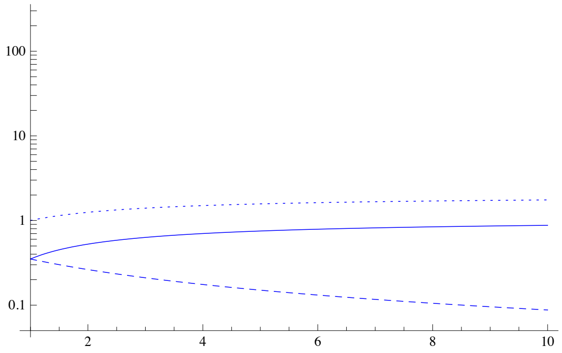

Altogether, with simulation data (see last section):
[Solid = Y, Dashed = W, Short-dashed = Z, all relative to X]

```
Show[plot1, plot2, plot3,
 ListLogPlot[{{α, 211/((2 + α)/α 62)} /. α → 5, {α, 211/((2 + α)/α * 71)} /. α → 10}, PlotStyle → {Black, PointSize[0.02]}],
 ListLogPlot[{{α, 622/((2 + α)/α * 588)} /. α → 5, {α, 622/((2 + α)/α * 579)} /. α → 10}, PlotStyle → {Blue, PointSize[0.02]}],
 AxesLabel → {"α", "Relative establishment rate"}
]
```

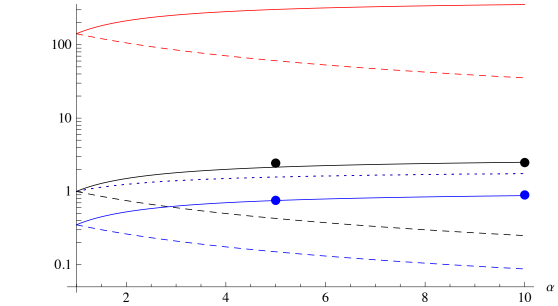

Adding 95% confidence errors based on a parametric bootstrap analysis (specifically, drawing the numerator and denominator repeatedly from a Poisson distribution given the simulated data, where the Poisson here is an excellent approximation because the number of establishments is small relative to the number of reps 10^6):

```
SeedRandom[93 460]
errorBlack5 = Transpose[
  Join[{{5, 5}, Quantile[Table[N[Random[PoissonDistribution[211]]/(((2 + α)/α /. α → 5) * Random[PoissonDistribution[62]])],
      {i, 1, 10 000}], {0.025, 0.975}]}]]
```

```
{{5, 1.85714}, {5, 3.31845}}
```

```
errorBlack10 = Transpose[Join[
   {{10, 10}, Quantile[Table[N[Random[PoissonDistribution[211]]/(((2 + α)/α /. α → 10) * Random[PoissonDistribution[71]])],
      {i, 1, 10 000}], {0.025, 0.975}]}]]
```

```
{{10, 1.9186}, {10, 3.29023}}
```

```
errorBlue5 = Transpose[
  Join[{{5, 5}, Quantile[Table[N[Random[PoissonDistribution[622]]/(((2 + α)/α /. α → 5) * Random[PoissonDistribution[588]])],
      {i, 1, 10 000}], {0.025, 0.975}]}]]
```

```
{{5, 0.67598}, {5, 0.845663}}
```

```
errorBlue10 = Transpose[Join[
   {{10, 10}, Quantile[Table[N[Random[PoissonDistribution[622]]/(((2 + α)/α /. α → 10) * Random[PoissonDistribution[579]])],
      {i, 1, 10 000}], {0.025, 0.975}]}]]
```

```
{{10, 0.799947}, {10, 1.00213}}
```

```
Show[plot1, plot2, plot3,
 ListLogPlot[{{α, 211/((2 + α)/α 62)} /. α → 5, {α, 211/((2 + α)/α * 71)} /. α → 10}, PlotStyle → {Black, PointSize[0.02]}],
 ListLogPlot[{{α, 622/((2 + α)/α * 588)} /. α → 5, {α, 622/((2 + α)/α * 579)} /. α → 10}, PlotStyle → {Blue, PointSize[0.02]}],
 ListLogPlot[{errorBlack5, errorBlack10}, Joined → True, PlotStyle → {Black}],
 ListLogPlot[{errorBlue5, errorBlue10}, Joined → True, PlotStyle → {Blue}],
 AxesLabel → {"α", "Relative establishment rate"}
]
```

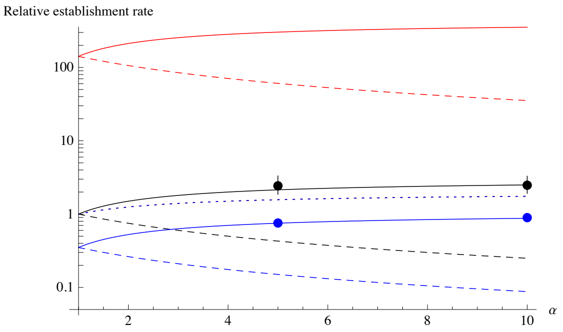

Expectations to compare to the simulations:
[First set gives the expected numbers on the Y then on the X, then the expected ratio]

```
selterm = -0.0003;
10^6 R_Y / (N/2 μ_Y) /. N_f → N / 2 /. N_m → 1 / γ N / 2 /. N → 10 000 /. s_X → selterm /. s_Y → selterm /. OriginBySex /. γ → 1 /.
 α → {5, 10}
10^6 R_X / (2 N/2 μ_X^f + N/2 μ_X^m) /. N_f → N / 2 /. N_m → 1 / γ N / 2 /. N → 10 000 /. s_X → selterm /. s_Y → selterm /. OriginBySex /. γ → 1 /.
 α → {5, 10}
ratio_Y /. N_f → N / 2 /. N_m → 1 / γ N / 2 /. N → 10 000 /. s_X → selterm /. s_Y → selterm /. OriginBySex /. γ → 1 /. α → {5, 10}
```

```
31.4469

{0.0740772, 0.0740772}

{303.225, 353.762}
```

```
selterm = 10^(-12.);
10^6 R_Y / (N/2 μ_Y) /. N_f → N / 2 /. N_m → 1 / γ N / 2 /. N → 10 000 /. s_X → selterm /. s_Y → selterm /. OriginBySex /. γ → 1 /.
 α → {5, 10}
10^6 R_X / (2 N/2 μ_X^f + N/2 μ_X^m) /. N_f → N / 2 /. N_m → 1 / γ N / 2 /. N → 10 000 /. s_X → selterm /. s_Y → selterm /. OriginBySex /. γ → 1 /.
 α → {5, 10}
ratio_Y /. N_f → N / 2 /. N_m → 1 / γ N / 2 /. N → 10 000 /. s_X → selterm /. s_Y → selterm /. OriginBySex /. γ → 1 /. α → {5, 10}
```

```
199.996

{66.6652, 66.6652}

{2.14286, 2.5}
```

```
selterm = 0.0003;
10^6 R_Y / (N/2 μ_Y) /. N_f → N / 2 /. N_m → 1 / γ N / 2 /. N → 10 000 /. s_X → selterm /. s_Y → selterm /. OriginBySex /. γ → 1 /.
 α → {5, 10}
10^6 R_X / (2 N/2 μ_X^f + N/2 μ_X^m) /. N_f → N / 2 /. N_m → 1 / γ N / 2 /. N → 10 000 /. s_X → selterm /. s_Y → selterm /. OriginBySex /. γ → 1 /.
 α → {5, 10}
ratio_Y /. N_f → N / 2 /. N_m → 1 / γ N / 2 /. N → 10 000 /. s_X → selterm /. s_Y → selterm /. OriginBySex /. γ → 1 /. α → {5, 10}
```

```
631.248

{599.894, 599.894}

{0.751618, 0.876888}
```

- **Figure - axes in both directions (shows that $\mu_m > \mu_f$ is needed to explain Y-A excess)**

```
tickset =
  {Join[{{Log[0.1], 0.1}}, {{Log[1], 1}}, {{Log[10], 10}}, Flatten[Table[{Log[j 10^i], ""}, {i, -1., 3}, {j, 2, 9}], 1]],
   Join[{{Log[0.1], 0.1}}, Table[{Log[10^i], 10^i}, {i, 0, 3}],
    Flatten[Table[{Log[j 10^i], ""}, {i, -1., 3}, {j, 2, 9}], 1]]};
```

```
OriginBySex = {μ_X^f → μ^f, μ_X^m → μ^m, μ_Y → μ^m, μ_Z^m → μ^m, μ_Z^f → μ^f, μ_W → μ^f} /. μ^m → α μ^f;
```

Deleterious fusions:
[Solid = Y, Dashed = W, Short-dashed = Z, all relative to X]

```
selterm = -0.0003;
plot1 = Show[
  LogLogPlot[ratioY /. Nf → N / 2 /. Nm → N / 2 /. N → 10 000 /. sX → selterm /. sY → selterm /. OriginBySex,
   {α, 1 / 10, 10}, AxesOrigin → {Log[1], Log[0.05]}, PlotStyle → Red],
  LogLogPlot[ratioW /. Nf → N / 2 /. Nm → N / 2 /. N → 10 000 /. sX → selterm /. sW → selterm /. OriginBySex,
   {α, 1 / 10, 10}, PlotStyle → {Red, Dashed}],
  LogLogPlot[ratioZ /. Nf → N / 2 /. Nm → N / 2 /. N → 10 000 /. sX → selterm /. sZ → selterm /. OriginBySex,
   {α, 1 / 10, 10}, PlotStyle → {Red, Dotted}],
  PlotRange → {{Log[1 / 10], Log[10]}, {Log[0.05], Log[300]}},
  Ticks → tickset
 ]
```

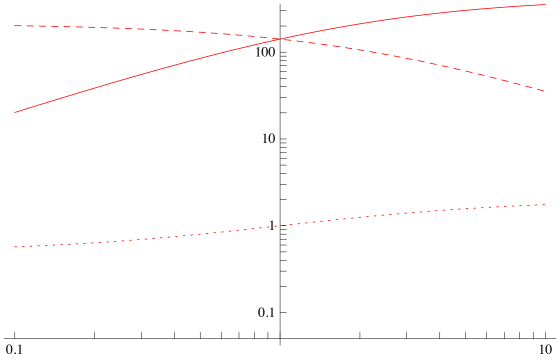

Neutral fusions:

```
selterm = 10^(-12);
plot2 = Show[
  LogLogPlot[ratioY /. Nf → N / 2 /. Nm → N / 2 /. N → 10 000 /. sX → selterm /. sY → selterm /. OriginBySex,
   {α, 1 / 10, 10}, AxesOrigin → {Log[1], Log[0.05]}, PlotStyle → Black],
  LogLogPlot[ratioW /. Nf → N / 2 /. Nm → N / 2 /. N → 10 000 /. sX → selterm /. sW → selterm /. OriginBySex,
   {α, 1 / 10, 10}, PlotStyle → {Black, Dashed}],
  LogLogPlot[ratioZ /. Nf → N / 2 /. Nm → N / 2 /. N → 10 000 /. sX → selterm /. sZ → selterm /. OriginBySex,
   {α, 1 / 10, 10}, PlotStyle → {Black, Dotted}],
  PlotRange → {{Log[1 / 10], Log[10]}, {Log[0.05], Log[300]}},
  Ticks → tickset
 ]
```

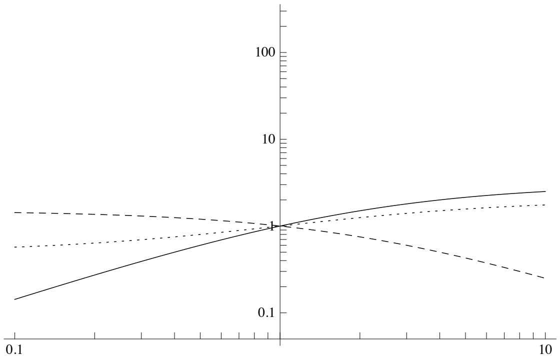

Beneficial fusions :

```
selterm = 0.0003;
plot3 = Show[
  LogLogPlot[ratioY /. Nf → N / 2 /. Nm → N / 2 /. N → 10 000 /. sX → selterm /. sY → selterm /. OriginBySex,
   {α, 1 / 10, 10}, AxesOrigin → {Log[1], Log[0.05]}, PlotStyle → Blue],
  LogLogPlot[ratioW /. Nf → N / 2 /. Nm → N / 2 /. N → 10 000 /. sX → selterm /. sW → selterm /. OriginBySex,
   {α, 1 / 10, 10}, PlotStyle → {Blue, Dashed}],
  LogLogPlot[ratioZ /. Nf → N / 2 /. Nm → N / 2 /. N → 10 000 /. sX → selterm /. sZ → selterm /. OriginBySex,
   {α, 1 / 10, 10}, PlotStyle → {Blue, Dotted}],
  PlotRange → {{Log[1 / 10], Log[10]}, {Log[0.05], Log[300]}},
  Ticks → tickset
 ]
```

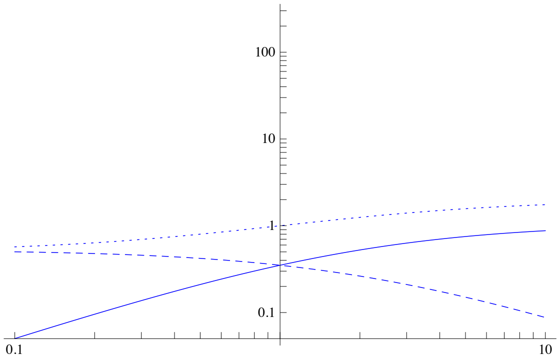

Altogether:

```
Show[plot1, plot3, plot2, AxesLabel → {"α", "Relative establishment rate"}]
```

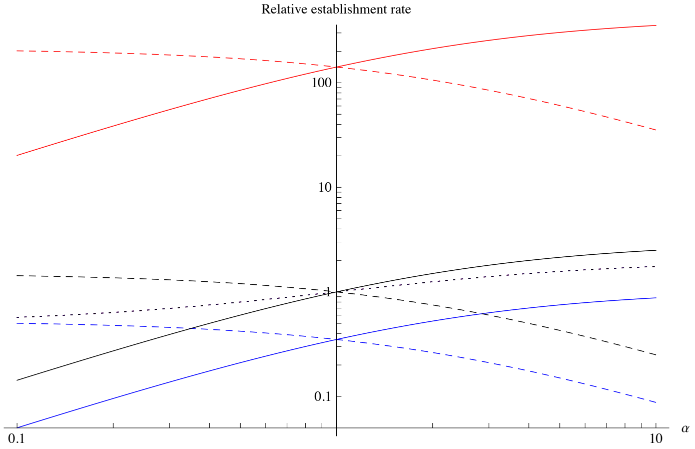

Note that the relative rate of Z-A to X-A fusions does not depend on selection differences when the sex-ratio is equal because the fixation probabilities then become equivalent:

```
ratioZ /. Nf → N / 2 /. Nm → N / 2 /. N → 10 000 /. sX -> sZ /. OriginBySex // Simplify
```

$$\frac{1+2\alpha}{2+\alpha}$$

- **Figure - Net establishment rate for X-A and Y-A versus for W-A and Z-A**

Note that the relative rate of Z-A to X-A fusions does not depend on selection differences when the sex-ratio is equal because the fixation probabilities then become equivalent:

```
ratioZ /. Nf → N / 2 /. Nm → N / 2 /. N → 10 000 /. sX -> sZ /. OriginBySex // Simplify
```

$$\frac{1+2\alpha}{2+\alpha}$$

Here we explore the relative ratio of XY fusions versus ZW fusions, accounting for sex ratio biases:

```
OriginBySex = {μXf → μf, μXm → μm, μY → μm, μZm → μm, μZf → μf, μW → μf} /. μm → α μf;
```

```
XYvsZW[s_, N_, α_] =
 (ratioY + 1 ) / (ratioW + ratioZ) /. Nf → N / 2 /. Nm → N / 2 /. sX → s /. sY → s /. sW → s /. sZ → s /. OriginBySex // Simplify
```

$$\frac{\alpha + e^{N s}\alpha + 2 e^{2 N s}(1+\alpha)}{1 + e^{N s} + 2 e^{2 N s}(1+\alpha)}$$

As long as α >1 and the mutations are deleterious, fusions are expected in XY lineages more often than ZW lineages:

```
selterm = -0.0003;
plot1 = Show[
  LogPlot[XYvsZW[selterm, 10 000, α] /. OriginBySex, {α, 1, 10}, PlotStyle → {Green}],
  PlotRange → {{1, 10}, {Log[0.05], Log[300]}},
  Ticks → tickset
 ]
```

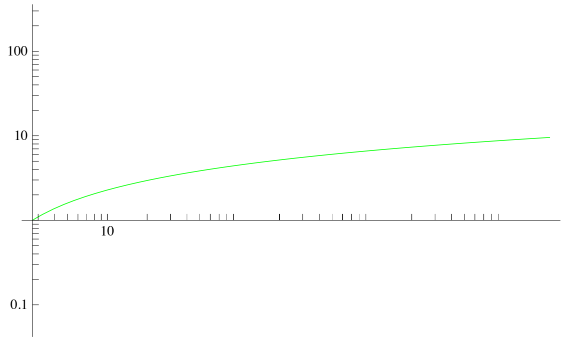

Ditto for neutral mutations:

```
selterm = 10^(-12);
plot1 = Show[
  LogPlot[XYvsZW[selterm, 10 000, α] /. OriginBySex, {α, 1, 10}, PlotStyle → {Green}],
  PlotRange → {{1, 10}, {Log[0.05], Log[300]}},
  Ticks → tickset
 ]
```

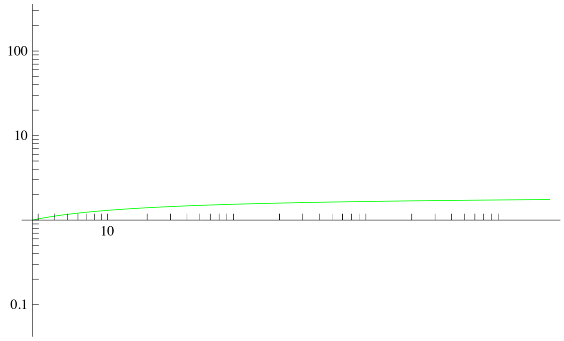

- ### Allowing both biased sex-ratios and sex-specific origination rates

```
tickset =
  {Join[Table[{i, i}, {i, -0.001, 0.001, 0.0005}], Flatten[Table[{Log[j 10^i], ""}, {i, -1., 3}, {j, 2, 9}], 1]], Join[
    {{Log[0.1], 0.1}}, Table[{Log[10^i], 10^i}, {i, 0, 3}], Flatten[Table[{Log[j 10^i], ""}, {i, -1., 3}, {j, 2, 9}], 1]]};
```

[Solid = Y, Dashed = W, Short-dashed = Z, all relative to X]

```
Manipulate[Show[
  LogPlot[
   ratio_Y /. N_f → N / 2 /. N_m → (1/ɣ) N / 2 /. N → 10000 /. s_X → s /. s_Y → s /. {μ^f_X → μ^f, μ^m_X → μ^m, μ_Y → μ^m, μ^m_Z → μ^m, μ^f_Z → μ^f, μ_W → μ^f} /.
    μ^m → α μ^f, {s, -0.001, 0.001}, AxesOrigin → {0, Log[0.05]}, PlotStyle → Black],
  LogPlot[ratio_W /. N_f → N / 2 /. N_m → (1/ɣ) N / 2 /. N → 10000 /. s_X → s /. s_W → s /.
     {μ^f_X → μ^f, μ^m_X → μ^m, μ_Y → μ^m, μ^m_Z → μ^m, μ^f_Z → μ^f, μ_W → μ^f} /. μ^m → α μ^f, {s, -0.001, 0.001}, PlotStyle → {Black, Dashed}],
  LogPlot[ratio_Z /. N_f → N / 2 /. N_m → (1/ɣ) N / 2 /. N → 10000 /. s_X → s /. s_Z → s /.
     {μ^f_X → μ^f, μ^m_X → μ^m, μ_Y → μ^m, μ^m_Z → μ^m, μ^f_Z → μ^f, μ_W → μ^f} /. μ^m → α μ^f, {s, -0.001, 0.001}, PlotStyle → {Black, Dotted}],
  PlotRange → {{-0.001, 0.001}, {Log[0.05], Log[1000]}},
  Ticks → tickset, AxesLabel → {"s", "Relative establishment rate"}], {{α, 1}, 0.1, 10}, {{ɣ, 1}, 0.1, 10}]
```

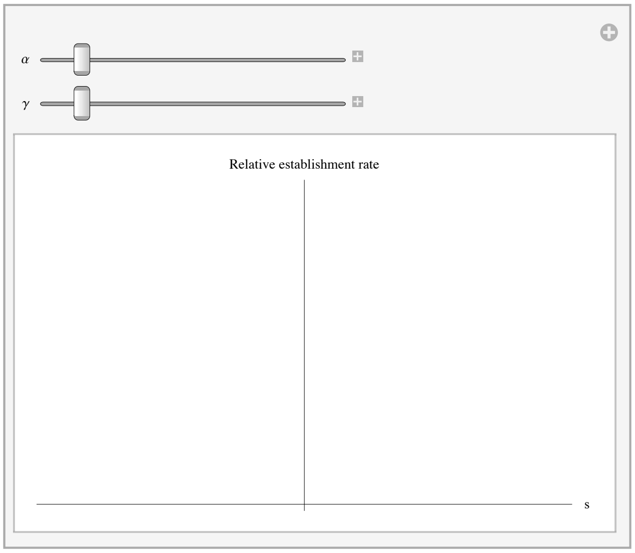

Direct fitness effects of fusions [allowing dominance and underdominance]

■ Introduction and derivation

A case of particular interest is underdominance of the fusion. To handle this case, we must include dominance within our calculation of the diffusion approximation. The following can also be used to We now modify the above to allow the fusion to have a direct fitness effect (often deleterious), which may be different in males and females. How does this affect the rate of establishment?

In the following we approximate this establishment rate using Kimura’s (1962) diffusion approximation for the fixation probability of a mutation:

$$P = \frac{\int_0^p \left(\mathrm{Exp}\left[-2\int M/V\,dx\right]\right) dx}{\int_0^1 \left(\mathrm{Exp}\left[-2\int M/V\,dx\right]\right) dx}$$

where p is the initial frequency of a mutation (here a fusion) and M and V are the mean and variance of the diffusion process and p is the initial frequency of a mutation (here a fusion). These are given by `s x (1 - x)` where s is the selection coefficient and `x (1 - x) / Ne` where N_e is the relevant effective population size of chromosomes (not of individuals). Given the number of reproducing females (N_f) and males (N_m), the effective population sizes for X and Z chromosomes are:

```
N_e,X = 9 N_f N_m / (N_f + 2 N_m);
N_e,Z = 9 N_f N_m / (2 N_f + N_m);
```

We also assume that the selection on the fusion is weak and can be taken as the average over many generations, where s_i^k is the selection coefficient for heterozygous fusions and σ_i^k for homozygous fusions. Specifically, for X and Z chromosomes, we take selection to be the long-term averages:

$$s_X = \frac{1}{3} s_X^m + \frac{2}{3} s_X^f (1 - x) + \frac{2}{3}\left(\sigma_X^f - s_X^f\right) x$$

$$s_Z = \frac{1}{3} s_Z^f + \frac{2}{3} s_Z^m (1 - x) + \frac{2}{3}\left(\sigma_X^f - s_X^f\right) x$$

which ignores the fact that an X or Z chromosome that appears in a father is more likely to be in a daughter than predicted by the overall frequency of that chromosome among all daughters (e.g., an X in fathers is 100% of the time found in daughters compared to 2/3 of the Xs, and a Z in fathers is 50% of the

time found in daughters compared to 1/3 of the Zs). Ditto for sex chromosomes that appear in mothers. This assumption is not necessary for Y or W chromosomes, as these spend every generation in the same sex.

For the hemizygous sex, we assume that the fusion is always paired with an unfused chromosome. The would suggest that s_X^m is likely equivalent in fitness to s_X^f not to σ_X^f. (This differs from the assumption in Table 2 of Charlesworth et al. 1987.)

YA: YA fusions are never homozygous, so the calculations above continue to hold, with an overall establishment rate of $\frac{N}{2}\,\mu_Y\,\frac{N_m}{N/2}\,\frac{1-\text{Exp}[-2\,s_Y]}{1-\text{Exp}[-2\,N_m\,s_Y]}$.

XA: XA fusions originate at rate $2\,\frac{N}{2}\,\mu_X^f$ in females (the “2” in front comes from the two X’s in females) and $\frac{N}{2}\,\mu_X^m$ in males. A fusion arising in a female has a chance $\frac{N_f}{N/2}$ of surviving to reproduce and of being in the “effective population” of females; as the contribution of fathers and mothers to the next generation is equal, the initial frequency among the pool of X chromosomes in the next generation (accounting for the fact that mothers contribute two Xs and fathers one X) among reproducing adults is p = 2/3*(freq in moms) + 1/3*(freq in dads). This gives a fixation probability when appearing in females of $P_X = \frac{\int_0^{\left(\frac{2}{3}\frac{1}{2N_f}\right)}\left(\text{Exp}\left[-2\int(s_X\,N_{e,X})\,dx\right]\right)dx}{\int_0^1\left(\text{Exp}\left[-2\int(s_X\,N_{e,X})\,dx\right]\right)dx}$ where $N_{e,X} = \frac{9\,N_f\,N_m}{N_f+2\,N_m}$ is the effective population size for a X-linked locus (similarly for males); thus, the overall establishment rate of an X-A fusion is $2\,\frac{N}{2}\,\mu_X^f\,\frac{N_f}{N/2}\,\frac{\int_0^{\left(\frac{2}{3}\frac{1}{2N_f}\right)}\left(\text{Exp}\left[-2\int(s_X\,N_{e,X})\,dx\right]\right)dx}{\int_0^1\left(\text{Exp}\left[-2\int(s_X\,N_{e,X})\,dx\right]\right)dx} + \frac{N}{2}\,\mu_X^m\,\frac{N_m}{N/2}\,\frac{\int_0^{\left(\frac{1}{3}\frac{1}{N_m}\right)}\left(\text{Exp}\left[-2\int(s_X\,N_{e,X})\,dx\right]\right)dx}{\int_0^1\left(\text{Exp}\left[-2\int(s_X\,N_{e,X})\,dx\right]\right)dx}$. When selection is negligible, this reduces to $\frac{2}{3}\,\mu_X^f + \frac{1}{3}\,\mu_X^m$, as in the neutral case above.

WA: WA fusions are never homozygous, so the calculations above continue to hold, with an overall establishment rate of $\frac{N}{2}\,\mu_W\,\frac{N_f}{N/2}\,\frac{1-\text{Exp}[-2\,s_W]}{1-\text{Exp}[-2\,N_f\,s_W]}$.

ZA: ZA fusions originate at rate $\frac{N}{2}\,\mu_X^f$ in females and $2\,\frac{N}{2}\,\mu_Z^m$ in males (the “2” in front comes from the two Z’s in males). A fusion arising in a female has a chance $\frac{N_f}{N/2}$ of surviving to reproduce and of being in the “effective population” of females; as the contribution of fathers and mothers to the next generation is equal, the initial frequency among the pool of Z chromosomes in the next generation (accounting for the fact that mothers contribute one Z and fathers two Zs) among reproducing adults is p = 1/3*(freq in moms) + 2/3*(freq in dads). This gives a fixation probability when appearing in females of $P_Z = \frac{\int_0^{\left(\frac{1}{3}\frac{1}{N_f}\right)}\left(\text{Exp}\left[-2\int(s_Z\,N_{e,Z})\,dx\right]\right)dx}{\int_0^1\left(\text{Exp}\left[-2\int(s_Z\,N_{e,Z})\,dx\right]\right)dx}$ where $N_{e,Z} = \frac{9\,N_f\,N_m}{2\,N_f+N_m}$ is the effective population size for a Z-linked locus (similarly for males); thus, the overall establishment rate of a Z-A fusion is $\frac{N}{2}\,\mu_Z^f\,\frac{N_f}{N/2}\,\frac{\int_0^{\left(\frac{1}{3}\frac{1}{N_f}\right)}\left(\text{Exp}\left[-2\int(s_Z\,N_{e,Z})\,dx\right]\right)dx}{\int_0^1\left(\text{Exp}\left[-2\int(s_Z\,N_{e,Z})\,dx\right]\right)dx} + 2\,\frac{N}{2}\,\mu_Z^m\,\frac{N_m}{N/2}\,\frac{\int_0^{\left(\frac{2}{3}\frac{1}{2N_m}\right)}\left(\text{Exp}\left[-2\int(s_Z\,N_{e,Z})\,dx\right]\right)dx}{\int_0^1\left(\text{Exp}\left[-2\int(s_Z\,N_{e,Z})\,dx\right]\right)dx}$.

From the above, we have the following rates of establishment:

$$\text{sX} = \frac{1}{3}\,s_X^m + \frac{2}{3}\,s_X^f\,(1-x) + \frac{2}{3}\left(\sigma_X^f - s_X^f\right)x;$$

$$\text{sZ} = \frac{1}{3}\,s_Z^f + \frac{2}{3}\,s_Z^m\,(1-x) + \frac{2}{3}\left(\sigma_Z^m - s_Z^m\right)x;$$

$$R_Y = \mu_Y\,N_m\,\frac{1-\text{Exp}[-2\,s_Y]}{1-\text{Exp}[-2\,N_m\,s_Y]};$$

$$R_W = \mu_W\,N_f\,\frac{1-\text{Exp}[-2\,s_W]}{1-\text{Exp}[-2\,N_f\,s_W]};$$

$$R_X = \mathbf{FullSimplify}\Big[$$

$$2\,\mu_X^f N_f \frac{\int_0^{\left(\frac{2}{3}\ \frac{1}{2 N_f}\right)} \left(\mathrm{Exp}\left[-2\int (sX\, N_{e,x})\, dx\right]\right) dx}{\int_0^1 \left(\mathrm{Exp}\left[-2\int (sX\, N_{e,x})\, dx\right]\right) dx} + \mu_X^m N_m \frac{\int_0^{\left(\frac{1}{3}\ \frac{1}{N_m}\right)} \left(\mathrm{Exp}\left[-2\int (sX\, N_{e,x})\, dx\right]\right) dx}{\int_0^1 \left(\mathrm{Exp}\left[-2\int (sX\, N_{e,x})\, dx\right]\right) dx} \ /.\ \sigma_X^f \to \mathrm{dif} + 2\, s_X^f\Big]\ /.\ \mathrm{dif} \to \sigma_X^f - 2\, s_X^f$$

$$\left(2\left(\mathrm{Erf}\left[\frac{\sqrt{\frac{3}{2}}\sqrt{N_f}\sqrt{N_m}\left(2 s_X^f + s_X^m\right)}{\sqrt{N_f + 2 N_m}\sqrt{-2 s_X^f + \sigma_X^f}}\right] - \mathrm{Erf}\left[\frac{\sqrt{N_m}\left(3 N_f\left(2 s_X^f + s_X^m\right) + 2\left(-2 s_X^f + \sigma_X^f\right)\right)}{\sqrt{6}\sqrt{N_f}\sqrt{N_f + 2 N_m}\sqrt{-2 s_X^f + \sigma_X^f}}\right]\right) N_f\, \mu_X^f + \left(\mathrm{Erf}\left[\frac{\sqrt{\frac{3}{2}}\sqrt{N_f}\sqrt{N_m}\left(2 s_X^f + s_X^m\right)}{\sqrt{N_f + 2 N_m}\sqrt{-2 s_X^f + \sigma_X^f}}\right] - \mathrm{Erf}\left[\frac{\sqrt{N_f}\left(3 N_m\left(2 s_X^f + s_X^m\right) + 2\left(-2 s_X^f + \sigma_X^f\right)\right)}{\sqrt{6}\sqrt{N_m}\sqrt{N_f + 2 N_m}\sqrt{-2 s_X^f + \sigma_X^f}}\right]\right) N_m\, \mu_X^m\right) \Big/ \left(\mathrm{Erf}\left[\frac{\sqrt{\frac{3}{2}}\sqrt{N_f}\sqrt{N_m}\left(2 s_X^f + s_X^m\right)}{\sqrt{N_f + 2 N_m}\sqrt{-2 s_X^f + \sigma_X^f}}\right] - \mathrm{Erf}\left[\frac{\sqrt{\frac{3}{2}}\sqrt{N_f}\sqrt{N_m}\left(s_X^m + 2\left(-s_X^f + \sigma_X^f\right)\right)}{\sqrt{N_f + 2 N_m}\sqrt{-2 s_X^f + \sigma_X^f}}\right]\right)$$

$$R_Z = \mathbf{FullSimplify}\Big[$$

$$\mu_Z^f N_f \frac{\int_0^{\left(\frac{1}{3}\ \frac{1}{N_f}\right)} \left(\mathrm{Exp}\left[-2\int (sZ\, N_{e,z})\, dx\right]\right) dx}{\int_0^1 \left(\mathrm{Exp}\left[-2\int (sZ\, N_{e,z})\, dx\right]\right) dx} + 2\,\mu_Z^m N_m \frac{\int_0^{\left(\frac{2}{3}\ \frac{1}{2 N_m}\right)} \left(\mathrm{Exp}\left[-2\int (sZ\, N_{e,z})\, dx\right]\right) dx}{\int_0^1 \left(\mathrm{Exp}\left[-2\int (sZ\, N_{e,z})\, dx\right]\right) dx} \ /.\ \sigma_Z^m \to \mathrm{dif} + 2\, s_Z^m\Big]\ /.\ \mathrm{dif} \to \sigma_Z^m - 2\, s_Z^m$$

$$\left(\left(\mathrm{Erf}\left[\frac{\sqrt{\frac{3}{2}}\sqrt{N_f}\sqrt{N_m}\left(s_Z^f + 2 s_Z^m\right)}{\sqrt{2 N_f + N_m}\sqrt{-2 s_Z^m + \sigma_Z^m}}\right] - \mathrm{Erf}\left[\frac{\sqrt{N_m}\left(3 N_f\left(s_Z^f + 2 s_Z^m\right) + 2\left(-2 s_Z^m + \sigma_Z^m\right)\right)}{\sqrt{6}\sqrt{N_f}\sqrt{2 N_f + N_m}\sqrt{-2 s_Z^m + \sigma_Z^m}}\right]\right) N_f\, \mu_Z^f + 2\left(\mathrm{Erf}\left[\frac{\sqrt{\frac{3}{2}}\sqrt{N_f}\sqrt{N_m}\left(s_Z^f + 2 s_Z^m\right)}{\sqrt{2 N_f + N_m}\sqrt{-2 s_Z^m + \sigma_Z^m}}\right] - \mathrm{Erf}\left[\frac{\sqrt{N_f}\left(3 N_m\left(s_Z^f + 2 s_Z^m\right) + 2\left(-2 s_Z^m + \sigma_Z^m\right)\right)}{\sqrt{6}\sqrt{N_m}\sqrt{2 N_f + N_m}\sqrt{-2 s_Z^m + \sigma_Z^m}}\right]\right) N_m\, \mu_Z^m\right) \Big/ \left(\mathrm{Erf}\left[\frac{\sqrt{\frac{3}{2}}\sqrt{N_f}\sqrt{N_m}\left(s_Z^f + 2 s_Z^m\right)}{\sqrt{2 N_f + N_m}\sqrt{-2 s_Z^m + \sigma_Z^m}}\right] - \mathrm{Erf}\left[\frac{\sqrt{\frac{3}{2}}\sqrt{N_f}\sqrt{N_m}\left(s_Z^f + 2 s_Z^m + 2\left(-2 s_Z^m + \sigma_Z^m\right)\right)}{\sqrt{2 N_f + N_m}\sqrt{-2 s_Z^m + \sigma_Z^m}}\right]\right)$$

For R_X, we can simplify the above assuming that the initial frequency is small (N_k is large) and selection weak, such that the numerator may be approximated by ignoring x^2 terms (as in Charlesworth et al 1987). Then, $\mathrm{Exp}\left[-2\int (sX\, N_{e,x})\, dx\right] = e^{\frac{6 N_f N_m \left(-2 x s_X^f + 2 x^2 s_X^f - x s_X^m - x^2 \sigma_X^f\right)}{N_f + 2 N_m}}$ is approximately $e^{\frac{6 N_f N_m \left(-2 x s_X^f - x s_X^m\right)}{N_f + 2 N_m}}$, and we can simplify the integration in the numerator:

$$\mathbf{Expand}\left[\left(\int_0^p \left(e^{\frac{6 N_f N_m \left(-2 x s_X^f - x s_X^m\right)}{N_f + 2 N_m}}\right) dx\right) \Big/ \frac{(N_f + 2 N_m)}{6 N_f N_m \left(2 s_X^f + s_X^m\right)}\right]$$

$$1 - e^{-\frac{6 p N_f N_m \left(2 s_X^f + s_X^m\right)}{N_f + 2 N_m}}$$

We can write the denominator as (first forcing *Mathematica* to use Erf and not DawsonF or Erfi functions):

```
Unprotect[ DawsonF, Erfi];
Format[DawsonF[x_]] := HoldForm[1/2 e^(-x^2) Sqrt[π] Erf[I x] / I];
Format[Erfi[x_]] := HoldForm[Erf[I x] / I];
Protect[DawsonF, Erfi];
```

$$\mathrm{denom} = \mathbf{FullSimplify}\left[\int_0^1 \left(\mathrm{Exp}\left[-2\int (sX\, N_{e,x})\, dx\right]\right) dx\ /.\ \sigma_X^f \to \mathrm{dif} + 2\, s_X^f,\ \left\{\left(\sqrt{-\mathrm{dif}}\right)\hat{}\,2 < 0\right\}\right]$$

$$\left(e^{\frac{3 N_f N_m \left(2 s_X^f + s_X^m\right)^2}{2\, \mathrm{dif}\, (N_f + 2 N_m)}} \sqrt{\frac{\pi}{6}} \left(-\mathrm{Erf}\left[\frac{\sqrt{\frac{3}{2}}\sqrt{N_f}\sqrt{N_m}\left(2 s_X^f + s_X^m\right)}{\sqrt{\mathrm{dif}\,(N_f + 2 N_m)}}\right] + \mathrm{Erf}\left[\frac{\sqrt{\frac{3}{2}}\sqrt{N_f}\sqrt{N_m}\left(s_X^m + 2\left(\mathrm{dif} + s_X^f\right)\right)}{\sqrt{\mathrm{dif}\,(N_f + 2 N_m)}}\right]\right) \sqrt{N_f + 2 N_m}\right) \Big/ \left(2\sqrt{\mathrm{dif}\, N_f}\sqrt{N_m}\right)$$

Giving R_X as:

$$2\,\mu_X^f N_f \frac{(N_f+2N_m)}{6N_fN_m(2s_X^f+s_X^m)}\left(1-e^{-\frac{6pN_fN_m(2s_X^f+s_X^m)}{N_f+2N_m}} \;/.\; p\to\frac{2}{3}\frac{1}{2N_f}\right)\Big/\,\text{denom}+$$

$$\mu_X^m N_m \frac{(N_f+2N_m)}{6N_fN_m(2s_X^f+s_X^m)}\left(1-e^{-\frac{6pN_fN_m(2s_X^f+s_X^m)}{N_f+2N_m}} \;/.\; p\to\frac{1}{3}\frac{1}{N_m}\right)\Big/\,\text{denom} \;/.\; \text{dif}\to\sigma_X^f-2s_X^f \;/.$$

$$\left(1-e^{-\frac{2N_{x\_}(2s_X^f+s_X^m)}{N_f+2N_m}}\right)\to\frac{2N_x}{N_f+2N_m}(2s_X^f+s_X^m)\;//\;\text{Factor}$$

$$-\left(2\,e^{-\frac{3N_fN_m(2s_X^f+s_X^m)^2}{2(N_f+2N_m)(-2s_X^f+\sigma_X^f)}}\sqrt{\frac{2}{3\pi}}\sqrt{N_m}\,(2\mu_X^f+\mu_X^m)\sqrt{-N_f(2s_X^f-\sigma_X^f)}\right)\Big/$$

$$\left(\left(\text{Erf}\left[\frac{\sqrt{\frac{3}{2}}\sqrt{N_f}\sqrt{N_m}(2s_X^f+s_X^m)}{\sqrt{(N_f+2N_m)(-2s_X^f+\sigma_X^f)}}\right]-\text{Erf}\left[\frac{\sqrt{\frac{3}{2}}\sqrt{N_f}\sqrt{N_m}(s_X^m+2(-s_X^f+\sigma_X^f))}{\sqrt{(N_f+2N_m)(-2s_X^f+\sigma_X^f)}}\right]\right)\sqrt{N_f+2N_m}\right)$$

$$\text{simpR}_X=\left(2\,e^{-\frac{3N_fN_m(2s_X^f+s_X^m)^2}{2(N_f+2N_m)(\sigma_X^f-2s_X^f)}}\sqrt{\frac{2}{3\pi}\frac{N_mN_f}{N_f+2N_m}(\sigma_X^f-2s_X^f)}\;(2\mu_X^f+\mu_X^m)\right)\Big/$$

$$\left(\text{Erf}\left[\frac{\sqrt{\frac{3}{2}}\sqrt{N_f}\sqrt{N_m}(s_X^m+2(\sigma_X^f-s_X^f))}{\sqrt{(N_f+2N_m)(\sigma_X^f-2s_X^f)}}\right]-\text{Erf}\left[\frac{\sqrt{\frac{3}{2}}\sqrt{N_f}\sqrt{N_m}(2s_X^f+s_X^m)}{\sqrt{(N_f+2N_m)(\sigma_X^f-2s_X^f)}}\right]\right);$$

where we have assumed weak selection to replace the following to leading order:

$$\text{Normal}\left[\text{Series}\left[\left(1-e^{-\frac{2N_x(2s_X^f+s_X^m)}{N_f+2N_m}}\right)\Big/(2s_X^f+s_X^m)\;/.\;s_{a\_}^{b\_}\to s_a^b*\text{small},\{\text{small},0,0\}\right]\right]$$

$$\frac{2N_x}{N_f+2N_m}$$

Similarly for R_Z, we can simplify the rate of establishment of fusions assuming large N and weak selectionabove assuming that the initial frequency is small (N_k is large) and selection weak, such that $\text{Exp}\left[-2\int(\text{sZ}\,N_{e,Z})\,dx\right]=e^{-\frac{6N_fN_m(x\,s_Z^f+2x\,s_Z^m-2x^2s_Z^m+x^2\sigma_Z^m)}{2N_f+N_m}}$ is approximately $e^{-\frac{6N_fN_m(x\,s_Z^f+2x\,s_Z^m)}{2N_f+N_m}}$ and then perform the integration in the numerator:

$$\text{Expand}\left[\left(\int_0^p\left(e^{-\frac{6N_fN_m(x\,s_Z^f+2x\,s_Z^m)}{2N_f+N_m}}\right)dx\right)\Big/\frac{(2N_f+N_m)}{6N_fN_m(s_Z^f+2s_Z^m)}\right]$$

$$1-e^{-\frac{6pN_fN_m(s_Z^f+2s_Z^m)}{2N_f+N_m}}$$

We can write the denominator as:

$$\text{denom}=\text{FullSimplify}\left[\int_0^1\left(\text{Exp}\left[-2\int(\text{sX}\,N_{e,X})\,dx\right]\right)dx\;/.\;\sigma_X^f\to\text{dif}+2s_X^f,\left\{\left(\sqrt{-\text{dif}}\right)^{\wedge}2<0\right\}\right]$$

$$\left(e^{\frac{3N_fN_m(2s_X^f+s_X^m)^2}{2\,\text{dif}\,(N_f+2N_m)}}\sqrt{\frac{\pi}{6}}\left(-\text{Erf}\left[\frac{\sqrt{\frac{3}{2}}\sqrt{N_f}\sqrt{N_m}(2s_X^f+s_X^m)}{\sqrt{\text{dif}\,(N_f+2N_m)}}\right]+\text{Erf}\left[\frac{\sqrt{\frac{3}{2}}\sqrt{N_f}\sqrt{N_m}(s_X^m+2(\text{dif}+s_X^f))}{\sqrt{\text{dif}\,(N_f+2N_m)}}\right]\right)\sqrt{N_f+2N_m}\right)\Big/\left(2\sqrt{\text{dif}\,N_f}\sqrt{N_m}\right)$$

Giving R_Z as:

$$\mu_Z^f N_f \frac{(2 N_f + N_m)}{6 N_f N_m (s_Z^f + 2 s_Z^m)} \left(1 - e^{-\frac{6 p N_f N_m (s_Z^f + 2 s_Z^m)}{2 N_f + N_m}} \text{ /. } p \text{ -> } \frac{1}{3} \frac{1}{N_f}\right) \Big/ \text{denom} +$$

$$2 \mu_Z^m N_m \frac{(2 N_f + N_m)}{6 N_f N_m (s_Z^f + 2 s_Z^m)} \left(1 - e^{-\frac{6 p N_f N_m (s_Z^f + 2 s_Z^m)}{2 N_f + N_m}} \text{ /. } p \text{ -> } \frac{2}{3} \frac{1}{2 N_m}\right) \Big/ \text{denom /. dif -> } \sigma_Z^m - 2 s_Z^m \text{ /.}$$

$$\left(1 - e^{-\frac{2 N_{x\_} (s_Z^f + 2 s_Z^m)}{2 N_f + N_m}}\right) \text{ -> } \frac{2 N_x}{2 N_f + N_m} (s_Z^f + 2 s_Z^m) \text{ // Factor}$$

$$-\left(2 e^{-\frac{3 N_f N_m (2 s_X^f + s_X^m)^2}{2 (N_f + 2 N_m)(-2 s_Z^m + \sigma_Z^m)}} \sqrt{\frac{2}{3 \pi}} \sqrt{N_m} (\mu_Z^f + 2 \mu_Z^m) \sqrt{-N_f (2 s_Z^m - \sigma_Z^m)}\right) \Big/$$

$$\left(\left(\text{Erf}\left[\frac{\sqrt{\frac{3}{2}} \sqrt{N_f} \sqrt{N_m} (2 s_X^f + s_X^m)}{\sqrt{(N_f + 2 N_m)(-2 s_Z^m + \sigma_Z^m)}}\right] - \text{Erf}\left[\frac{\sqrt{\frac{3}{2}} \sqrt{N_f} \sqrt{N_m} (s_X^m + 2 (s_X^f - 2 s_Z^m + \sigma_Z^m))}{\sqrt{(N_f + 2 N_m)(-2 s_Z^m + \sigma_Z^m)}}\right]\right) \sqrt{N_f + 2 N_m}\right)$$

$$\text{simpR}_Z = \left(2 e^{-\frac{3 N_f N_m (2 s_X^f + s_X^m)^2}{2 (N_f + 2 N_m)(-2 s_Z^m + \sigma_Z^m)}} \sqrt{\frac{2}{3 \pi} \frac{N_m N_f}{N_f + 2 N_m} (\sigma_Z^m - 2 s_Z^m)} (\mu_Z^f + 2 \mu_Z^m)\right) \Big/$$

$$\left(\text{Erf}\left[\frac{\sqrt{\frac{3}{2}} \sqrt{N_f} \sqrt{N_m} (s_X^m + 2 (s_X^f + \sigma_Z^m - 2 s_Z^m))}{\sqrt{(N_f + 2 N_m)(\sigma_Z^m - 2 s_Z^m)}}\right] - \text{Erf}\left[\frac{\sqrt{\frac{3}{2}} \sqrt{N_f} \sqrt{N_m} (2 s_X^f + s_X^m)}{\sqrt{(N_f + 2 N_m)(\sigma_Z^m - 2 s_Z^m)}}\right]\right);$$

The ratio of Y-A origination relative to X-A establishment rates is therefore

$$\text{ratio}_Y = \frac{R_Y}{R_X};$$

Similarly, the rate of origination for W-A fusions relative to X-A fusions would be:

$$\text{ratio}_W = \frac{R_W}{R_X};$$

and the rate of origination for Z-A fusions relative to X-A fusions would be:

$$\text{ratio}_Z = \frac{R_Z}{R_X};$$

As a check, with negligible selection we regain the neutral results:

$$\text{weaksel} = \{s_{x\_}^{y\_} \text{ -> } s_x^y * \text{small}, \; s_{x\_} \text{ -> } s_x * \text{small}, \; \sigma_{x\_}^{y\_} \to \sigma_x^y * \text{small}, \; \sigma_{x\_} \to \sigma_x * \text{small}\};$$

$$\text{Normal[Series[}\{\text{ratio}_Y, \text{ratio}_W, \text{ratio}_Z\} \text{ /. weaksel, \{small, 0, 0\}]] // Simplify}$$

$$\left\{\frac{3 \mu_Y}{2 \mu_X^f + \mu_X^m}, \frac{3 \mu_W}{2 \mu_X^f + \mu_X^m}, \frac{\mu_Z^f + 2 \mu_Z^m}{2 \mu_X^f + \mu_X^m}\right\}$$

The ratios of fusion rates, with all else equal, are given by:

$$\text{subsimp} = \{N_f \to N/2, \; N_m \to N/2, \; \mu_{a\_}^{b\_} \to \mu, \; \mu_{a\_} \to \mu, \; \sigma_{x\_}^{y\_} \to \sigma, \; s_{x\_}^{y\_} \to s, \; s_{x\_} \to s\};$$

$$\{\text{ratio}_Y, \text{ratio}_W, \text{ratio}_Z\} \text{ /. subsimp /. } \sigma \to \text{dif} + 2 s \text{ // FullSimplify;}$$
$$\text{\% /. dif -> } \sigma - 2 s$$

$$\left\{\left(e^{(-2+N) s} (-1 + e^{2 s}) \left(\text{Erf}\left[\frac{3 \sqrt{N} s}{2 \sqrt{-2 s + \sigma}}\right] - \text{Erf}\left[\frac{\sqrt{N} (3 s + 2 (-2 s + \sigma))}{2 \sqrt{-2 s + \sigma}}\right]\right)\right) \Big/ \left(3 (-1 + e^{N s}) \left(\text{Erf}\left[\frac{3 \sqrt{N} s}{2 \sqrt{-2 s + \sigma}}\right] - \text{Erf}\left[\frac{9 N s + 4 (-2 s + \sigma)}{6 \sqrt{N} \sqrt{-2 s + \sigma}}\right]\right)\right),\right.$$
$$\left.\left(e^{(-2+N) s} (-1 + e^{2 s}) \left(\text{Erf}\left[\frac{3 \sqrt{N} s}{2 \sqrt{-2 s + \sigma}}\right] - \text{Erf}\left[\frac{\sqrt{N} (3 s + 2 (-2 s + \sigma))}{2 \sqrt{-2 s + \sigma}}\right]\right)\right) \Big/ \left(3 (-1 + e^{N s}) \left(\text{Erf}\left[\frac{3 \sqrt{N} s}{2 \sqrt{-2 s + \sigma}}\right] - \text{Erf}\left[\frac{9 N s + 4 (-2 s + \sigma)}{6 \sqrt{N} \sqrt{-2 s + \sigma}}\right]\right)\right), 1\right\}$$

```
%[[1]] - %[[2]]
```

0

Here, "all else equal" assumes that hemizygous and heterozygous fusions have the same fitness (see next section for the different case considered by Charlesworth et al.).

Assuming that hemizygous and heterozygous fusions have the same fitness and that selection is very weak relative to the population sizes,

$$\text{subsimp2} = \{\sigma_{x\_}^{y\_} \to \sigma, \; s_{x\_}^{y\_} \to s, \; s_{x\_} \to s\};$$

$$\texttt{Normal[Series[ratio}_Y\texttt{ /. subsimp2 /. weaksel /. }N_m \texttt{->} N_m\texttt{ / small /. }N_f\texttt{ ->} N_f\texttt{ / small /. s} \to \texttt{s * small^2 /. } \sigma \to \sigma\texttt{ * small^2, \{small, 0, 1\}]] /. small} \to 1$$

$$\frac{3\,\mu_Y}{2\,\mu_X^f+\mu_X^m}+\frac{6\,N_m\,(-2\,s\,N_f-\sigma\,N_f+s\,N_m)\,\mu_Y}{(N_f+2\,N_m)\,\left(2\,\mu_X^f+\mu_X^m\right)}$$

Or with equal reproductive sex ratio and mutation rates:

$$\texttt{1 + (\% - 1 /. subsimp // Simplify)}$$

$$1-\frac{1}{3}\,N\,(s+\sigma)$$

$$\texttt{Normal[Series[ratio}_W\texttt{ /. subsimp2 /. weaksel /. }N_m \texttt{->} N_m\texttt{ / small /. }N_f\texttt{ ->} N_f\texttt{ / small /. s} \to \texttt{s * small^2 /. } \sigma \to \sigma\texttt{ * small^2, \{small, 0, 1\}]] /. small} \to 1$$

$$\frac{3\,\mu_W}{2\,\mu_X^f+\mu_X^m}+\frac{3\,N_f\,(s\,N_f-3\,s\,N_m-2\,\sigma\,N_m)\,\mu_W}{(N_f+2\,N_m)\,\left(2\,\mu_X^f+\mu_X^m\right)}$$

Or with equal reproductive sex ratio and mutation rates:

$$\texttt{1 + (\% - 1 /. subsimp // Simplify)}$$

$$1-\frac{1}{3}\,N\,(s+\sigma)$$

$$\texttt{Normal[Series[ratio}_Z\texttt{ /. subsimp2 /. weaksel /. }N_m \texttt{->} N_m\texttt{ / small /. }N_f\texttt{ ->} N_f\texttt{ / small /. s} \to \texttt{s * small^2 /. } \sigma \to \sigma\texttt{ * small^2, \{small, 0, 1\}]] /. small} \to 1$$

$$\frac{\mu_Z^f+2\,\mu_Z^m}{2\,\mu_X^f+\mu_X^m}-\frac{(5\,s+2\,\sigma)\,N_f\,(N_f-N_m)\,N_m\,\left(\mu_Z^f+2\,\mu_Z^m\right)}{(2\,N_f+N_m)\,(N_f+2\,N_m)\,\left(2\,\mu_X^f+\mu_X^m\right)}$$

Or with equal reproductive sex ratio and mutation rates:

$$\texttt{1 + (\% - 1 /. subsimp // Simplify)}$$

1

■ Comparisons against Charlesworth et al. (1987)

With weak selection, the fixation probability correctly gives the approximation given on p. 136 of Charlesworth et al. (1987):

$$\texttt{subsimpC1987 = }\left\{N_f \to N/2,\ N_m \to N/2,\ \mu_{a\_}^{b\_} \to \mu,\ \mu_{a\_} \to \mu,\ \sigma_X^f \to \sigma,\ s_X^m \to \sigma,\ s_X^f \to s\right\};$$

$$\texttt{Normal}\left[\texttt{Series}\left[\frac{\int_0^{\left(\frac{2}{3}\ \frac{1}{2N_f}\right)}\left(\texttt{Exp}\left[-2\int(sX\,N_{e,X})\,dx\right]\right)dx}{\int_0^1\left(\texttt{Exp}\left[-2\int(sX\,N_{e,X})\,dx\right]\right)dx}\texttt{ /. subsimpC1987 /. }\sigma\to\sigma\texttt{ * small /. s}\to\texttt{s * small, \{small, 0, 1\}}\right]\right]\texttt{ /. small}\to 1\texttt{ /. s}\to\texttt{h * s /. }\sigma\to\texttt{s}$$

$$\frac{2}{3\,N}+\frac{-8\,s+16\,h\,s-18\,N\,s-36\,h\,N\,s+45\,N^2\,s+18\,h\,N^2\,s}{81\,N^2}$$

For N large:

$$\texttt{Normal[Series[\% /. }\sigma\to\sigma\texttt{ * small /. s}\to\texttt{s * small /. N}\to\texttt{N / small, \{small, 0, 1\}]] /. small}\to 1$$

$$\frac{6+5\,N\,s+2\,h\,N\,s}{9\,N}$$

which is reported on p. 136.

Making the assumption of Charlesworth et al. (1987) that hemizygous fusions have the advantage of homozygous fusions, the rate of X-autosome fusions would be:

$$\texttt{R}_X\texttt{ /. subsimpC1987 /. }\sigma\to\texttt{dif + 2 s // FullSimplify;}$$
$$\texttt{\% /. dif -> }\sigma\texttt{ - 2 s}$$

$$\frac{3\,N\,\mu\left(\text{Erf}\left[\frac{\sqrt{N}\,(2\,s+\sigma)}{2\,\sqrt{-2\,s+\sigma}}\right]-\text{Erf}\left[\frac{12\,N\,s+(4+3\,N)\,(-2\,s+\sigma)}{6\,\sqrt{N}\,\sqrt{-2\,s+\sigma}}\right]\right)}{2\left(\text{Erf}\left[\frac{\sqrt{N}\,(2\,s+\sigma)}{2\,\sqrt{-2\,s+\sigma}}\right]-\text{Erf}\left[\frac{\sqrt{N}\,(4\,s+3\,(-2\,s+\sigma))}{2\,\sqrt{-2\,s+\sigma}}\right]\right)}$$

The form presented in (20) of Charlesworth et al. (1987) assumes that p is small enough (N large enough) and selection is weak, as in the derivation of $\texttt{simpR}_X$ above. Furthermore, in terms of Charlesworth's Table 2, the sign of s must be flipped and σ is αC s:

$$\texttt{simpR}_X\texttt{ /. subsimpC1987 /. s}\to\texttt{-s /. }\sigma\to\alpha\texttt{C s // Simplify}$$

$$-\frac{2\,e^{-\frac{N\,s\,(-2+\alpha C)^2}{4\,(2+\alpha C)}}\sqrt{N\,s\,(2+\alpha C)}\;\mu}{\sqrt{\pi}\left(\text{Erf}\left[\frac{N\,s\,(-2+\alpha C)}{2\,\sqrt{N\,s\,(2+\alpha C)}}\right]-\text{Erf}\left[\frac{N\,s\,(2+3\,\alpha C)}{2\,\sqrt{N\,s\,(2+\alpha C)}}\right]\right)}$$

$$\texttt{matchC = }\frac{2\,e^{-\frac{N\,s\,(2-\alpha C)^2}{4\,(2+\alpha C)}}\sqrt{N}\,\sqrt{s\,(2+\alpha C)}\;\mu}{\sqrt{\pi}\left(\text{Erf}\left[\frac{\sqrt{N}\,s\,(2+3\,\alpha C)}{2\,\sqrt{s\,(2+\alpha C)}}\right]-\text{Erf}\left[\frac{\sqrt{N}\,s\,(-2+\alpha C)}{2\,\sqrt{s\,(2+\alpha C)}}\right]\right)};$$

Compare this to Charlesworth et al. (1987) equation (20):

```
PC1987[x_] := Integrate[Exp[-1 / 2 t^2], {t, -Infinity, x}] / (2^(1/2) Pi)
KX = (μ (N s αC / Pi)^(1/2) Exp[-N s (4 - αC)^2 / (4 αC)]) /
   (PC1987[(3 αC - 4) (2 N s αC)^(1/2) / (2 αC)] - PC1987[(αC - 4) (2 N s αC)^(1/2) / (2 αC)]) // FullSimplify
```

$$\frac{2\, e^{-\frac{N s (-4+\alpha C)^2}{4 \alpha C}} \sqrt{N} \sqrt{s} \sqrt{\alpha C}\, \mu}{-\mathrm{Erf}\left[\frac{\sqrt{N} \sqrt{s} (-4+\alpha C)}{2 \sqrt{\alpha C}}\right] + \mathrm{Erf}\left[\frac{\sqrt{N} \sqrt{s} (-4+3 \alpha C)}{2 \sqrt{\alpha C}}\right]}$$

The above doesn't match our solution of the diffusion equation and suggests the following two typos (in red):

```
PC1987new[x_] := Integrate[Exp[-1 / 2 t^2], {t, -Infinity, x}] / (2 Pi)^(1/2)
KXnew = (μ (N s αC / Pi)^(1/2) Exp[-N s (4 - αC)^2 / (4 αC)]) /
   (PC1987new[(3 αC - 4) (2 N s αC)^(1/2) / (2 αC)] - PC1987new[(αC - 4) (2 N s αC)^(1/2) / (2 αC)]) /. αC → 2 + αC // FullSimplify
```

$$\frac{2\, e^{-\frac{N s (-2+\alpha C)^2}{4 (2+\alpha C)}} \sqrt{N} \sqrt{s} \sqrt{2+\alpha C}\, \mu}{\sqrt{\pi} \left(-\mathrm{Erf}\left[\frac{\sqrt{N} \sqrt{s} (-2+\alpha C)}{2 \sqrt{2+\alpha C}}\right] + \mathrm{Erf}\left[\frac{\sqrt{N} \sqrt{s} (2+3 \alpha C)}{2 \sqrt{2+\alpha C}}\right]\right)}$$

As a consequence of these typos, Equation (20) does not reach the right limit for Ns large, given by (21b):

```
Ns Limit[(KX /. N → Ns / s) / Ns, Ns → Infinity, Assumptions → αC > 4]
```

$Ns \sqrt{\pi}\, (-4 + \alpha C)\, \mu$

The corrected equation (KXnew) does, if we account for their αC actually being 2+αC:

```
Ns Limit[(KXnew /. N → Ns / s) / Ns, Ns → Infinity, Assumptions → αC > 2]
```

$Ns\, (-2 + \alpha C)\, \mu$

KXnew also reaches the correct limit for smaller αC (21a), although I think the limits are misspecified (the limit is not (2+αC) < 2 but (2+αC) < 4, i.e., αC < 2 and αC > -2/3) :

```
FullSimplify[PowerExpand[(KXnew /. N → Ns / s) / ((Ns (2 + αC) / Pi)^(1/2) Exp[-Ns (4 - (2 + αC))^2 / (4 (2 + αC))] μ)], {αC < 2}]
```

$$\frac{2}{-\mathrm{Erf}\left[\frac{\sqrt{Ns} (-2+\alpha C)}{2 \sqrt{2+\alpha C}}\right] + \mathrm{Erf}\left[\frac{\sqrt{Ns} (2+3 \alpha C)}{2 \sqrt{2+\alpha C}}\right]}$$

```
Limit[%, Ns → Infinity, Assumptions → {-2/3 < αC, αC < 2}]
```

1

```
LogPlot[2 / (-Erf[(√Ns (-2+αC)) / (2 √(2+αC))] + Erf[(√Ns (2+3 αC)) / (2 √(2+αC))]) /. Ns → 10^5, {αC, -2, 4}, PlotRange → All, AxesLabel → {"αC", "Should be one"}]
```

General::unfl : Underflow occurred in computation. ≫

General::unfl : Underflow occurred in computation. ≫

Power::infy : Infinite expression $\frac{1}{0.}$ encountered. ≫

Power::infy : Infinite expression $\frac{1}{0.}$ encountered. ≫

Power::infy : Infinite expression $\frac{1}{0.}$ encountered. ≫

General::stop : Further output of Power::infy will be suppressed during this calculation. ≫

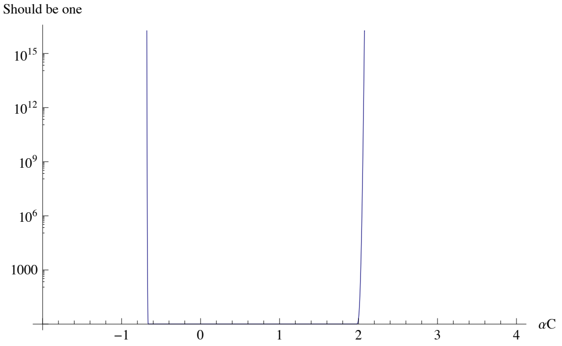

- **Biased sex-ratios**
- **Exploring Figure 5A with dominance and underdominance**

```
tickset = {Automatic, Join[{{Log[0.1], 0.1}},
    Table[{Log[10^i], 10^i}, {i, 0, 6}], Flatten[Table[{Log[j 10^i], ""}, {i, -1., 6}, {j, 2, 9}], 1]]};
```

OriginBySex = $\{\mu_X^f \to \mu^f,\ \mu_X^m \to \mu^m,\ \mu_Y \to \mu^m,\ \mu_Z^m \to \mu^m,\ \mu_Z^f \to \mu^f,\ \mu_W \to \mu^f\}$ **/.** $\mu^m \to \alpha\,\mu^f$**;**

subsimp = $\{\sigma_{X\_}^{Y\_} \to \sigma,\ s_{X\_}^{Y\_} \to s,\ s_{X\_} \to s\}$**;**

We get the same results as for in the previous section where we assumed an additive allele.

E.g., for deleterious fusions:
[Solid = Y, Dashed = W, Short-dashed = Z, all relative to X]
[There’s a division by zero problem if $\sigma \to 2s$, requiring a separate limit be taken, we avoid this here by subtracting a small amount off σ.]

```
selterm = -0.0003;
sigterm = 2 selterm - 10^(-6);
plot1 = Show[
  LogPlot[
   Chop[ratioY /. subsimp /. Nf → N / 2 /. Nm → 1 / γ N / 2 /. N → 10 000 /. s → selterm /. σ → sigterm /. OriginBySex /. α → 1],
   {γ, 1 / 10, 10}, PlotStyle → Red, PlotRange → All, AxesOrigin → {1, Log[0.05]}],
  LogPlot[Chop[ratioW /. subsimp /. Nf → N / 2 /. Nm → 1 / γ N / 2 /. N → 10 000 /. s → selterm /. σ → sigterm /. OriginBySex /.
     α → 1], {γ, 1 / 10, 10}, PlotStyle → {Red, Dashed}, PlotRange → All], LogPlot[
   Chop[ratioZ /. subsimp /. Nf → N / 2 /. Nm → 1 / γ N / 2 /. N → 10 000 /. s → selterm /. σ → sigterm /. OriginBySex /. α → 1],
   {γ, 1 / 10, 10}, PlotStyle → {Red, Dotted}, PlotRange → All],
  PlotRange → {{1 / 10, 10}, {Log[0.05], Log[300]}}, Ticks -> tickset
 ]
```

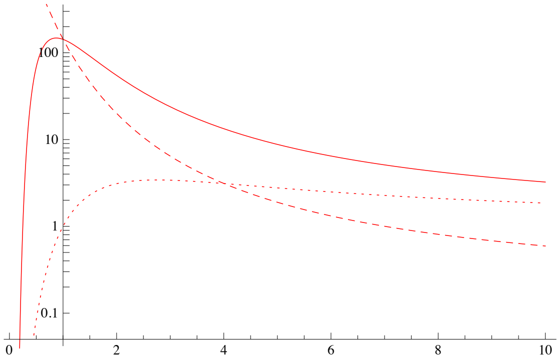

The results are not fundamentally altered for deleterious mutations (or beneficial mutations) if we vary the dominance coefficient, here with h = 4/5 (although the y-axis shifts):

```
selterm = -0.0003;
sigterm = 1.25 selterm;
plot1 = Show[
  LogPlot[
   Chop[ratioY /. subsimp /. Nf → N / 2 /. Nm → 1 / γ N / 2 /. N → 10 000 /. s → selterm /. σ → sigterm /. OriginBySex /. α → 1],
   {γ, 1 / 10, 10}, PlotStyle → Red, PlotRange → All, AxesOrigin → {1, Log[0.05]}],
  LogPlot[Chop[ratioW /. subsimp /. Nf → N / 2 /. Nm → 1 / γ N / 2 /. N → 10 000 /. s → selterm /. σ → sigterm /. OriginBySex /.
     α → 1], {γ, 1 / 10, 10}, PlotStyle → {Red, Dashed}, PlotRange → All], LogPlot[
   Chop[ratioZ /. subsimp /. Nf → N / 2 /. Nm → 1 / γ N / 2 /. N → 10 000 /. s → selterm /. σ → sigterm /. OriginBySex /. α → 1],
   {γ, 1 / 10, 10}, PlotStyle → {Red, Dotted}, PlotRange → All],
  PlotRange → {{1 / 10, 10}, {Log[0.05], Log[1000]}}, Ticks -> tickset
 ]
```

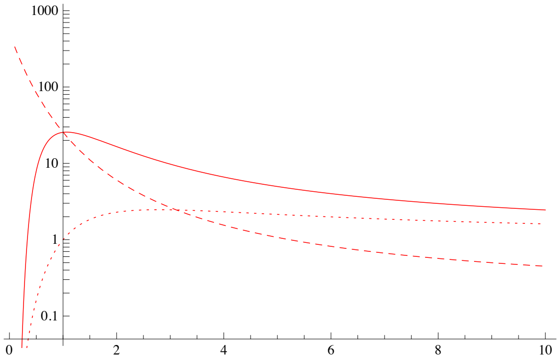

Here with h = 1/5 (although the y-axis shifts):

```
selterm = -0.0003;
sigterm = 5 selterm;
plot1 = Show[
  LogPlot[
   Chop[ratioY /. subsimp /. Nf → N / 2 /. Nm → 1 / γ N / 2 /. N → 10 000 /. s → selterm /. σ → sigterm /. OriginBySex /. α → 1],
   {γ, 1 / 10, 10}, PlotStyle → Red, PlotRange → All, AxesOrigin → {1, Log[0.05]}],
  LogPlot[Chop[ratioW /. subsimp /. Nf → N / 2 /. Nm → 1 / γ N / 2 /. N → 10 000 /. s → selterm /. σ → sigterm /. OriginBySex /.
      α → 1], {γ, 1 / 10, 10}, PlotStyle → {Red, Dashed}, PlotRange → All], LogPlot[
   Chop[ratioZ /. subsimp /. Nf → N / 2 /. Nm → 1 / γ N / 2 /. N → 10 000 /. s → selterm /. σ → sigterm /. OriginBySex /. α → 1],
   {γ, 1 / 10, 10}, PlotStyle → {Red, Dotted}, PlotRange → All],
  PlotRange → {{1, 10}, {Log[0.05], Log[5 000 000]}}, Ticks -> tickset
 ]
```

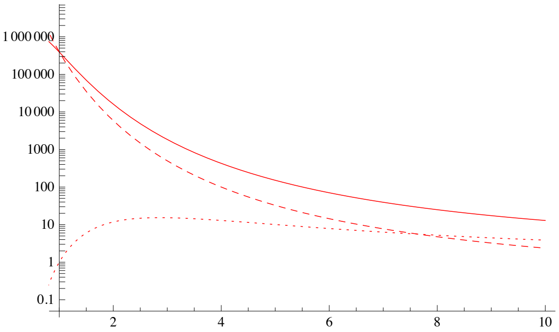

Underdominant mutations:
[Solid = Y, Dashed = W, Short-dashed = Z, all relative to X]

Homozygotes equal in fitness:

```
selterm = -0.0003;
sigterm = 10^-8;
plot1 = Show[
  LogPlot[
   Chop[ratioY /. subsimp /. Nf → N / 2 /. Nm → 1 / γ N / 2 /. N → 10 000 /. s → selterm /. σ → sigterm /. OriginBySex /. α → 1],
   {γ, 1 / 10, 10}, PlotStyle → Green, PlotRange → All, AxesOrigin → {1, Log[0.05]}],
  LogPlot[Chop[ratioW /. subsimp /. Nf → N / 2 /. Nm → 1 / γ N / 2 /. N → 10 000 /. s → selterm /. σ → sigterm /. OriginBySex /.
      α → 1], {γ, 1 / 10, 10}, PlotStyle → {Green, Dashed}, PlotRange → All], LogPlot[
   Chop[ratioZ /. subsimp /. Nf → N / 2 /. Nm → 1 / γ N / 2 /. N → 10 000 /. s → selterm /. σ → sigterm /. OriginBySex /. α → 1],
   {γ, 1 / 10, 10}, PlotStyle → {Green, Dotted}, PlotRange → All],
  PlotRange → {{1 / 10, 10}, {Log[0.05], Log[1000]}}, Ticks -> tickset
 ]
```

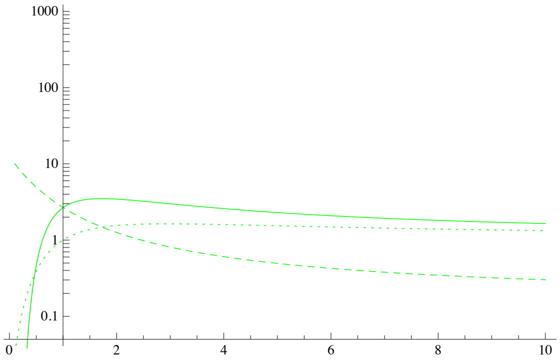

Fusion homozygotes more fit:

```
selterm = -0.0003;
sigterm = 0.0003;
plot1 = Show[
  LogPlot[
   Chop[ratioY /. subsimp /. Nf → N / 2 /. Nm → 1 / γ N / 2 /. N → 10000 /. s → selterm /. σ → sigterm /. OriginBySex /. α → 1],
   {γ, 1 / 10, 10}, PlotStyle → Green, PlotRange → All, AxesOrigin → {1, Log[0.05]}],
  LogPlot[Chop[ratioW /. subsimp /. Nf → N / 2 /. Nm → 1 / γ N / 2 /. N → 10000 /. s → selterm /. σ → sigterm /. OriginBySex /.
      α → 1], {γ, 1 / 10, 10}, PlotStyle → {Green, Dashed}, PlotRange → All], LogPlot[
   Chop[ratioZ /. subsimp /. Nf → N / 2 /. Nm → 1 / γ N / 2 /. N → 10000 /. s → selterm /. σ → sigterm /. OriginBySex /. α → 1],
   {γ, 1 / 10, 10}, PlotStyle → {Green, Dotted}, PlotRange → All],
  PlotRange → {{1 / 10, 10}, {Log[0.05], Log[1000]}}, Ticks -> tickset
 ]
```

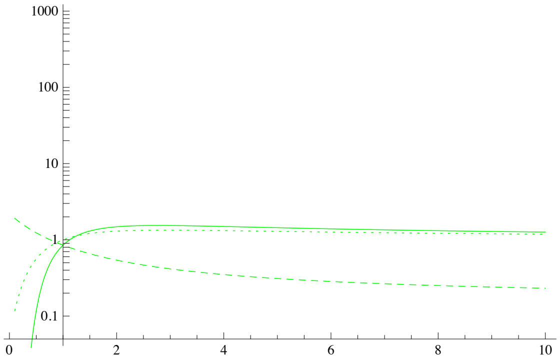

Fusion homozygotes even more fit:

```
selterm = -0.0003;
sigterm = 0.0006;
plot1 = Show[
  LogPlot[
   Chop[ratioY /. subsimp /. Nf → N / 2 /. Nm → 1 / γ N / 2 /. N → 10000 /. s → selterm /. σ → sigterm /. OriginBySex /. α → 1],
   {γ, 1 / 10, 10}, PlotStyle → Green, PlotRange → All, AxesOrigin → {1, Log[0.05]}],
  LogPlot[Chop[ratioW /. subsimp /. Nf → N / 2 /. Nm → 1 / γ N / 2 /. N → 10000 /. s → selterm /. σ → sigterm /. OriginBySex /.
      α → 1], {γ, 1 / 10, 10}, PlotStyle → {Green, Dashed}, PlotRange → All], LogPlot[
   Chop[ratioZ /. subsimp /. Nf → N / 2 /. Nm → 1 / γ N / 2 /. N → 10000 /. s → selterm /. σ → sigterm /. OriginBySex /. α → 1],
   {γ, 1 / 10, 10}, PlotStyle → {Green, Dotted}, PlotRange → All],
  PlotRange → {{1 / 10, 10}, {Log[0.05], Log[300]}}, Ticks -> tickset
 ]
```

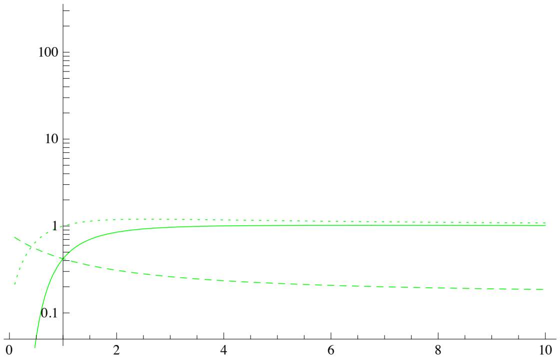

The above graphs indicate that underdominant fusions are less likely to account for an excess of Y-fusions than deleterious fusions (solid curves higher), unless fusion homozygotes aren’t that fit. The reason is that underdominance causes X-fusions and Z-fusions to fix more easily because of their homozygous advantage, relative to Y-fusions and W-fusions, which remain heterozygous.

Even making the assumption of Charlesworth et al. (1987) that fusions in the hemizygous sex gain the benefit of homozygous fusions does not lead to an excess of Y fusions:
[Solid = Y, Dashed = W, Short-dashed = Z, all relative to X]

$$\text{subsimp} = \{\sigma^f_X \to \sigma,\ \sigma^m_Z \to \sigma,\ s^m_X \to \sigma,\ s^f_Z \to \sigma,\ s_Y \to \sigma,\ s_W \to \sigma,\ s^f_X \to s,\ s^m_Z \to s\};$$

```
selterm = -0.0003;
sigterm = 10^-8;
plot1 = Show[
  LogPlot[
   Chop[ratioY /. subsimp /. Nf → N / 2 /. Nm → 1 / γ N / 2 /. N → 10 000 /. s → selterm /. σ → sigterm /. OriginBySex /. α → 1],
   {γ, 1 / 10, 10}, PlotStyle → Green, PlotRange → All, AxesOrigin → {1, Log[0.05]}],
  LogPlot[Chop[ratioW /. subsimp /. Nf → N / 2 /. Nm → 1 / γ N / 2 /. N → 10 000 /. s → selterm /. σ → sigterm /. OriginBySex /.
     α → 1], {γ, 1 / 10, 10}, PlotStyle → {Green, Dashed, Thick}, PlotRange → All], LogPlot[
   Chop[ratioZ /. subsimp /. Nf → N / 2 /. Nm → 1 / γ N / 2 /. N → 10 000 /. s → selterm /. σ → sigterm /. OriginBySex /. α → 1],
   {γ, 1 / 10, 10}, PlotStyle → {Green, Dotted}, PlotRange → All],
  PlotRange → {{1 / 10, 10}, {Log[0.05], Log[300]}}, Ticks -> tickset
 ]
```

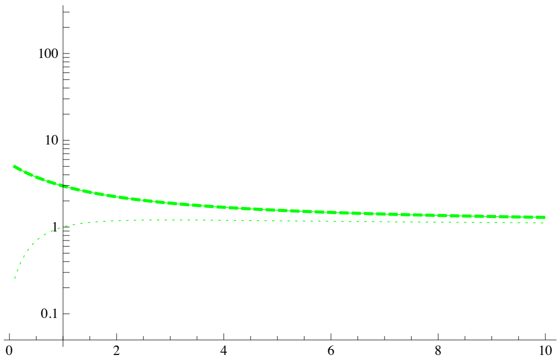

(ratio$_Y$ and ratio$_W$ lie on top of each other.)

```
selterm = -0.0003;
sigterm = 0.0003;
plot1 = Show[
  LogPlot[
   Chop[ratioY /. subsimp /. Nf → N / 2 /. Nm → 1 / γ N / 2 /. N → 10 000 /. s → selterm /. σ → sigterm /. OriginBySex /. α → 1],
   {γ, 1 / 10, 10}, PlotStyle → Green, PlotRange → All, AxesOrigin → {1, Log[0.05]}],
  LogPlot[Chop[ratioW /. subsimp /. Nf → N / 2 /. Nm → 1 / γ N / 2 /. N → 10 000 /. s → selterm /. σ → sigterm /. OriginBySex /.
     α → 1], {γ, 1 / 10, 10}, PlotStyle → {Green, Dashed}, PlotRange → All], LogPlot[
   Chop[ratioZ /. subsimp /. Nf → N / 2 /. Nm → 1 / γ N / 2 /. N → 10 000 /. s → selterm /. σ → sigterm /. OriginBySex /. α → 1],
   {γ, 1 / 10, 10}, PlotStyle → {Green, Dotted}, PlotRange → All],
  PlotRange → {{1 / 10, 10}, {Log[0.05], Log[300]}}, Ticks -> tickset
 ]
```

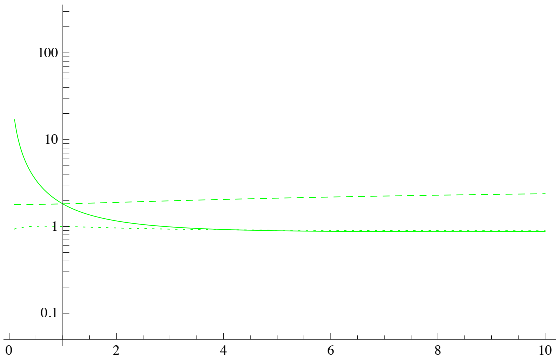

```
selterm = -0.0003;
sigterm = 0.0006;
plot1 = Show[
  LogPlot[
   Chop[ratioY /. subsimp /. Nf → N / 2 /. Nm → 1 / γ N / 2 /. N → 10000 /. s → selterm /. σ → sigterm /. OriginBySex /. α → 1],
   {γ, 1 / 10, 10}, PlotStyle → Green, PlotRange → All, AxesOrigin → {1, Log[0.05]}],
  LogPlot[Chop[ratioW /. subsimp /. Nf → N / 2 /. Nm → 1 / γ N / 2 /. N → 10000 /. s → selterm /. σ → sigterm /. OriginBySex /.
     α → 1], {γ, 1 / 10, 10}, PlotStyle → {Green, Dashed}, PlotRange → All], LogPlot[
   Chop[ratioZ /. subsimp /. Nf → N / 2 /. Nm → 1 / γ N / 2 /. N → 10000 /. s → selterm /. σ → sigterm /. OriginBySex /. α → 1],
   {γ, 1 / 10, 10}, PlotStyle → {Green, Dotted}, PlotRange → All],
  PlotRange → {{1 / 10, 10}, {Log[0.05], Log[300]}}, Ticks -> tickset
 ]
```

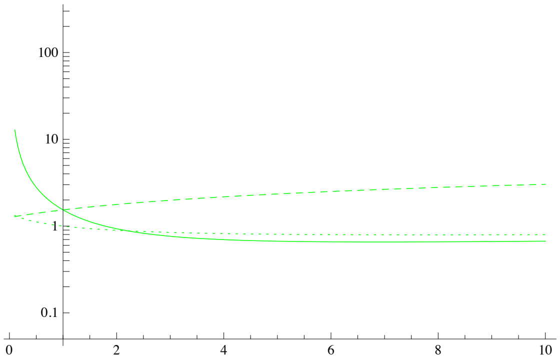

Under the Charlesworth assumptions, Y-fusions and W-fusions would then be beneficial, in which case W-fusions become more common if females have the larger number of reproductive individuals.

- **Sex-specific origination rates**
- **Exploring Figure 5B with dominance and underdominance**

```
tickset = {Automatic, Join[{{Log[0.1], 0.1}},
    Table[{Log[10^i], 10^i}, {i, 0, 5}], Flatten[Table[{Log[j 10^i], ""}, {i, -1., 5}, {j, 2, 9}], 1]]};
```

OriginBySex = $\{\mu_X^f \to \mu^f,\ \mu_X^m \to \mu^m,\ \mu_Y \to \mu^m,\ \mu_Z^m \to \mu^m,\ \mu_Z^f \to \mu^f,\ \mu_W \to \mu^f\}$ /. $\mu^m \to \alpha\,\mu^f$;

subsimp = $\{\sigma_{x\_}^{Y\_} \to \sigma,\ s_{x\_}^{Y\_} \to s,\ s_{x\_} \to s\}$;

We get the same results as for in the previous section where we assumed an additive allele.

E.g., for deleterious fusions:
[Solid = Y, Dashed = W, Short-dashed = Z, all relative to X]
[There's a division by zero problem if $\sigma \to 2s$, requiring a separate limit be taken, we avoid this here by subtracting a small amount off σ.]

```
selterm = -0.0003;
sigterm = 2 selterm - 10^(-6);
plot1 = Show[
  LogPlot[Chop[ratioY /. subsimp /. Nf → N / 2 /. Nm → N / 2 /. N → 10000 /. s → selterm /. σ → sigterm /. OriginBySex],
   {α, 1 / 10, 10}, AxesOrigin → {1, Log[0.05]}, PlotStyle → Red],
  LogPlot[Chop[ratioW /. subsimp /. Nf → N / 2 /. Nm → N / 2 /. N → 10000 /. s → selterm /. σ → sigterm /. OriginBySex],
   {α, 1 / 10, 10}, PlotStyle → {Red, Dashed}],
  LogPlot[Chop[ratioZ /. subsimp /. Nf → N / 2 /. Nm → N / 2 /. N → 10000 /. s → selterm /. σ → sigterm /. OriginBySex],
   {α, 1 / 10, 10}, PlotStyle → {Red, Dotted}],
  PlotRange → {{1 / 10, 10}, {Log[0.05], Log[300]}},
  Ticks → tickset
 ]
```

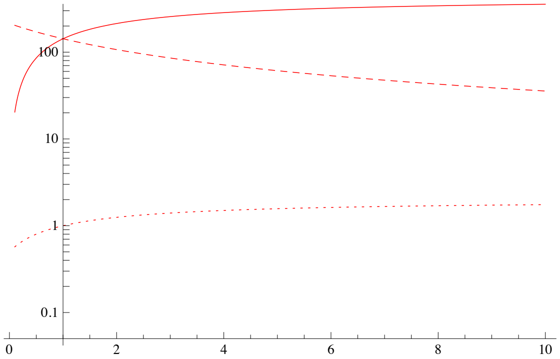

The results are not fundamentally altered for deleterious mutations (or beneficial mutations) if we vary the dominance coefficient slightly, here with h = 4/5 [the y-axis changes, but the relative positions of the curves does not]:

```
selterm = -0.0003;
sigterm = 1.25 selterm;
plot1 = Show[
  LogPlot[Chop[ratioY /. subsimp /. Nf → N / 2 /. Nm → N / 2 /. N → 10 000 /. s → selterm /. σ → sigterm /. OriginBySex],
   {α, 1 / 10, 10}, AxesOrigin → {1, Log[0.05]}, PlotStyle → Red],
  LogPlot[Chop[ratioW /. subsimp /. Nf → N / 2 /. Nm → N / 2 /. N → 10 000 /. s → selterm /. σ → sigterm /. OriginBySex],
   {α, 1 / 10, 10}, PlotStyle → {Red, Dashed}],
  LogPlot[Chop[ratioZ /. subsimp /. Nf → N / 2 /. Nm → N / 2 /. N → 10 000 /. s → selterm /. σ → sigterm /. OriginBySex],
   {α, 1 / 10, 10}, PlotStyle → {Red, Dotted}],
  PlotRange → {{1 / 10, 10}, {Log[0.05], Log[300]}},
  Ticks → tickset
 ]
```

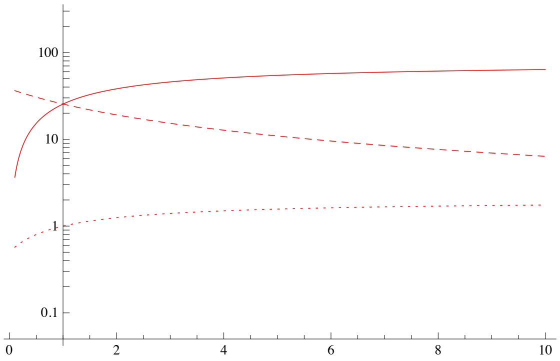


```
selterm = -0.0003;
sigterm = 5 selterm;
plot1 = Show[
  LogPlot[Chop[ratioY /. subsimp /. Nf → N / 2 /. Nm → N / 2 /. N → 10 000 /. s → selterm /. σ → sigterm /. OriginBySex],
   {α, 1 / 10, 10}, AxesOrigin → {1, Log[0.05]}, PlotStyle → Red],
  LogPlot[Chop[ratioW /. subsimp /. Nf → N / 2 /. Nm → N / 2 /. N → 10 000 /. s → selterm /. σ → sigterm /. OriginBySex],
   {α, 1 / 10, 10}, PlotStyle → {Red, Dashed}],
  LogPlot[Chop[ratioZ /. subsimp /. Nf → N / 2 /. Nm → N / 2 /. N → 10 000 /. s → selterm /. σ → sigterm /. OriginBySex],
   {α, 1 / 10, 10}, PlotStyle → {Red, Dotted}],
   PlotRange → {{1 / 10, 10}, {Log[0.05], Log[1 000 000]}}],
  Ticks → tickset
 ]
```

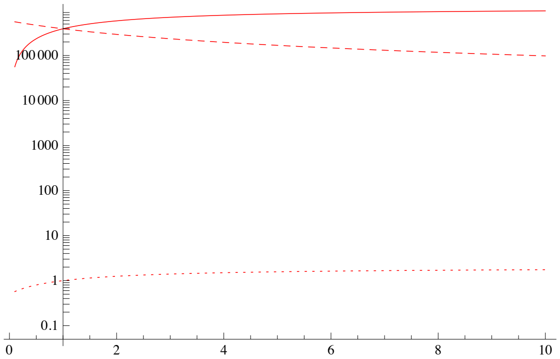

Underdominant mutations:

[Solid = Y, Dashed = W, Short-dashed = Z, all relative to X]

Homozygotes equal in fitness:

```
selterm = -0.0003;
sigterm = 0;
plot1 = Show[
  LogPlot[Chop[ratioY /. subsimp /. Nf → N / 2 /. Nm → N / 2 /. N → 10 000 /. s → selterm /. σ → sigterm /. OriginBySex],
   {α, 1 / 10, 10}, AxesOrigin → {1, Log[0.05]}, PlotStyle → Green],
  LogPlot[Chop[ratioW /. subsimp /. Nf → N / 2 /. Nm → N / 2 /. N → 10 000 /. s → selterm /. σ → sigterm /. OriginBySex],
   {α, 1 / 10, 10}, PlotStyle → {Green, Dashed}],
  LogPlot[Chop[ratioZ /. subsimp /. Nf → N / 2 /. Nm → N / 2 /. N → 10 000 /. s → selterm /. σ → sigterm /. OriginBySex],
   {α, 1 / 10, 10}, PlotStyle → {Green, Dotted}],
  PlotRange → {{1 / 10, 10}, {Log[0.05], Log[5000]}},
  Ticks → tickset
 ]
```

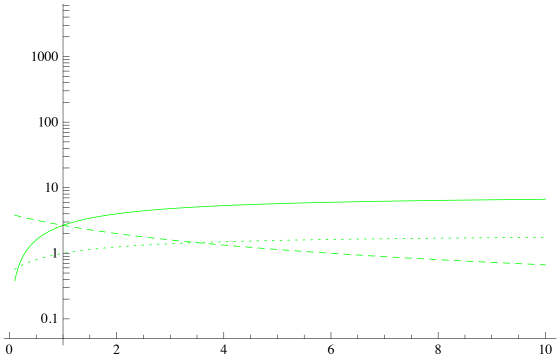

Fusion homozygotes more fit:

```
selterm = -0.0003;
sigterm = 0.0003;
plot1 = Show[
  LogPlot[Chop[ratioY /. subsimp /. Nf → N / 2 /. Nm → N / 2 /. N → 10 000 /. s → selterm /. σ → sigterm /. OriginBySex],
   {α, 1 / 10, 10}, AxesOrigin → {1, Log[0.05]}, PlotStyle → Green],
  LogPlot[Chop[ratioW /. subsimp /. Nf → N / 2 /. Nm → N / 2 /. N → 10 000 /. s → selterm /. σ → sigterm /. OriginBySex],
   {α, 1 / 10, 10}, PlotStyle → {Green, Dashed}],
  LogPlot[Chop[ratioZ /. subsimp /. Nf → N / 2 /. Nm → N / 2 /. N → 10 000 /. s → selterm /. σ → sigterm /. OriginBySex],
   {α, 1 / 10, 10}, PlotStyle → {Green, Dotted}],
  PlotRange → {{1 / 10, 10}, {Log[0.05], Log[5000]}},
  Ticks → tickset
 ]
```

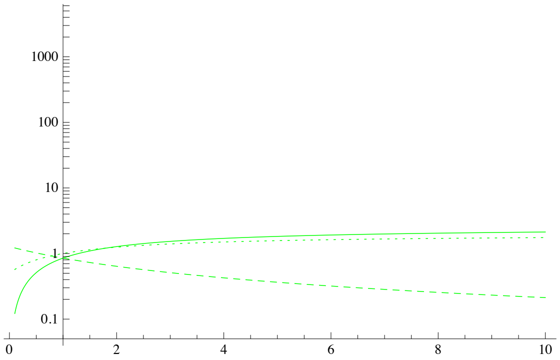

Fusion homozygotes even more fit:

```
selterm = -0.0003;
sigterm = 0.0006;
plot1 = Show[
  LogPlot[Chop[ratioY /. subsimp /. Nf → N / 2 /. Nm → N / 2 /. N → 10 000 /. s → selterm /. σ → sigterm /. OriginBySex],
   {α, 1 / 10, 10}, AxesOrigin → {1, Log[0.05]}, PlotStyle → Green],
  LogPlot[Chop[ratioW /. subsimp /. Nf → N / 2 /. Nm → N / 2 /. N → 10 000 /. s → selterm /. σ → sigterm /. OriginBySex],
   {α, 1 / 10, 10}, PlotStyle → {Green, Dashed}],
  LogPlot[Chop[ratioZ /. subsimp /. Nf → N / 2 /. Nm → N / 2 /. N → 10 000 /. s → selterm /. σ → sigterm /. OriginBySex],
   {α, 1 / 10, 10}, PlotStyle → {Green, Dotted}],
  PlotRange → {{1 / 10, 10}, {Log[0.05], Log[5000]}},
  Ticks → tickset
 ]
```

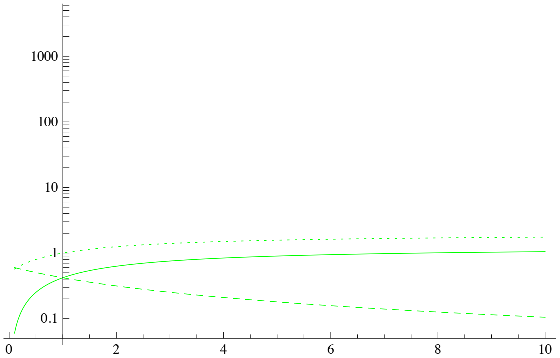

The above graphs indicate that underdominant fusions are less likely to account for an excess of Y-fusions than deleterious fusions (solid curves higher), unless fusion homozygotes aren't that fit. The reason is that underdominance causes X-fusions and Z-fusions to fix more easily because of their homozygous advantage, relative to Y-fusions and W-fusions, which remain heterozygous.

Even making the assumption of Charlesworth et al. (1987) that fusions in the hemizygous sex gain the benefit of homozygous fusions does not help explain an excess of Y fusions:
[Solid = Y, Dashed = W, Short-dashed = Z, all relative to X]

```
subsimp = {σX^f → σ, σZ^m → σ, sX^m → σ, sZ^f → σ, sY → σ, sW → σ, sX^f → s, sZ^m → s};
```

```
selterm = -0.0003;
sigterm = 10^-8;
plot1 = Show[
  LogPlot[Chop[ratioY /. subsimp /. Nf → N / 2 /. Nm → N / 2 /. N → 10 000 /. s → selterm /. σ → sigterm /. OriginBySex],
   {α, 1 / 10, 10}, AxesOrigin → {1, Log[0.05]}, PlotStyle → Green],
  LogPlot[Chop[ratioW /. subsimp /. Nf → N / 2 /. Nm → N / 2 /. N → 10 000 /. s → selterm /. σ → sigterm /. OriginBySex],
   {α, 1 / 10, 10}, PlotStyle → {Green, Dashed}],
  LogPlot[Chop[ratioZ /. subsimp /. Nf → N / 2 /. Nm → N / 2 /. N → 10 000 /. s → selterm /. σ → sigterm /. OriginBySex],
   {α, 1 / 10, 10}, PlotStyle → {Green, Dotted}],
  PlotRange → {{1 / 10, 10}, {Log[0.05], Log[5000]}},
  Ticks → tickset
 ]
```

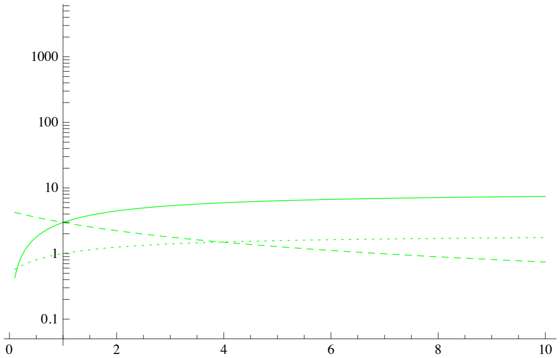

Note that the solid (Y/X) and dashed (W/X) curves have both dropped relative to the short-dashed curve (Z/X), which becomes more exagerated with an increased fitness advantage to fusion homozygotes:

```
selterm = -0.0003;
sigterm = 0.0003;
plot1 = Show[
  LogPlot[Chop[ratioY /. subsimp /. Nf → N / 2 /. Nm → N / 2 /. N → 10 000 /. s → selterm /. σ → sigterm /. OriginBySex],
   {α, 1 / 10, 10}, AxesOrigin → {1, Log[0.05]}, PlotStyle → Green],
  LogPlot[Chop[ratioW /. subsimp /. Nf → N / 2 /. Nm → N / 2 /. N → 10 000 /. s → selterm /. σ → sigterm /. OriginBySex],
   {α, 1 / 10, 10}, PlotStyle → {Green, Dashed}],
  LogPlot[Chop[ratioZ /. subsimp /. Nf → N / 2 /. Nm → N / 2 /. N → 10 000 /. s → selterm /. σ → sigterm /. OriginBySex],
   {α, 1 / 10, 10}, PlotStyle → {Green, Dotted}],
  PlotRange → {{1 / 10, 10}, {Log[0.05], Log[5000]}},
  Ticks → tickset
 ]
```

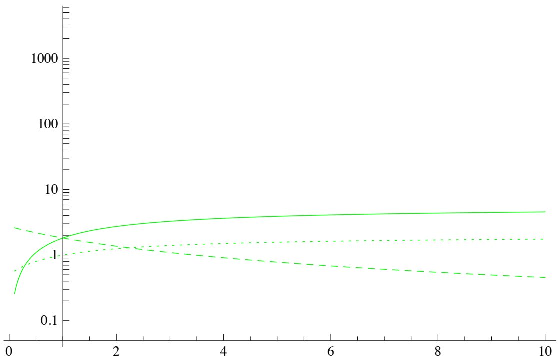


```
selterm = -0.0003;
sigterm = 0.0006;
plot1 = Show[
  LogPlot[Chop[ratioY /. subsimp /. Nf → N / 2 /. Nm → N / 2 /. N → 10 000 /. s → selterm /. σ → sigterm /. OriginBySex],
   {α, 1 / 10, 10}, AxesOrigin → {1, Log[0.05]}, PlotStyle → Green],
  LogPlot[Chop[ratioW /. subsimp /. Nf → N / 2 /. Nm → N / 2 /. N → 10 000 /. s → selterm /. σ → sigterm /. OriginBySex],
   {α, 1 / 10, 10}, PlotStyle → {Green, Dashed}],
  LogPlot[Chop[ratioZ /. subsimp /. Nf → N / 2 /. Nm → N / 2 /. N → 10 000 /. s → selterm /. σ → sigterm /. OriginBySex],
   {α, 1 / 10, 10}, PlotStyle → {Green, Dotted}],
  PlotRange → {{1 / 10, 10}, {Log[0.05], Log[5000]}},
  Ticks → tickset
 ]
```

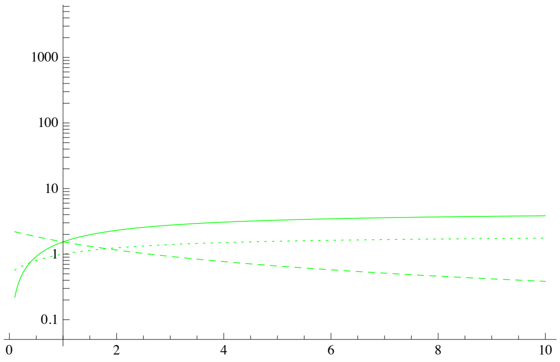

Under the Charlesworth assumptions (with αC>0), Y-fusions and W-fusions are beneficial, in which case Y-fusions are more common if the male mutation rate is higher, but so too is the rate of Z-fusions, which occur more often in males and become relatively more frequent as the homozygous fusion becomes more fit.

The biggest impact of underdominance is to increase the absolute rate of fusions, although this won't help Y-fusions or W-fusions unless we assume hemizygote fusions to have the same fitness advantage as homozygous fusions.

Meiotic drive favoring fusions

In this section, we assume that meiotic drive occurs only in females, which generates sex-specific selection. Meiotic drive in males can be treated simply be interchanging the definitions of the sexes (males <-> females, Z<->X, and W<->Y). Meiotic drive acting equivalently in both sexes would be equivalent to non-sex-specific selection, as in the previous graphs (treating the extent of drive "f" as the selection coefficient "s").

Introduction and derivation

Here we explore meiotic drive, where fusion heterozygotes produce a fraction $\frac{1}{2}(1+f)$ of gametes with fusions and $\frac{1}{2}(1-f)$ without. This leads to a change in allele frequency that is equivalent to the selection in Kimura's model (assuming weak selection, with heterozygous effect s and homozygous effect ~2s):

```
(p^2 + p q (1 + f)) - p /. q → 1 - p // Factor
```

```
-f (-1 + p) p
```

```
Normal[Series[(p^2 + p q (1 + s)) / (p^2 + 2 p q (1 + s) + q^2 (1 + s)^2) - p /. q → 1 - p, {s, 0, 1}]] // Factor
```

```
(-1 + p) p s
```

When meiotic drive occurs in only one sex (here, females), we must account for the fraction of time each chromosomes spends in that sex.

Assuming that meiotic drive is weak, we can again take an average of the resulting selection on the fusion over many generations:

$$s_X = \frac{2}{3} f$$
$$s_Y = 0$$
$$s_Z = \frac{1}{3} f$$
$$s_W = f$$

These values can be used in the expressions above to get the establishment probability. For the additive case:

$$N_{e,X} = \frac{9 N_f N_m}{N_f + 2 N_m};$$

$$N_{e,Z} = \frac{9 N_f N_m}{2 N_f + N_m};$$

$$R_Y = \mu_Y N_m \frac{1 - Exp[-2 s_Y]}{1 - Exp[-2 N_m s_Y]};$$

$$R_X = 2 \mu_X^f N_f \frac{1 - Exp\left[-2 (N_{e,X}) \left(\frac{2}{3} \frac{1}{2 N_f}\right) s_X\right]}{1 - Exp[-2 (N_{e,X}) s_X]} + \mu_X^m N_m \frac{1 - Exp\left[-2 (N_{e,X}) \left(\frac{1}{3} \frac{1}{N_m}\right) s_X\right]}{1 - Exp[-2 (N_{e,X}) s_X]};$$

$$R_W = \mu_W N_f \frac{1 - Exp[-2 s_W]}{1 - Exp[-2 N_f s_W]};$$

$$R_Z = \mu_Z^f N_f \frac{1 - Exp\left[-2 (N_{e,Z}) \left(\frac{1}{3} \frac{1}{N_f}\right) s_Z\right]}{1 - Exp[-2 (N_{e,Z}) s_Z]} + 2 \mu_Z^m N_m \frac{1 - Exp\left[-2 (N_{e,Z}) \left(\frac{2}{3} \frac{1}{2 N_m}\right) s_Z\right]}{1 - Exp[-2 (N_{e,Z}) s_Z]};$$

$$ratio_Y = \frac{R_Y}{R_X};$$

$$ratio_W = \frac{R_W}{R_X};$$

$$ratio_Z = \frac{R_Z}{R_X};$$

- **Biased sex-ratios**
- **Figure (meiotic drive against fusions, f < 0, is needed to explain Y-A excess)**

```
tickset = {Automatic, Join[{{Log[0.1], 0.1}},
    Table[{Log[10^i], 10^i}, {i, 0, 3}], Flatten[Table[{Log[j 10^i], ""}, {i, -1., 3}, {j, 2, 9}], 1]]};
```

$OriginBySex = \{\mu_X^f \to \mu^f, \mu_X^m \to \mu^m, \mu_Y \to \mu^m, \mu_Z^m \to \mu^m, \mu_Z^f \to \mu^f, \mu_W \to \mu^f\}$ /. $\mu^m \to \alpha \mu^f$;

Deleterious fusions:
[Solid = Y, Dashed = W, Short-dashed = Z, all relative to X]

```
selterm = -0.0003;
subsel = {sX → 2 / 3 selterm, sY → 10^(-12), sW → selterm, sZ → 1 / 3 selterm};
plot1 = Show[
  LogPlot[ratioY /. Nf → N / 2 /. Nm → 1 / γ N / 2 /. N → 10 000 /. subsel /. OriginBySex /. α → 1,
   {γ, 1, 10}, PlotStyle → Red, PlotRange → All, AxesOrigin → {1, Log[0.05]}],
  LogPlot[ratioW /. Nf → N / 2 /. Nm → 1 / γ N / 2 /. N → 10 000 /. subsel /. OriginBySex /. α → 1,
   {γ, 1, 10}, PlotStyle → {Red, Dashed}, PlotRange → All],
  LogPlot[ratioZ /. Nf → N / 2 /. Nm → 1 / γ N / 2 /. N → 10 000 /. subsel /. OriginBySex /. α → 1,
   {γ, 1, 10}, PlotStyle → {Red, Dotted}, PlotRange → All],
  PlotRange → {{1, 10}, {Log[0.05], Log[300]}}, Ticks -> tickset
 ]
```

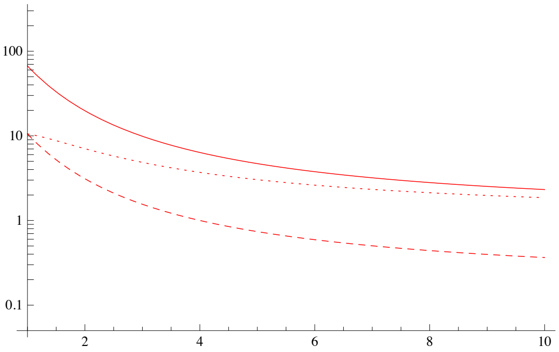

Neutral fusions:

```
selterm = 10^(-12);
subsel = {sX → 2 / 3 selterm, sY → 10^(-12), sW → selterm, sZ → 1 / 3 selterm};
plot2 = Show[
  LogPlot[ratioY /. Nf → N / 2 /. Nm → 1 / γ N / 2 /. N → 10 000 /. subsel /. OriginBySex /. α → 1,
   {γ, 1, 10}, PlotStyle → Black, PlotRange → All, AxesOrigin → {1, Log[0.05]}],
  LogPlot[ratioW /. Nf → N / 2 /. Nm → 1 / γ N / 2 /. N → 10 000 /. subsel /. OriginBySex /. α → 1,
   {γ, 1, 10}, PlotStyle → {Black, Dashed}, PlotRange → All],
  LogPlot[ratioZ /. Nf → N / 2 /. Nm → 1 / γ N / 2 /. N → 10 000 /. subsel /. OriginBySex /. α → 1,
   {γ, 1, 10}, PlotStyle → {Black, Dotted}, PlotRange → All],
  PlotRange → {{1, 10}, {Log[0.05], Log[300]}}, Ticks → tickset
 ]
```

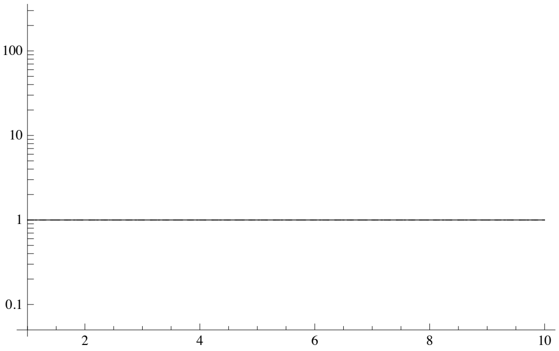

Beneficial fusions :

```
selterm = 0.0003;
subsel = {sX → 2 / 3 selterm, sY → 10^(-12), sW → selterm, sZ → 1 / 3 selterm};
plot3 = Show[
  LogPlot[ratioY /. Nf → N / 2 /. Nm → 1 / γ N / 2 /. N → 10 000 /. subsel /. OriginBySex /. α → 1,
   {γ, 1, 10}, PlotStyle → Blue, PlotRange → All, AxesOrigin → {1, Log[0.05]}],
  LogPlot[ratioW /. Nf → N / 2 /. Nm → 1 / γ N / 2 /. N → 10 000 /. subsel /. OriginBySex /. α → 1,
   {γ, 1, 10}, PlotStyle → {Blue, Dashed}, PlotRange → All],
  LogPlot[ratioZ /. Nf → N / 2 /. Nm → 1 / γ N / 2 /. N → 10 000 /. subsel /. OriginBySex /. α → 1,
   {γ, 1, 10}, PlotStyle → {Blue, Dotted}, PlotRange → All],
  PlotRange → {{1, 10}, {Log[0.05], Log[300]}}, Ticks → tickset
 ]
```

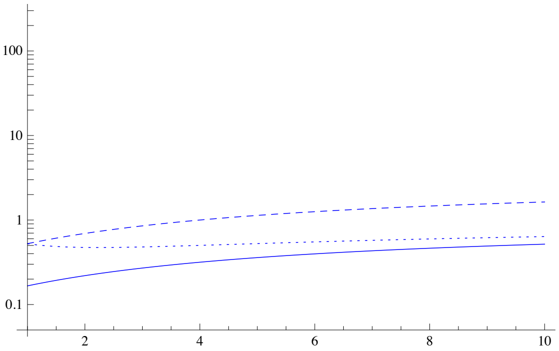

Altogether:

```
Show[plot1, plot2, plot3, AxesLabel → {"Nf/Nm", "Relative establishment rate"}]
```

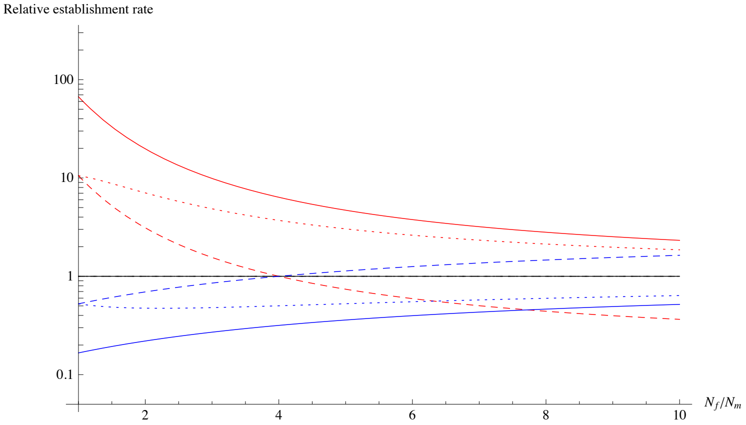

- **Figure - axes in both directions (meiotic drive against fusions, f < 0, is needed to explain Y-A excess)**

```
tickset =
  {Join[{{Log[0.1], 0.1}}, {{Log[1], 1}}, {{Log[10], 10}}, Flatten[Table[{Log[j 10^i], ""}, {i, -1., 3}, {j, 2, 9}], 1]],
   Join[{{Log[0.1], 0.1}}, Table[{Log[10^i], 10^i}, {i, 0, 3}],
    Flatten[Table[{Log[j 10^i], ""}, {i, -1., 3}, {j, 2, 9}], 1]]};
```

```
OriginBySex = {μ_X^f → μ^f, μ_X^m → μ^m, μ_Y → μ^m, μ_Z^m → μ^m, μ_Z^f → μ^f, μ_W → μ^f} /. μ^m → α μ^f;
```

Deleterious fusions:

```
selterm = -0.0003;
subsel = {s_X → 2 / 3 selterm, s_Y → 10^(-12), s_W → selterm, s_Z → 1 / 3 selterm};
plot1 = Show[
  LogLogPlot[ratio_Y /. N_f → N / 2 /. N_m → 1 / γ N / 2 /. N → 10 000 /. subsel /. OriginBySex /. α → 1,
    {γ, 1 / 10, 10}, PlotStyle → Red, PlotRange → All, AxesOrigin → {Log[1], Log[0.05]}],
  LogLogPlot[ratio_W /. N_f → N / 2 /. N_m → 1 / γ N / 2 /. N → 10 000 /. subsel /. OriginBySex /. α → 1,
    {γ, 1 / 10, 10}, PlotStyle → {Red, Dashed}, PlotRange → All],
  LogLogPlot[ratio_Z /. N_f → N / 2 /. N_m → 1 / γ N / 2 /. N → 10 000 /. subsel /. OriginBySex /. α → 1,
    {γ, 1 / 10, 10}, PlotStyle → {Red, Dotted}, PlotRange → All],
  PlotRange → {{Log[1 / 10], Log[10]}, {Log[0.05], Log[300]}},
  Ticks → tickset
 ]
```

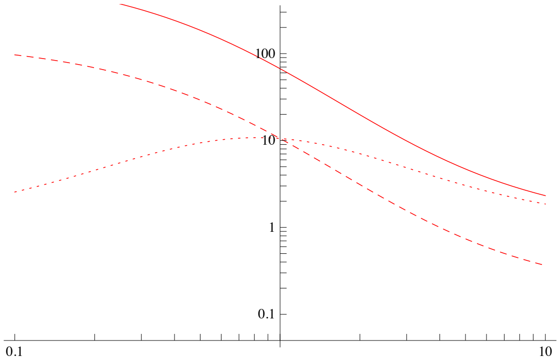

Neutral fusions:

```
selterm = 10^(-12);
subsel = {s_X → 2 / 3 selterm, s_Y → 10^(-12), s_W → selterm, s_Z → 1 / 3 selterm};
plot2 = Show[
  LogLogPlot[ratio_Y /. N_f → N / 2 /. N_m → 1 / γ N / 2 /. N → 10 000 /. subsel /. OriginBySex /. α → 1,
    {γ, 1 / 10, 10}, PlotStyle → Black, PlotRange → All, AxesOrigin → {Log[1], Log[0.05]}],
  LogLogPlot[ratio_W /. N_f → N / 2 /. N_m → 1 / γ N / 2 /. N → 10 000 /. subsel /. OriginBySex /. α → 1,
    {γ, 1 / 10, 10}, PlotStyle → {Black, Dashed}, PlotRange → All],
  LogLogPlot[ratio_Z /. N_f → N / 2 /. N_m → 1 / γ N / 2 /. N → 10 000 /. subsel /. OriginBySex /. α → 1,
    {γ, 1 / 10, 10}, PlotStyle → {Black, Dotted}, PlotRange → All],
  PlotRange → {{Log[1 / 10], Log[10]}, {Log[0.05], Log[300]}},
  Ticks → tickset
 ]
```

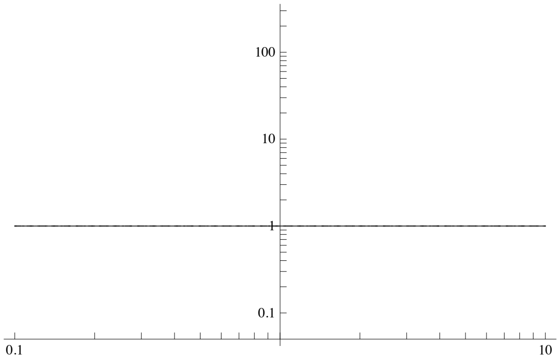

Beneficial fusions :

```
selterm = 0.0003;
subsel = {s_X → 2 / 3 selterm, s_Y → 10^(-12), s_W → selterm, s_Z → 1 / 3 selterm};
plot3 = Show[
  LogLogPlot[ratio_Y /. N_f → N / 2 /. N_m → 1 / γ N / 2 /. N → 10 000 /. subsel /. OriginBySex /. α → 1,
   {γ, 1 / 10, 10}, PlotStyle → Blue, PlotRange → All, AxesOrigin → {Log[1], Log[0.05]}],
  LogLogPlot[ratio_W /. N_f → N / 2 /. N_m → 1 / γ N / 2 /. N → 10 000 /. subsel /. OriginBySex /. α → 1,
   {γ, 1 / 10, 10}, PlotStyle → {Blue, Dashed}, PlotRange → All],
  LogLogPlot[ratio_Z /. N_f → N / 2 /. N_m → 1 / γ N / 2 /. N → 10 000 /. subsel /. OriginBySex /. α → 1,
   {γ, 1 / 10, 10}, PlotStyle → {Black, Dotted}, PlotRange → All],
  PlotRange → {{Log[1 / 10], Log[10]}, {Log[0.05], Log[300]}},
  Ticks → tickset
 ]
```

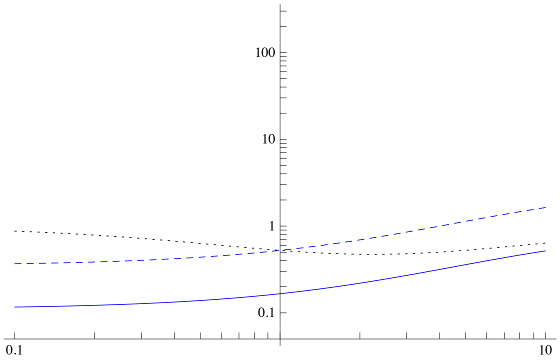

Altogether:

```
Show[plot1, plot2, plot3, AxesLabel → {"N_f/N_m", "Relative establishment rate"}]
```

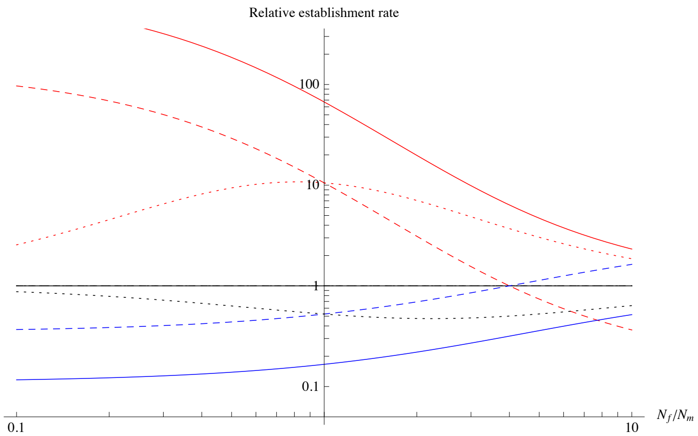

- **Figure - Net establishment rate for X-A and Y-A versus for W-A and Z-A**

Here we explore the relative ratio of XY fusions versus ZW fusions, accounting for sex ratio biases (selection on Y-A fusions is set to 1/N^2 to avoid numerical errors when setting it to 0, but selection will be effectively zero):

```
OriginBySex = {μ_X^f → μ^f, μ_X^m → μ^m, μ_Y → μ^m, μ_Z^m → μ^m, μ_Z^f → μ^f, μ_W → μ^f} /. μ^m → α μ^f;

subsel = {s_X → 2 / 3 f, s_Y → 1 / N^2, s_W → f, s_Z → 1 / 3 f};
XYvsZW[f_, N_, γ_] =
 (ratio_Y + 1) / (ratio_W + ratio_Z) /. N_f → N / 2 /. N_m → 1 / γ N / 2 /. subsel /. OriginBySex /. α → 1 // Simplify
```

$$\left(e^{-\frac{2f(2-3N+2\gamma)}{2+\gamma}}\left(-e^{\frac{4f}{2+\gamma}}-2e^{\frac{4f\gamma}{2+\gamma}}\gamma+e^{\frac{4f(1+\gamma)}{2+\gamma}}(1+2\gamma)\right)\left(1+\left(e^{-\frac{2}{N^2}+\frac{1}{N\gamma}-\frac{6fN}{2+\gamma}+\frac{4f(1+\gamma)}{2+\gamma}}\left(-1+e^{\frac{2}{N^2}}\right)\left(-1+e^{\frac{6fN}{2+\gamma}}\right)\right)\Big/\left(\left(-1+e^{\frac{1}{N\gamma}}\right)\left(-e^{\frac{4f}{2+\gamma}}-2e^{\frac{4f\gamma}{2+\gamma}}\gamma+e^{\frac{4f(1+\gamma)}{2+\gamma}}(1+2\gamma)\right)\right)\right)\right)\Big/\left(\left(-1+e^{\frac{6fN}{2+\gamma}}\right)\gamma\left(\frac{1-e^{-2f}}{1-e^{-fN}}+\left(e^{\frac{f(3N-2(1+\gamma))}{1+2\gamma}}\left(-2e^{\frac{2f}{1+2\gamma}}-e^{\frac{2f\gamma}{1+2\gamma}}\gamma+e^{\frac{2f(1+\gamma)}{1+2\gamma}}(2+\gamma)\right)\right)\Big/\left(\left(-1+e^{\frac{3fN}{1+2\gamma}}\right)\gamma\right)\right)\right)$$

As long the mutations are deleterious, fusions are expected in XY lineages more often than ZW lineages:

```
selterm = -0.0003;
subsel = {sX → 2 / 3 selterm, sY → 10^(-12), sW → selterm, sZ → 1 / 3 selterm};
plot1 = Show[
  LogLogPlot[XYvsZW[selterm, 10 000, γ], {γ, 1 / 10, 10}, PlotStyle → {Green}, AxesOrigin → {Automatic, Log[1]}],
  PlotRange → {{Log[1 / 10], Log[10]}, {Log[0.1], Log[100]}},
  Ticks → tickset
 ]
```

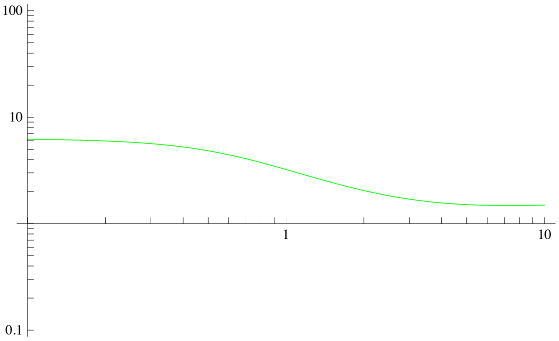

- ## Sex-specific origination rates

- **Figure (meiotic drive against fusions, f < 0, is needed to explain Y-A excess when $\mu_m > \mu_f$)**

```
tickset = {Automatic, Join[{{Log[0.1], 0.1}},
    Table[{Log[10^i], 10^i}, {i, 0, 3}], Flatten[Table[{Log[j 10^i], ""}, {i, -1., 3}, {j, 2, 9}], 1]]};
```

```
OriginBySex = {μXf → μf, μXm → μm, μY → μm, μZm → μm, μZf → μf, μW → μf} /. μm → α μf;
```

Deleterious fusions:

```
selterm = -0.0003;
subsel = {sX → 2 / 3 selterm, sY → 10^(-12), sW → selterm, sZ → 1 / 3 selterm};
plot1 = Show[
  LogPlot[ratioY /. Nf → N / 2 /. Nm → N / 2 /. N → 10 000 /. subsel /. OriginBySex,
   {α, 1, 10}, AxesOrigin → {1, Log[0.05]}, PlotStyle → Red],
  LogPlot[ratioW /. Nf → N / 2 /. Nm → N / 2 /. N → 10 000 /. subsel /. OriginBySex, {α, 1, 10}, PlotStyle → {Red, Dashed}],
  LogPlot[ratioZ /. Nf → N / 2 /. Nm → N / 2 /. N → 10 000 /. subsel /. OriginBySex, {α, 1, 10}, PlotStyle → {Red, Dotted}],
  PlotRange → {{1, 10}, {Log[0.05], Log[300]}},
  Ticks → tickset
 ]
```

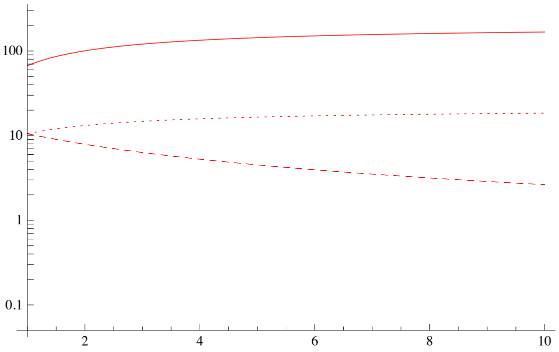

Neutral fusions:

```
selterm = 10^(-12);
subsel = {s_X → 2 / 3 selterm, s_Y → 10^(-12), s_W → selterm, s_Z → 1 / 3 selterm};
plot2 = Show[
  LogPlot[ratio_Y /. N_f → N / 2 /. N_m → N / 2 /. N → 10 000 /. subsel /. OriginBySex,
   {α, 1, 10}, AxesOrigin → {1, Log[0.05]}, PlotStyle → Black],
  LogPlot[ratio_W /. N_f → N / 2 /. N_m → N / 2 /. N → 10 000 /. subsel /. OriginBySex, {α, 1, 10}, PlotStyle → {Black, Dashed}],
  LogPlot[ratio_Z /. N_f → N / 2 /. N_m → N / 2 /. N → 10 000 /. subsel /. OriginBySex, {α, 1, 10}, PlotStyle → {Black, Dotted}],
  PlotRange → {{1, 10}, {Log[0.05], Log[300]}},
  Ticks → tickset
 ]
```

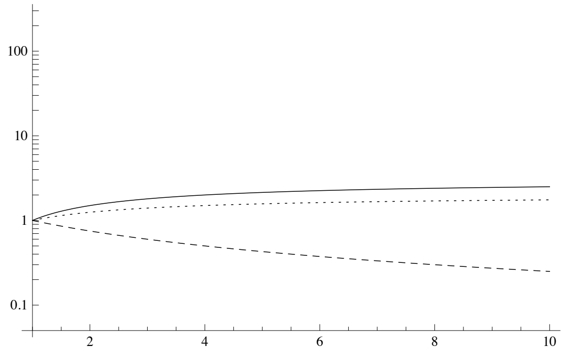

Beneficial fusions :

```
selterm = 0.0003;
subsel = {s_X → 2 / 3 selterm, s_Y → 10^(-12), s_W → selterm, s_Z → 1 / 3 selterm};
plot3 = Show[
  LogPlot[ratio_Y /. N_f → N / 2 /. N_m → N / 2 /. N → 10 000 /. subsel /. OriginBySex,
   {α, 1, 10}, AxesOrigin → {1, Log[0.05]}, PlotStyle → Blue],
  LogPlot[ratio_W /. N_f → N / 2 /. N_m → N / 2 /. N → 10 000 /. subsel /. OriginBySex, {α, 1, 10}, PlotStyle → {Blue, Dashed}],
  LogPlot[ratio_Z /. N_f → N / 2 /. N_m → N / 2 /. N → 10 000 /. subsel /. OriginBySex, {α, 1, 10}, PlotStyle → {Blue, Dotted}],
  PlotRange → {{1, 10}, {Log[0.05], Log[300]}},
  Ticks → tickset
 ]
```

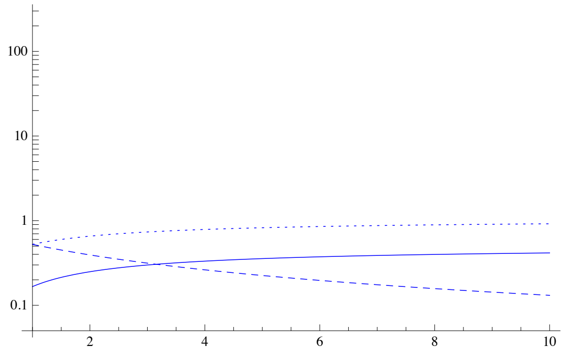

Altogether:

```
Show[plot1, plot3, plot2, AxesLabel → {"α", "Relative establishment rate"}]
```

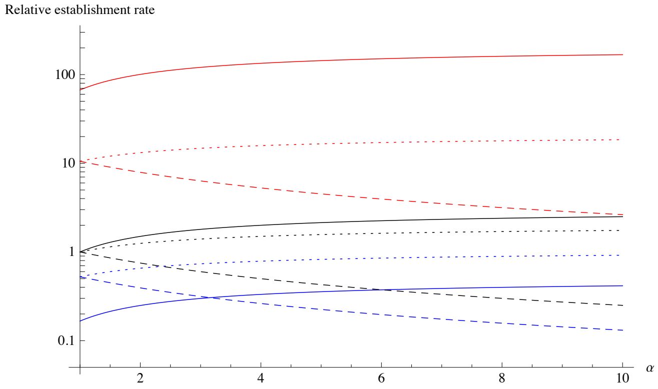

- **Figure - axes in both directions (meiotic drive against fusions, f < 0, and or $\mu_m > \mu_f$ is needed to explain Y-A excess)**

```
tickset =
  {Join[{{Log[0.1], 0.1}}, {{Log[1], 1}}, {{Log[10], 10}}, Flatten[Table[{Log[j 10^i], ""}, {i, -1., 3}, {j, 2, 9}], 1]],
   Join[{{Log[0.1], 0.1}}, Table[{Log[10^i], 10^i}, {i, 0, 3}],
    Flatten[Table[{Log[j 10^i], ""}, {i, -1., 3}, {j, 2, 9}], 1]]};
```

```
OriginBySex = {μ^f_X → μ^f, μ^m_X → μ^m, μ_Y → μ^m, μ^m_Z → μ^m, μ^f_Z → μ^f, μ_W → μ^f} /. μ^m → α μ^f;
```

Deleterious fusions:

```
selterm = -0.0003;
subsel = {sX → 2 / 3 selterm, sY → 10^(-12), sW → selterm, sZ → 1 / 3 selterm};
plot1 = Show[
  LogLogPlot[ratioY /. Nf → N / 2 /. Nm → N / 2 /. N → 10000 /. subsel /. OriginBySex,
   {α, 1 / 10, 10}, AxesOrigin → {Log[1], Log[0.05]}, PlotStyle → Red],
  LogLogPlot[ratioW /. Nf → N / 2 /. Nm → N / 2 /. N → 10000 /. subsel /. OriginBySex,
   {α, 1 / 10, 10}, PlotStyle → {Red, Dashed}], LogLogPlot[
   ratioZ /. Nf → N / 2 /. Nm → N / 2 /. N → 10000 /. subsel /. OriginBySex, {α, 1 / 10, 10}, PlotStyle → {Red, Dotted}],
  PlotRange → {{Log[1 / 10], Log[10]}, {Log[0.05], Log[300]}},
  Ticks → tickset
 ]
```

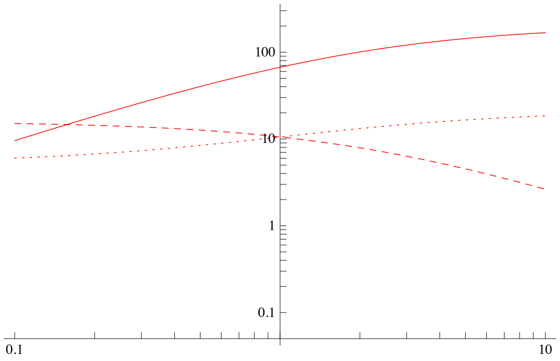

Neutral fusions:

```
selterm = 10^(-12);
subsel = {sX → 2 / 3 selterm, sY → 10^(-12), sW → selterm, sZ → 1 / 3 selterm};
plot2 = Show[
  LogLogPlot[ratioY /. Nf → N / 2 /. Nm → N / 2 /. N → 10000 /. subsel /. OriginBySex,
   {α, 1 / 10, 10}, AxesOrigin → {Log[1], Log[0.05]}, PlotStyle → Black],
  LogLogPlot[ratioW /. Nf → N / 2 /. Nm → N / 2 /. N → 10000 /. subsel /. OriginBySex,
   {α, 1 / 10, 10}, PlotStyle → {Black, Dashed}], LogLogPlot[
   ratioZ /. Nf → N / 2 /. Nm → N / 2 /. N → 10000 /. subsel /. OriginBySex, {α, 1 / 10, 10}, PlotStyle → {Black, Dotted}],
  PlotRange → {{Log[1 / 10], Log[10]}, {Log[0.05], Log[300]}},
  Ticks → tickset
 ]
```

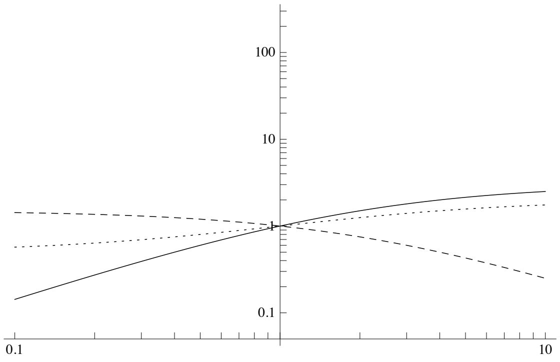

Beneficial fusions :

```
selterm = 0.0003;
subsel = {sX → 2 / 3 selterm, sY → 10 ^ (-12), sW → selterm, sZ → 1 / 3 selterm};
plot3 = Show[
  LogLogPlot[ratioY /. Nf → N / 2 /. Nm → N / 2 /. N → 10 000 /. subsel /. OriginBySex,
   {α, 1 / 10, 10}, AxesOrigin → {Log[1], Log[0.05]}, PlotStyle → Blue],
  LogLogPlot[ratioW /. Nf → N / 2 /. Nm → N / 2 /. N → 10 000 /. subsel /. OriginBySex,
   {α, 1 / 10, 10}, PlotStyle → {Blue, Dashed}], LogLogPlot[
   ratioZ /. Nf → N / 2 /. Nm → N / 2 /. N → 10 000 /. subsel /. OriginBySex, {α, 1 / 10, 10}, PlotStyle → {Blue, Dotted}],
  PlotRange → {{Log[1 / 10], Log[10]}, {Log[0.05], Log[300]}},
  Ticks → tickset
 ]
```

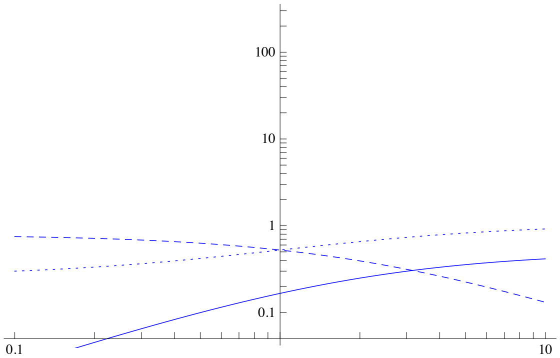

Altogether:

```
Show[plot1, plot3, plot2, AxesLabel → {"α", "Relative establishment rate"}]
```

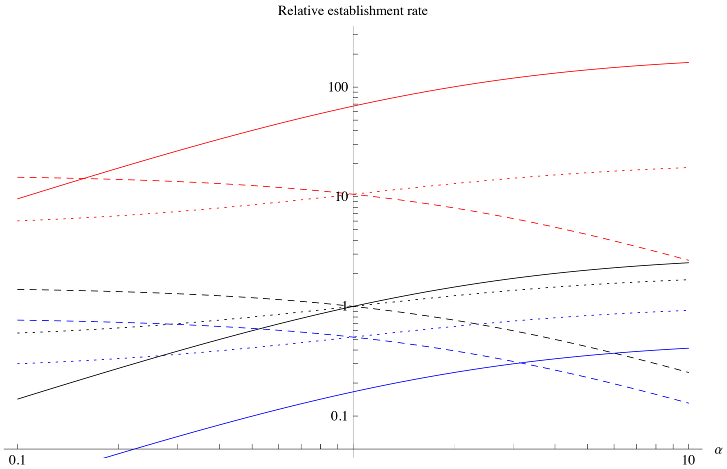

- **Figure - Net establishment rate for X-A and Y-A versus for W-A and Z-A**

```
tickset =
  {Join[{{Log[0.1], 0.1}}, {{Log[1], 1}}, {{Log[10], 10}}, Flatten[Table[{Log[j 10^i], ""}, {i, -1., 3}, {j, 2, 9}], 1]],
   Join[{{Log[0.1], 0.1}}, Table[{Log[10^i], 10^i}, {i, 0, 3}],
    Flatten[Table[{Log[j 10^i], ""}, {i, -1., 3}, {j, 2, 9}], 1]]};
```

Here we explore the relative ratio of XY fusions versus ZW fusions, accounting for sex ratio biases:

```
OriginBySex = {μX^f → μ^f, μX^m → μ^m, μY → μ^m, μZ^m → μ^m, μZ^f → μ^f, μW → μ^f} /. μ^m → α μ^f;

subsel = {sX → 2 / 3 f, sY → 1 / N^2, sW → f, sZ → 1 / 3 f};

XYvsZW[f_, N_, α_] = (ratioY + 1) / (ratioW + ratioZ) /. Nf → N / 2 /. Nm → N / 2 /. subsel /. OriginBySex // Simplify
```

$$-\left(e^{2 f+\frac{1}{N}-f N} \alpha - e^{-\frac{(-2+N)\left(-1+f N^2\right)}{N^2}} \alpha + e^{\frac{-2+N}{N^2}+f (2+N)} \alpha - 2 e^{\frac{1}{N}+f (2+N)} (1+\alpha) - e^{f \left(\frac{2}{3}+N\right)} (2+\alpha) + e^{f (2+N)} (2+\alpha) + e^{\frac{1}{N}+f \left(\frac{2}{3}+N\right)} (2+\alpha)\right) \Big/ \left(\left(-1+e^{2 f/3}\right)\left(-1+e^{\frac{1}{N}}\right)\left(1+e^{f N}\right)\left(1+e^{2 f/3}+2 e^{4 f/3} (1+\alpha)\right)\right)$$

As long as α is not too small and the mutations are deleterious, fusions are expected in XY lineages more often than ZW lineages:

```
selterm = -0.0003;
subsel = {sX → 2 / 3 selterm, sY → 10^(-12), sW → selterm, sZ → 1 / 3 selterm};
plot1 = Show[
  LogLogPlot[XYvsZW[selterm, 10 000, α], {α, 1 / 10, 10}, PlotStyle → {Green}, AxesOrigin → {Automatic, Log[1]}],
  PlotRange → {{Log[1 / 10], Log[10]}, {Log[0.1], Log[100]}},
  Ticks → tickset
 ]
```

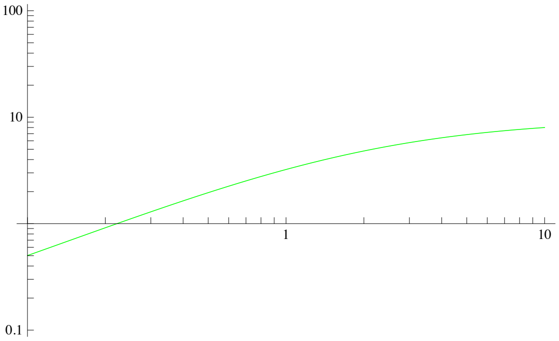

- **Allowing both biased sex-ratios and sex-specific origination rates**

```
tickset =
  {Join[Table[{i, i}, {i, -0.001, 0.001, 0.0005}], Flatten[Table[{Log[j 10^i], ""}, {i, -1., 3}, {j, 2, 9}], 1]], Join[
    {{Log[0.1], 0.1}}, Table[{Log[10^i], 10^i}, {i, 0, 3}], Flatten[Table[{Log[j 10^i], ""}, {i, -1., 3}, {j, 2, 9}], 1]]};
subsel = {sX → 2 / 3 f, sY → 1 / N^2, sW → f, sZ → 1 / 3 f};
Manipulate[Show[
  LogPlot[ratioY /. Nf → N / 2 /. Nm → (1/γ) N / 2 /. subsel /. N → 10 000 /. {μXf → μf, μXm → μm, μY → μm, μZm → μm, μZf → μf, μW → μf} /.
    μm → α μf, {f, -0.001, 0.001}, AxesOrigin → {0, Log[0.05]}, PlotStyle → Black],
  LogPlot[ratioW /. Nf → N / 2 /. Nm → (1/γ) N / 2 /. subsel /. N → 10 000 /. {μXf → μf, μXm → μm, μY → μm, μZm → μm, μZf → μf, μW → μf} /.
    μm → α μf, {f, -0.001, 0.001}, PlotStyle → {Black, Dashed}], LogPlot[
   ratioZ /. Nf → N / 2 /. Nm → (1/γ) N / 2 /. subsel /. N → 10 000 /. {μXf → μf, μXm → μm, μY → μm, μZm → μm, μZf → μf, μW → μf} /. μm → α μf,
   {f, -0.001, 0.001}, PlotStyle → {Black, Dotted}],
  PlotRange → {{-0.001, 0.001}, {Log[0.05], Log[1000]}},
  Ticks → tickset, AxesLabel → {"f", "Relative establishment rate"}], {{α, 1}, 0.1, 10}, {{γ, 1}, 0.1, 10}]
```

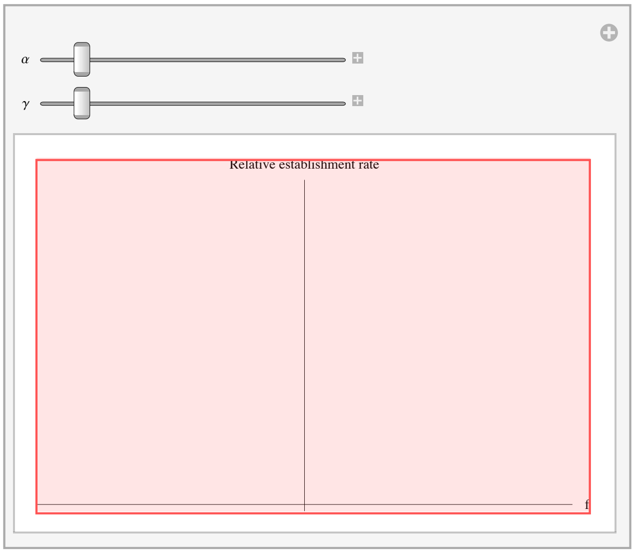

ReplaceAll::reps : {subsel} is neither a list of replacement rules nor a valid dispatch table, and so cannot be used for replacing. ≫

ReplaceAll::reps : {subsel} is neither a list of replacement rules nor a valid dispatch table, and so cannot be used for replacing. ≫

ReplaceAll::reps : {subsel} is neither a list of replacement rules nor a valid dispatch table, and so cannot be used for replacing. ≫

General::stop : Further output of ReplaceAll::reps will be suppressed during this calculation. ≫

Fusions driven by SA selection

■ Introduction and derivation

Here we explore sexually antagonistic selection driving the spread of fusions that couple sex-beneficial alleles to that sex. Locus A, with alleles A_0 and A_1, is under SA selection on an autosome. Allele A_0 is beneficial in males and A_1 in females. The equilibrium frequency of allele A_1 is p. We assume that SA selection is sufficiently weak that allele frequency differences between eggs and sperm, and between gametes and adults, can be neglected. We make no assumptions about the form of selection leading to that equilibrium. In addition, we assume that there is no recombination between the SDR and the fusion. (This assumption is justified by the fact that we are trying to explain systems in which one but not both sex chromosomes are fused.) In this section, we also ignore recombination between locus A and the SDR in fused chromosomes.

Define the selection coefficients

$$s_i^m = \frac{W_{m,i}}{\overline{W}_m} - 1$$

$$s_i^f = \frac{W_{f,i}}{\overline{W}_f} - 1$$

where $W_{s,i}$ is the average fitness of allele A_i in sex s, and $\overline{W}_s$ is the mean fitness of that sex in the population before the fusion appears. Note that these selection coefficients will in general depend on allele frequencies, so they will change if the fusion spreads to a nontrivial frequency.

Assuming that sexually antagonistic selection is weak, we can again take an average of the resulting selection on the fusion over many generations:

$$s_{X,i} = \frac{2}{3} s_i^f + \frac{1}{3} s_i^m$$
$$s_{Y,i} = s_i^m$$
$$s_{Z,i} = \frac{1}{3} s_i^f + \frac{2}{3} s_i^m$$
$$s_{W,i} = s_i^f$$

In addition, we must now account for the probability that the fusion appears with allele A_i. For example, take A_0 to be the male beneficial allele and A_1 to be the female beneficial allele, with p_i equal to the frequency of allele A_i, the rate of establishment for a fusion that captures allele i is derived as before, but is now multiplied by p_i:

YA: YA fusions with allele i establish at rate $\mu_Y\, p_i\, N_m \frac{1-\text{Exp}[-2\, s_{Y,i}]}{1-\text{Exp}[-2\, N_m\, s_{Y,i}]}$

XA: XA fusions with allele i establish at rate $2\,\mu_X^f\, p_i\, N_f \frac{1-\text{Exp}\left[-2\,(N_{e,X})\left(\frac{2}{3}\frac{1}{2 N_f}\right) s_{X,i}\right]}{1-\text{Exp}[-2\,(N_{e,X})\, s_{X,i}]} + \mu_X^m\, p_i\, N_m \frac{1-\text{Exp}\left[-2\,(N_{e,X})\left(\frac{1}{3}\frac{1}{N_m}\right) s_{X,i}\right]}{1-\text{Exp}[-2\,(N_{e,X})\, s_{X,i}]}$.

WA: WA fusions with allele i establish at rate $\mu_W\, p_i\, N_f \frac{1-\text{Exp}[-2\, s_{W,i}]}{1-\text{Exp}[-2\, N_f\, s_{W,i}]}$.

ZA: ZA fusions with allele i establish at rate $\mu_Z^f\, p_i\, N_f \frac{1-\text{Exp}\left[-2\,(N_{e,Z})\left(\frac{1}{3}\frac{1}{N_f}\right) s_{Z,i}\right]}{1-\text{Exp}[-2\,(N_{e,Z})\, s_{Z,i}]} + 2\,\mu_Z^m\, p_i\, N_m \frac{1-\text{Exp}\left[-2\,(N_{e,Z})\left(\frac{2}{3}\frac{1}{2 N_m}\right) s_{Z,i}\right]}{1-\text{Exp}[-2\,(N_{e,Z})\, s_{Z,i}]}$.

Summing over all of the alleles gives the net rate of establishment.

$$N_{e,X} = \frac{9\, N_f\, N_m}{N_f + 2\, N_m};$$

$$N_{e,Z} = \frac{9\, N_f\, N_m}{2\, N_f + N_m};$$

$$R_Y = \sum_{i=0}^{1}\left(\mu_Y\, p_i\, N_m \frac{1 - \text{Exp}[-2\, s_{Y,i}]}{1 - \text{Exp}[-2\, N_m\, s_{Y,i}]}\right);$$

$$R_X = \sum_{i=0}^{1}\left(2\,\mu_X^f\, p_i\, N_f \frac{1 - \text{Exp}\left[-2\,(N_{e,X})\left(\frac{2}{3}\frac{1}{2 N_f}\right) s_{X,i}\right]}{1 - \text{Exp}[-2\,(N_{e,X})\, s_{X,i}]} + \mu_X^m\, p_i\, N_m \frac{1 - \text{Exp}\left[-2\,(N_{e,X})\left(\frac{1}{3}\frac{1}{N_m}\right) s_{X,i}\right]}{1 - \text{Exp}[-2\,(N_{e,X})\, s_{X,i}]}\right);$$

$$R_W = \sum_{i=0}^{1}\left(\mu_W\, p_i\, N_f \frac{1 - \text{Exp}[-2\, s_{W,i}]}{1 - \text{Exp}[-2\, N_f\, s_{W,i}]}\right);$$

$$R_Z = \sum_{i=0}^{1}\left(\mu_Z^f\, p_i\, N_f \frac{1 - \text{Exp}\left[-2\,(N_{e,Z})\left(\frac{1}{3}\frac{1}{N_f}\right) s_{Z,i}\right]}{1 - \text{Exp}[-2\,(N_{e,Z})\, s_{Z,i}]} + 2\,\mu_Z^m\, p_i\, N_m \frac{1 - \text{Exp}\left[-2\,(N_{e,Z})\left(\frac{2}{3}\frac{1}{2 N_m}\right) s_{Z,i}\right]}{1 - \text{Exp}[-2\,(N_{e,Z})\, s_{Z,i}]}\right);$$

The ratio of Y-A origination relative to X-A establishment rates is therefore

$$\text{ratio}_Y = \frac{R_Y}{R_X};$$

Similarly, the rate of origination for W-A fusions relative to X-A fusions would be:

$$\text{ratio}_W = \frac{R_W}{R_X};$$

and the rate of origination for Z-A fusions relative to X-A fusions would be:

$$\text{ratio}_Z = \frac{R_Z}{R_X};$$

In the next section, we calculate p_i and s_i^{sex}, assuming weak selection:

$$\text{subequil} = \left\{p_0 \to 1 - \frac{(1-\text{hm})\,\text{sm} - \text{hf}\,\text{sf}}{\text{sf} - 2\,\text{hf}\,\text{sf} + \text{sm} - 2\,\text{hm}\,\text{sm}},\ p_1 \text{->} \frac{(1-\text{hm})\,\text{sm} - \text{hf}\,\text{sf}}{\text{sf} - 2\,\text{hf}\,\text{sf} + \text{sm} - 2\,\text{hm}\,\text{sm}}\right\};$$

$$\text{submarg} = \left\{s_0^m \to \frac{(1-\text{hf}-\text{hm})\,\text{sf}\,\text{sm}\,(\text{sm}\,(1-\text{hm}) - \text{hf}\,\text{sf})}{(\text{sf}\,(1-2\,\text{hf}) + \text{sm}\,(1-2\,\text{hm}))^2},\ s_1^m \to -\frac{(1-\text{hf}-\text{hm})\,\text{sf}\,\text{sm}\,(\text{sf}\,(1-\text{hf}) - \text{hm}\,\text{sm})}{(\text{sf}\,(1-2\,\text{hf}) + \text{sm}\,(1-2\,\text{hm}))^2},\right.$$
$$\left. s_0^f \to -\frac{(1-\text{hf}-\text{hm})\,\text{sf}\,\text{sm}\,(\text{sm}\,(1-\text{hm}) - \text{hf}\,\text{sf})}{(\text{sf}\,(1-2\,\text{hf}) + \text{sm}\,(1-2\,\text{hm}))^2},\ s_1^f \to \frac{(1-\text{hf}-\text{hm})\,\text{sf}\,\text{sm}\,(\text{sf}\,(1-\text{hf}) - \text{hm}\,\text{sm})}{(\text{sf}\,(1-2\,\text{hf}) + \text{sm}\,(1-2\,\text{hm}))^2}\right\};$$

$$\text{subsel} = \left\{s_{Y,0} \to s_0^m,\ s_{Y,1} \to s_1^m,\ s_{X,0} \to \frac{2}{3} s_0^f + \frac{1}{3} s_0^m,\ s_{X,1} \to \frac{2}{3} s_1^f + \frac{1}{3} s_1^m,\ s_{Z,0} \to \frac{1}{3} s_0^f + \frac{2}{3} s_0^m,\ s_{Z,1} \to \frac{1}{3} s_1^f + \frac{2}{3} s_1^m,\ s_{W,0} \to s_0^f,\ s_{W,1} \to s_1^f\right\};$$

$$\text{OriginBySex} = \{\mu_X^f \to \mu^f,\ \mu_X^m \to \mu^m,\ \mu_Y \to \mu^m,\ \mu_Z^m \to \mu^m,\ \mu_Z^f \to \mu^f,\ \mu_W \to \mu^f\}\ /.\ \mu^m \to \alpha\,\mu^f;$$

If selection is sufficiently strong (N s>>1), fusions will only establish if they are linked to the right allele, specifically, the allele A_0 that is beneficial in males for Z and Y chromosomes and the allele A_1 in females for W and X chromosomes. Assuming a large population size and N s>>1 and dropping the very small establishment rates for any other combinations, we can approximate the above rates of establishment as:

$$\text{approxratio}_Y = (\mu_Y\, p_0\, N_m (2\, s_{Y,0})) \Big/ \left(2\,\mu_X^f\, p_1\, N_f \left(2\,(N_{e,X}) \left(\frac{2}{3} - \frac{1}{2\,N_f}\right) s_{X,1}\right) + \mu_X^m\, p_1\, N_m \left(2\,(N_{e,X}) \left(\frac{1}{3} - \frac{1}{N_m}\right) s_{X,1}\right)\right);$$

$$\text{approxratio}_W = (\mu_W\, p_1\, N_f\, (2\, s_{W,1})) \Big/ \left(2\,\mu_X^f\, p_1\, N_f \left(2\,(N_{e,X}) \left(\frac{2}{3} - \frac{1}{2\,N_f}\right) s_{X,1}\right) + \mu_X^m\, p_1\, N_m \left(2\,(N_{e,X}) \left(\frac{1}{3} - \frac{1}{N_m}\right) s_{X,1}\right)\right);$$

$$\text{approxratio}_Z = \left(\mu_Z^f\, p_0\, N_f \left(2\,(N_{e,Z}) \left(\frac{1}{3} - \frac{1}{N_f}\right) s_{Z,0}\right) + 2\,\mu_Z^m\, p_0\, N_m \left(2\,(N_{e,Z}) \left(\frac{2}{3} - \frac{1}{2\,N_m}\right) s_{Z,0}\right)\right) \Big/$$
$$\left(2\,\mu_X^f\, p_1\, N_f \left(2\,(N_{e,X}) \left(\frac{2}{3} - \frac{1}{2\,N_f}\right) s_{X,1}\right) + \mu_X^m\, p_1\, N_m \left(2\,(N_{e,X}) \left(\frac{1}{3} - \frac{1}{N_m}\right) s_{X,1}\right)\right);$$

Under these conditions, there is no effect of sexually antagonistic selection on the rate of establishment of fusions, once we plug in the equilibrium and assuming all else equal:

$$\{\text{approxratio}_Y,\ \text{approxratio}_W,\ \text{approxratio}_Z\}\ /.\ \text{subequil}\ /.\ \text{subsel}\ /.\ \text{submarg}\ /.\ N_f \to N/2\ /.\ N_m \to N/2\ /.\ \text{OriginBySex}\ /.\ \alpha \to 1\ //\ \text{Factor}$$

{1, 1, 1}

This can be shown generally using the fact that the marginal allele fitnesses at the initial equilibrium must be opposite in males and females for the allele to remain constant at the initial autosomal equilibrium and using the fact that $p_0\, s_0^m + p_1\, s_1^m = p_0 \left(\frac{W_{m,0}}{\overline{W}_m} - 1\right) + p_1 \left(\frac{W_{m,1}}{\overline{W}_m} - 1\right) = \frac{p_0\, W_{m,0} + p_1\, W_{m,1}}{\overline{W}_m} - 1 = 0$:

$$\{\text{approxratio}_Y,\ \text{approxratio}_W,\ \text{approxratio}_Z\}\ /.\ \text{subsel}\ /.\ N_f \to N/2\ /.\ N_m \to N/2\ /.\ \text{OriginBySex}\ /.\ \alpha \to 1\ /.\ s_1^f \to -s_1^m\ /.\ s_0^f \to -s_0^m\ /.\ p_0\, s_0^m \to -p_1\, s_1^m\ //\ \text{Factor}$$

{1, 1, 1}

Allowing for sex ratio and origination rate biases:

$$\{\text{approxratio}_Y,\ \text{approxratio}_W,\ \text{approxratio}_Z\}\ /.\ \text{subequil}\ /.\ \text{subsel}\ /.\ \text{submarg}\ /.\ N_f \to N/2\ /.\ N_m \to 1/\gamma\, N/2\ /.\ \text{OriginBySex}\ //\ \text{Factor}$$

$$\left\{\frac{\alpha\,(2+\gamma)}{(2+\alpha)\,\gamma},\ \frac{2+\gamma}{2+\alpha},\ \frac{(1+2\,\alpha)\,(2+\gamma)}{(2+\alpha)\,(1+2\,\gamma)}\right\}$$

which is the same as found by using the equilibrium conditions:

$$\{\text{approxratio}_Y,\ \text{approxratio}_W,\ \text{approxratio}_Z\}\ /.\ \text{subsel}\ /.\ N_f \to N/2\ /.\ N_m \to 1/\gamma\, N/2\ /.\ \text{OriginBySex}\ /.\ s_1^f \to -s_1^m\ /.\ s_0^f \to -s_0^m\ /.\ p_0\, s_0^m \to -p_1\, s_1^m\ //\ \text{Factor}$$

$$\left\{\frac{\alpha\,(2+\gamma)}{(2+\alpha)\,\gamma},\ \frac{2+\gamma}{2+\alpha},\ \frac{(1+2\,\alpha)\,(2+\gamma)}{(2+\alpha)\,(1+2\,\gamma)}\right\}$$

$$\%-1\ //\ \text{Factor}$$

$$\left\{\frac{2\,(\alpha-\gamma)}{(2+\alpha)\,\gamma},\ -\frac{\alpha-\gamma}{2+\alpha},\ \frac{3\,(\alpha-\gamma)}{(2+\alpha)\,(1+2\,\gamma)}\right\}$$

If the origination rates are equal (α=1), then Y-A fusions will be more common than X-A as long as γ<1 [higher reproductive population size of males].
If the sex ratio is equal (γ=1), then Y-A fusions will be more common than X-A as long as α>1 [higher origination rate in males].
More generally, the origination rate must satisfy $\alpha > \gamma$ for Y-A to establish more frequently than X-A, W-A, and Z-A fusions.

To also account for the fact that XY fusions occur more often than ZW fusions, we require that the following be greater than one:

$$\text{XYcond} = (\text{approxratio}_Y + 1)\ /\ (\text{approxratio}_W + \text{approxratio}_Z)\ /.\ \text{subequil}\ /.\ \text{subsel}\ /.\ \text{submarg}\ /.\ N_f \to N/2\ /.\ N_m \to 1/\gamma\ N/2\ /.\ \text{OriginBySex}\ //\ \text{Simplify}$$

$$\frac{(1+2\,\gamma)\,(\alpha+\gamma+\alpha\,\gamma)}{\gamma\,(2+\gamma)\,(1+\alpha+\gamma)}$$

```
Factor[% - 1]
```

$$-\frac{(-\alpha+\gamma)\ (1+\gamma+\gamma^2)}{\gamma\ (2+\gamma)\ (1+\alpha+\gamma)}$$

Thus to see YA fusions most often and to see XY fusions more than ZW fusions requires $\alpha > \gamma$:

$$\text{Reduce}\left[\left\{\frac{\alpha\ (2+\gamma)}{(2+\alpha)\ \gamma} > \text{Max}\left[1, \frac{2+\gamma}{2+\alpha}, \frac{(1+2\,\alpha)\ (2+\gamma)}{(2+\alpha)\ (1+2\,\gamma)}\right], \text{XYcond} > 1, \gamma > 0, \alpha > 0\right\}\right]$$

$\gamma > 0$ && $\alpha > \gamma$

Calculating the equilibrium allele frequencies, p_i, and marginal selection coefficients, s_i^{sex}

Because the A locus is initially autosomal, the genotype frequencies at birth will be the same for males and females:

$$\begin{pmatrix} x1 & A_0 / A_0 \\ x2 & A_0 / A_1 \\ x3 & A_1 / A_1 \end{pmatrix}$$

Natural selection, however, acts to differentiate the sexes. Consequently, the frequency of allele A_1 in female gametes p^f will be different from that in male gametes p^m.

Fitness in females (Fij):

$$\begin{pmatrix} F00 & F01 & F11 \\ 1 & 1+hf\,sf & 1+sf \end{pmatrix}$$

Fitness in males (Mij):

$$\begin{pmatrix} M00 & M01 & M11 \\ 1+sm & 1+hm\,sm & 1 \end{pmatrix}$$

```
subfit = {F00 → 1, F01 → 1 + hf sf, F11 → 1 + sf, M00 → 1 + sm, M01 → 1 + hm sm, M11 → 1};
```

Gametes come together, one from each sex, at random to give:

```
x1 = (1 - pf) * (1 - pm);
x2 = pf * (1 - pm) + (1 - pf) * pm;
x3 = pf * pm;
Factor[x1 + x2 + x3]
```

1

After selection, in females:

```
xs1 = x1 * F00 / (x1 * F00 + x2 * F01 + x3 * F11);

xs2 = x2 * F01 / (x1 * F00 + x2 * F01 + x3 * F11);

xs3 = x3 * F11 / (x1 * F00 + x2 * F01 + x3 * F11);
Factor[xs1 + xs2 + xs3]
```

1

After selection, in males:

```
ys1 = x1 * M00 / (x1 * M00 + x2 * M01 + x3 * M11);

ys2 = x2 * M01 / (x1 * M00 + x2 * M01 + x3 * M11);

ys3 = x3 * M11 / (x1 * M00 + x2 * M01 + x3 * M11);
Factor[ys1 + ys2 + ys3]
```

1

Finally, these parents reproduce to get the next generation of gametes:

```
nextpf = xs2 / 2 + xs3;

nextpm = ys2 / 2 + ys3;
```

At equilibrium, the allele frequencies in each sex must stay the same, which gives us the following two equilibrium relationships (both must equal zero at equilibrium):

```
eqn1 = Factor[nextpf - pf]
```

$$-\left(2\,F00\,pf - F01\,pf - 2\,F00\,pf^2 + 2\,F01\,pf^2 - F01\,pm - 2\,F00\,pf\,pm + 4\,F01\,pf\,pm - 2\,F11\,pf\,pm + 2\,F00\,pf^2\,pm - 4\,F01\,pf^2\,pm + 2\,F11\,pf^2\,pm\right) / \left(2\,(F00 - F00\,pf + F01\,pf - F00\,pm + F01\,pm + F00\,pf\,pm - 2\,F01\,pf\,pm + F11\,pf\,pm)\right)$$

```
eqn2 = Factor[nextpm - pm]
```

$$-\left(-M01\,pf + 2\,M00\,pm - M01\,pm - 2\,M00\,pf\,pm + 4\,M01\,pf\,pm - 2\,M11\,pf\,pm - 2\,M00\,pm^2 + 2\,M01\,pm^2 + 2\,M00\,pf\,pm^2 - 4\,M01\,pf\,pm^2 + 2\,M11\,pf\,pm^2\right) / \left(2\,(M00 - M00\,pf + M01\,pf - M00\,pm + M01\,pm + M00\,pf\,pm - 2\,M01\,pf\,pm + M11\,pf\,pm)\right)$$

The first equation could be solved for pm to plug into the second equation, but the result is a cubic (as well as pf = pm = 0 or pf = pm = 0):

```
Factor[eqn2 /. Solve[eqn1 == 0, pm]]
```

$$\Big\{-\Big((-1+pf)\,pf\,\big(2\,F00\,F01\,M00 - F01^2\,M00 - F00\,F01\,M01 - 8\,F00\,F01\,M00\,pf + 5\,F01^2\,M00\,pf + 4\,F00\,F11\,M00\,pf - 2\,F01\,F11\,M00\,pf + 2\,F00^2\,M01\,pf + 3\,F00\,F01\,M01\,pf - 2\,F00\,F11\,M01\,pf - F01\,F11\,M01\,pf - 2\,F00\,F01\,M11\,pf + F01^2\,M11\,pf + 10\,F00\,F01\,M00\,pf^2 - 8\,F01^2\,M00\,pf^2 - 8\,F00\,F11\,M00\,pf^2 + 6\,F01\,F11\,M00\,pf^2 - 4\,F00^2\,M01\,pf^2 - 2\,F00\,F01\,M01\,pf^2 + 6\,F00\,F11\,M01\,pf^2 + 2\,F01\,F11\,M01\,pf^2 - 2\,F11^2\,M01\,pf^2 + 6\,F00\,F01\,M11\,pf^2 - 4\,F01^2\,M11\,pf^2 - 4\,F00\,F11\,M11\,pf^2 + 2\,F01\,F11\,M11\,pf^2 - 4\,F00\,F01\,M00\,pf^3 + 4\,F01^2\,M00\,pf^3 + 4\,F00\,F11\,M00\,pf^3 - 4\,F01\,F11\,M00\,pf^3 + 2\,F00^2\,M01\,pf^3 - 4\,F00\,F11\,M01\,pf^3 + 2\,F11^2\,M01\,pf^3 - 4\,F00\,F01\,M11\,pf^3 + 4\,F01^2\,M11\,pf^3 + 4\,F00\,F11\,M11\,pf^3 - 4\,F01\,F11\,M11\,pf^3\big)\Big) \Big/ \Big(\big(-F01 - 2\,F00\,pf + 4\,F01\,pf - 2\,F11\,pf + 2\,F00\,pf^2 - 4\,F01\,pf^2 + 2\,F11\,pf^2\big)\big(F01\,M00 - 4\,F01\,M00\,pf + 2\,F11\,M00\,pf + 2\,F00\,M01\,pf + 5\,F01\,M00\,pf^2 - 4\,F11\,M00\,pf^2 - 4\,F00\,M01\,pf^2 + 2\,F11\,M01\,pf^2 + 2\,F00\,M11\,pf^2 - F01\,M11\,pf^2 - 2\,F01\,M00\,pf^3 + 2\,F11\,M00\,pf^3 + 2\,F00\,M01\,pf^3 - 2\,F11\,M01\,pf^3 - 2\,F00\,M11\,pf^3 + 2\,F01\,M11\,pf^3\big)\Big)\Big\}$$

For weak selection, we can take a Taylor series of the two equilibrium requirements. Starting to linear order, we find that the allele frequencies must be equal in the two sexes (to leading order):

```
Normal[Series[Factor[eqn1 /. subfit /. sf → sf * small /. sm → sm * small], {small, 0, 0}]] /. small → 1
```

$$\frac{1}{2}(-pf + pm)$$

```
Normal[Series[Factor[eqn2 /. subfit /. sf → sf * small /. sm → sm * small], {small, 0, 0}]] /. small → 1
```

$$\frac{pf - pm}{2}$$

We thus let pave equal the average allele frequency of allele A_1 across the two sexes, and pdif the difference (pf - pm), where pdif is assumed small. Taking the next order term in the power series then allows us to solve for the average allele frequency:

```
Normal[Series[
   Factor[eqn1 /. subfit /. sf → sf * small /. sm → sm * small /. pf → pave + pdif / 2 /. pm → pave - pdif / 2 /. pdif → pdif * small],
   {small, 0, 1}]] /. small → 1
```

$$-\frac{pdif}{2} + hf\,pave\,sf + pave^2\,sf - 3\,hf\,pave^2\,sf - pave^3\,sf + 2\,hf\,pave^3\,sf$$

```
Normal[Series[
   Factor[eqn2 /. subfit /. sf → sf * small /. sm → sm * small /. pf → pave + pdif / 2 /. pm → pave - pdif / 2 /. pdif → pdif * small],
   {small, 0, 1}]] /. small → 1
```

$$\frac{pdif}{2} - pave\,sm + hm\,pave\,sm + 2\,pave^2\,sm - 3\,hm\,pave^2\,sm - pave^3\,sm + 2\,hm\,pave^3\,sm$$

```
Solve[{%% == 0, % == 0}, {pave, pdif}] // Simplify
```

$$\Big\{\{pave \to 0,\ pdif \to 0\},\ \{pave \to 1,\ pdif \to 0\},\ \Big\{pave \to -\frac{hf\,sf + (-1 + hm)\,sm}{sf - 2\,hf\,sf + sm - 2\,hm\,sm},\ pdif \to -\big(2\,(-1 + hf + hm)\,sf\,sm\,\big(hf^2\,sf^2 - hf\,sf\,(sf + sm - 2\,hm\,sm) + (-1 + hm)\,sm\,(-sf + hm\,sm)\big)\big) \big/ (sf - 2\,hf\,sf + sm - 2\,hm\,sm)^3\Big\}\Big\}$$

Thus, with weak selection, the average allele frequency is $pave = \frac{(1-hm)\,sm - hf\,sf}{sf\,(1-2\,hf) + sm\,(1-2\,hm)}$.

We can also derive the conditions under which the two fixation states are unstable, in which case the system moves into this polymorphic equilibrium:

```
mat = {{D[nextpf, pf], D[nextpf, pm]},
     {D[nextpm, pf], D[nextpm, pm]}} /. pf → 0 /. pm → 0 // Factor
```

$$\Big\{\Big\{\frac{F01}{2\,F00}, \frac{F01}{2\,F00}\Big\}, \Big\{\frac{M01}{2\,M00}, \frac{M01}{2\,M00}\Big\}\Big\}$$

```
Eigenvalues[mat] // Factor
```

$$\Big\{0, \frac{F01\,M00 + F00\,M01}{2\,F00\,M00}\Big\}$$

Thus, instability of the pf = pm = 0 equilibrium requires:

$$\frac{1}{2}\left(\frac{F01}{F00} + \frac{M01}{M00}\right) > 1$$

```
mat = {{D[nextpf, pf], D[nextpf, pm]},
     {D[nextpm, pf], D[nextpm, pm]}} /. pf → 1 /. pm → 1 // Factor
```

$$\Big\{\Big\{\frac{F01}{2\,F11}, \frac{F01}{2\,F11}\Big\}, \Big\{\frac{M01}{2\,M11}, \frac{M01}{2\,M11}\Big\}\Big\}$$

```
Eigenvalues[mat] // Factor
```

$$\Big\{0, \frac{F11\,M01 + F01\,M11}{2\,F11\,M11}\Big\}$$

Thus, instability of the pf = pm = 1 equilibrium requires:

$$\frac{1}{2}\left(\frac{F01}{F11} + \frac{M01}{M11}\right) > 1$$

Thus, a protected polymorphism exists only if

$$\mathrm{Min}\left[\frac{1}{2}\left(\frac{F01}{F00} + \frac{M01}{M00}\right), \frac{1}{2}\left(\frac{F01}{F11} + \frac{M01}{M11}\right)\right] > 1$$

For weak selection, this requires:

```
Min[hf sf - sm (1 - hm), hm sm - sf (1 - hf)] > 0
testpoly[hf_, sf_, hm_, sm_] := (Min[hf sf - sm (1 - hm), hm sm - sf (1 - hf)] > 0)
```

Generally, dominance of the favorable allele in each sex must be high enough to maintain a polymorphic equilibrium with SA selection:

```
testpoly[0.9, 0.1, 0.9, 0.1]
True
testpoly[0.1, 0.1, 0.1, 0.1]
False
```

In the following, we derive the marginal fitnesses of each allele in each sex to be able to derive the necessary selection terms.

Marginal fitness:

```
meanF = x1 * F00 + x2 * F01 + x3 * F11 /. subfit /. pf → pave /. pm → pave // Factor;
meanM = x1 * M00 + x2 * M01 + x3 * M11 /. subfit /. pf → pave /. pm → pave // Factor;
```

The marginal fitnesses in males, $\frac{W_{m,i}}{\overline{W}_m} - 1$:

$$\text{Factor}\left[\text{Normal}\left[\text{Series}\left[\frac{(1-\text{pave})*\text{M00}+\text{pave}*\text{M01}}{\text{meanM}}-1\ /.\ \text{subfit}\ /.\ \text{pave}\ \text{->}\ \frac{(1-\text{hm})\,\text{sm}-\text{hf}\,\text{sf}}{\text{sf}-2\,\text{hf}\,\text{sf}+\text{sm}-2\,\text{hm}\,\text{sm}}\ /.\ \text{sf}\to\text{sf}*\text{small}\ /.\ \text{sm}\to\text{sm}*\text{small},\ \{\text{small},0,1\}\right]\right]\ /.\ \text{small}\to 1\right]$$

$$\frac{(-1+\text{hf}+\text{hm})\,\text{sf}\,\text{sm}\,(\text{hf}\,\text{sf}-\text{sm}+\text{hm}\,\text{sm})}{(-\text{sf}+2\,\text{hf}\,\text{sf}-\text{sm}+2\,\text{hm}\,\text{sm})^2}$$

$$\text{Factor}\left[\text{Normal}\left[\text{Series}\left[\frac{(1-\text{pave})*\text{M01}+\text{pave}*\text{M11}}{\text{meanM}}-1\ /.\ \text{subfit}\ /.\ \text{pave}\ \text{->}\ \frac{(1-\text{hm})\,\text{sm}-\text{hf}\,\text{sf}}{\text{sf}-2\,\text{hf}\,\text{sf}+\text{sm}-2\,\text{hm}\,\text{sm}}\ /.\ \text{sf}\to\text{sf}*\text{small}\ /.\ \text{sm}\to\text{sm}*\text{small},\ \{\text{small},0,1\}\right]\right]\ /.\ \text{small}\to 1\right]$$

$$-\frac{(-1+\text{hf}+\text{hm})\,\text{sf}\,\text{sm}\,(-\text{sf}+\text{hf}\,\text{sf}+\text{hm}\,\text{sm})}{(-\text{sf}+2\,\text{hf}\,\text{sf}-\text{sm}+2\,\text{hm}\,\text{sm})^2}$$

The marginal fitnesses in females, $\frac{W_{f,i}}{\overline{W}_f} - 1$:

$$\text{Factor}\left[\text{Normal}\left[\text{Series}\left[\frac{(1-\text{pave})*\text{F00}+\text{pave}*\text{F01}}{\text{meanF}}-1\ /.\ \text{subfit}\ /.\ \text{pave}\ \text{->}\ \frac{(1-\text{hm})\,\text{sm}-\text{hf}\,\text{sf}}{\text{sf}-2\,\text{hf}\,\text{sf}+\text{sm}-2\,\text{hm}\,\text{sm}}\ /.\ \text{sf}\to\text{sf}*\text{small}\ /.\ \text{sm}\to\text{sm}*\text{small},\ \{\text{small},0,1\}\right]\right]\ /.\ \text{small}\to 1\right]$$

$$-\frac{(-1+\text{hf}+\text{hm})\,\text{sf}\,\text{sm}\,(\text{hf}\,\text{sf}-\text{sm}+\text{hm}\,\text{sm})}{(-\text{sf}+2\,\text{hf}\,\text{sf}-\text{sm}+2\,\text{hm}\,\text{sm})^2}$$

$$\text{Factor}\left[\text{Normal}\left[\text{Series}\left[\frac{(1-\text{pave})*\text{F01}+\text{pave}*\text{F11}}{\text{meanF}}-1\ /.\ \text{subfit}\ /.\ \text{pave}\ \text{->}\ \frac{(1-\text{hm})\,\text{sm}-\text{hf}\,\text{sf}}{\text{sf}-2\,\text{hf}\,\text{sf}+\text{sm}-2\,\text{hm}\,\text{sm}}\ /.\ \text{sf}\to\text{sf}*\text{small}\ /.\ \text{sm}\to\text{sm}*\text{small},\ \{\text{small},0,1\}\right]\right]\ /.\ \text{small}\to 1\right]$$

$$\frac{(-1+\text{hf}+\text{hm})\,\text{sf}\,\text{sm}\,(-\text{sf}+\text{hf}\,\text{sf}+\text{hm}\,\text{sm})}{(-\text{sf}+2\,\text{hf}\,\text{sf}-\text{sm}+2\,\text{hm}\,\text{sm})^2}$$

$$\text{submarg}=\left\{s_0^m\to\frac{(1-\text{hf}-\text{hm})\,\text{sf}\,\text{sm}\,(\text{sm}\,(1-\text{hm})-\text{hf}\,\text{sf})}{(\text{sf}\,(1-2\,\text{hf})+\text{sm}\,(1-2\,\text{hm}))^2},\ s_1^m\to-\frac{(1-\text{hf}-\text{hm})\,\text{sf}\,\text{sm}\,(\text{sf}\,(1-\text{hf})-\text{hm}\,\text{sm})}{(\text{sf}\,(1-2\,\text{hf})+\text{sm}\,(1-2\,\text{hm}))^2},\ s_0^f\to-\frac{(1-\text{hf}-\text{hm})\,\text{sf}\,\text{sm}\,(\text{sm}\,(1-\text{hm})-\text{hf}\,\text{sf})}{(\text{sf}\,(1-2\,\text{hf})+\text{sm}\,(1-2\,\text{hm}))^2},\ s_1^f\to\frac{(1-\text{hf}-\text{hm})\,\text{sf}\,\text{sm}\,(\text{sf}\,(1-\text{hf})-\text{hm}\,\text{sm})}{(\text{sf}\,(1-2\,\text{hf})+\text{sm}\,(1-2\,\text{hm}))^2}\right\};$$

Notice that the marginal allele fitnesses are opposite in males and females. Thus, for this autosomal model, the marginal fitness averaged over the sexes is zero for both alleles, which accounts for the fact that the alleles remain constant in frequency over time.

$s_0^m + s_0^f$ /. submarg

0

$s_1^m + s_1^f$ /. submarg

0

$$\text{subsel}=\left\{s_{Y,0}\to s_0^m,\ s_{Y,1}\to s_1^m,\ s_{X,0}\to\frac{2}{3}s_0^f+\frac{1}{3}s_0^m,\ s_{X,1}\to\frac{2}{3}s_1^f+\frac{1}{3}s_1^m,\ s_{Z,0}\to\frac{1}{3}s_0^f+\frac{2}{3}s_0^m,\ s_{Z,1}\to\frac{1}{3}s_1^f+\frac{2}{3}s_1^m,\ s_{W,0}\to s_0^f,\ s_{W,1}\to s_1^f\right\}\ /.\ \text{submarg};$$

For example, with perfectly symmetrical selection:

```
p1 /. subequil /. subsel /. sm → s /. sf → s /. hm → h /. hf → h // Factor
```

$\frac{1}{2}$

```
{sY,0, sY,1, sX,0, sX,1, sZ,0, sZ,1, sW,0, sW,1} /. subsel /. sm → s /. sf → s /. hm → h /. hf → h // Factor
```

$\left\{\frac{s}{4}, -\frac{s}{4}, -\frac{s}{12}, \frac{s}{12}, \frac{s}{12}, -\frac{s}{12}, -\frac{s}{4}, \frac{s}{4}\right\}$

- **Figures with sexually antagonistic selection**

```
OriginBySex = {μXf → μf, μXm → μm, μY → μm, μZm → μm, μZf → μf, μW → μf} /. μm → α μf;
```

- **All else equal (N_m=N_f and μ_m=μ_f) - no substantial effect**

Equal dominance:

```
selterm = {hm → 0.9, sm → 0.01, hf → 0.9, sf → κ 0.01};
subsel /. selterm
```

$\left\{s_{Y,0} \to -\frac{0.00008\ (0.001 - 0.009\,\kappa)\ \kappa}{(-0.008 - 0.008\,\kappa)^2},\ s_{Y,1} \to \frac{0.00008\ (-0.009 + 0.001\,\kappa)\ \kappa}{(-0.008 - 0.008\,\kappa)^2},\ s_{X,0} \to \frac{0.0000266667\ (0.001 - 0.009\,\kappa)\ \kappa}{(-0.008 - 0.008\,\kappa)^2},\right.$
$s_{X,1} \to -\frac{0.0000266667\ (-0.009 + 0.001\,\kappa)\ \kappa}{(-0.008 - 0.008\,\kappa)^2},\ s_{Z,0} \to -\frac{0.0000266667\ (0.001 - 0.009\,\kappa)\ \kappa}{(-0.008 - 0.008\,\kappa)^2},$
$\left.s_{Z,1} \to \frac{0.0000266667\ (-0.009 + 0.001\,\kappa)\ \kappa}{(-0.008 - 0.008\,\kappa)^2},\ s_{W,0} \to \frac{0.00008\ (0.001 - 0.009\,\kappa)\ \kappa}{(-0.008 - 0.008\,\kappa)^2},\ s_{W,1} \to -\frac{0.00008\ (-0.009 + 0.001\,\kappa)\ \kappa}{(-0.008 - 0.008\,\kappa)^2}\right\}$

For these conditions, we must keep κ between 1/9 and 9 for a protected polymorphism to exist:

```
Reduce[{hf sf - (1 - hm) sm > 0, - (1 - hf) sf + hm sm > 0} /. selterm]
```

Reduce::ratnz : Reduce was unable to solve the system with inexact
coefficients. The answer was obtained by solving a corresponding exact system and numericizing the result. ≫

$0.111111 < \kappa < 9.$

The following confirms that if we add all of the terms, there is very little effect on the relative fixation rates (the small shifts from 1 may reflect the approximations made in calculating the equilibrium):

```
Show[
 Plot[ratioY /. Nf → N / 2 /. Nm → 1 / γ N / 2 /. N → 10000 /. subsel /. subequil /. selterm /. OriginBySex /. α → 1 /. γ → 1,
  {κ, 1 / 9, 9}, PlotStyle → Black, PlotRange → All, AxesOrigin → {0, 0}],
 Plot[ratioW /. Nf → N / 2 /. Nm → 1 / γ N / 2 /. N → 10000 /. subsel /. subequil /. selterm /. OriginBySex /. α → 1 /. γ → 1,
  {κ, 1 / 9, 9}, PlotStyle → {Black, Dashed}, PlotRange → All],
 Plot[ratioZ /. Nf → N / 2 /. Nm → 1 / γ N / 2 /. N → 10000 /. subsel /. subequil /. selterm /. OriginBySex /. α → 1 /. γ → 1,
  {κ, 1 / 9, 9}, PlotStyle → {Black, Dotted}, PlotRange → All],
 PlotRange → {{0, 9}, {0, 1.1}}
]
```

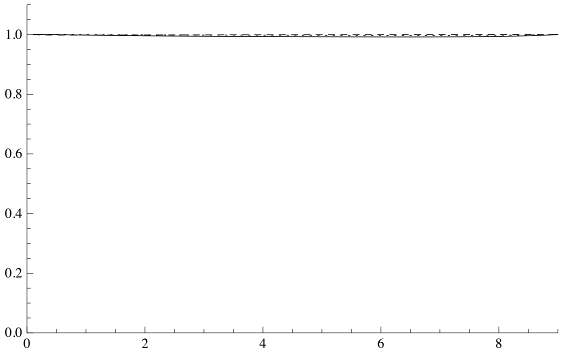

Unequal dominance:

```
selterm = {hm → 0.9, sm → 0.01, hf → 0.5, sf → κ 0.01};
subsel /. selterm
```

$\{s_{Y,0} \to -0.625\ (0.001 - 0.005\,\kappa)\ \kappa,\ s_{Y,1} \to 0.625\ (-0.009 + 0.005\,\kappa)\ \kappa,$
$s_{X,0} \to 0.208333\ (0.001 - 0.005\,\kappa)\ \kappa,\ s_{X,1} \to -0.208333\ (-0.009 + 0.005\,\kappa)\ \kappa,\ s_{Z,0} \to -0.208333\ (0.001 - 0.005\,\kappa)\ \kappa,$
$s_{Z,1} \to 0.208333\ (-0.009 + 0.005\,\kappa)\ \kappa,\ s_{W,0} \to 0.625\ (0.001 - 0.005\,\kappa)\ \kappa,\ s_{W,1} \to -0.625\ (-0.009 + 0.005\,\kappa)\ \kappa\}$

For these conditions, we must keep κ between 1/9 and 9 for a protected polymorphism to exist:

```
Reduce[{hf sf - (1 - hm) sm > 0, - (1 - hf) sf + hm sm > 0} /. selterm]
```

Reduce::ratnz : Reduce was unable to solve the system with inexact
coefficients. The answer was obtained by solving a corresponding exact system and numericizing the result. ≫

$0.2 < \kappa < 1.8$

The following confirms that if we add all of the terms, there is very little effect on the relative fixation rates:

```
Show[
 Plot[ratioY /. Nf → N / 2 /. Nm → 1 / γ N / 2 /. N → 10 000 /. subsel /. subequil /. selterm /. OriginBySex /. α → 1 /. γ → 1,
   {κ, 0.2, 1.8}, PlotStyle → Black, PlotRange → All, AxesOrigin → {0, 0}],
 Plot[ratioW /. Nf → N / 2 /. Nm → 1 / γ N / 2 /. N → 10 000 /. subsel /. subequil /. selterm /. OriginBySex /. α → 1 /. γ → 1,
   {κ, 0.2, 1.8}, PlotStyle → {Black, Dashed}, PlotRange → All],
 Plot[ratioZ /. Nf → N / 2 /. Nm → 1 / γ N / 2 /. N → 10 000 /. subsel /. subequil /. selterm /. OriginBySex /. α → 1 /. γ → 1,
   {κ, 0.2, 1.8}, PlotStyle → {Black, Dotted}, PlotRange → All],
 PlotRange → {{0, 2}, {0, 1.1}}
]
```

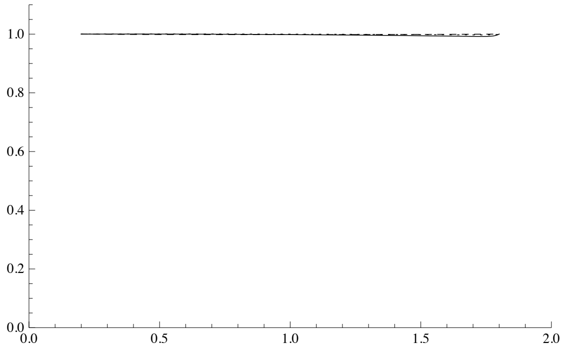

- **Biased sex ratios ($N_m \neq N_f$ and $\mu_m = \mu_f$) - requires $N_m > N_f$ for Y-A fusions to be more common**

Equal dominance:

```
selterm = {hm → 0.9, sm → 0.01, hf → 0.9, sf → κ 0.01};
subsel /. selterm
```

$$\left\{s_{Y,0} \to -\frac{0.00008\,(0.001 - 0.009\,\kappa)\,\kappa}{(-0.008 - 0.008\,\kappa)^2},\ s_{Y,1} \to \frac{0.00008\,(-0.009 + 0.001\,\kappa)\,\kappa}{(-0.008 - 0.008\,\kappa)^2},\ s_{X,0} \to \frac{0.0000266667\,(0.001 - 0.009\,\kappa)\,\kappa}{(-0.008 - 0.008\,\kappa)^2},\right.$$
$$s_{X,1} \to -\frac{0.0000266667\,(-0.009 + 0.001\,\kappa)\,\kappa}{(-0.008 - 0.008\,\kappa)^2},\ s_{Z,0} \to -\frac{0.0000266667\,(0.001 - 0.009\,\kappa)\,\kappa}{(-0.008 - 0.008\,\kappa)^2},$$
$$\left. s_{Z,1} \to \frac{0.0000266667\,(-0.009 + 0.001\,\kappa)\,\kappa}{(-0.008 - 0.008\,\kappa)^2},\ s_{W,0} \to \frac{0.00008\,(0.001 - 0.009\,\kappa)\,\kappa}{(-0.008 - 0.008\,\kappa)^2},\ s_{W,1} \to -\frac{0.00008\,(-0.009 + 0.001\,\kappa)\,\kappa}{(-0.008 - 0.008\,\kappa)^2}\right\}$$

For these conditions, we must keep κ between 1/9 and 9 for a protected polymorphism to exist:

```
Reduce[{hf sf - (1 - hm) sm > 0, - (1 - hf) sf + hm sm > 0} /. selterm]
```

Reduce::ratnz : Reduce was unable to solve the system with inexact
coefficients. The answer was obtained by solving a corresponding exact system and numericizing the result. ≫

$0.111111 < \kappa < 9.$

The following confirms that if $N_m < N_f$ (here N_f/10), then Y-A fusions actually establish less frequently:
[Solid = Y, Dashed = W, Short-dashed = Z, all relative to X]

```
Show[
 Plot[ratioY /. Nf → N / 2 /. Nm → 1 / γ N / 2 /. N → 10 000 /. subsel /. subequil /. selterm /. OriginBySex /. α → 1 /. γ → 10,
   {κ, 1 / 9, 9}, PlotStyle → Black, PlotRange → All, AxesOrigin → {0, 0}],
 Plot[ratioW /. Nf → N / 2 /. Nm → 1 / γ N / 2 /. N → 10 000 /. subsel /. subequil /. selterm /. OriginBySex /. α → 1 /. γ → 10,
   {κ, 1 / 9, 9}, PlotStyle → {Black, Dashed}, PlotRange → All],
 Plot[ratioZ /. Nf → N / 2 /. Nm → 1 / γ N / 2 /. N → 10 000 /. subsel /. subequil /. selterm /. OriginBySex /. α → 1 /. γ → 10,
   {κ, 1 / 9, 9}, PlotStyle → {Black, Dotted}, PlotRange → All],
 PlotRange → {{0, 9}, {0, 10}}, AxesLabel → {"sf/sm", "Relative establishment rate"}
]
```

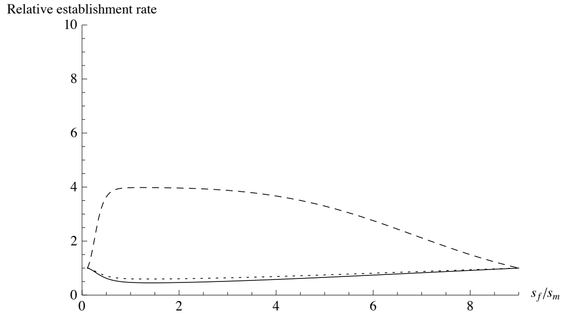

This also demonstrates that the asymmetry in selection (κ) has a relatively minor effect. Below we plot for various sex ratios using $\kappa = 1$ and $\kappa = 5$:

```
tickset =
  {Join[{{Log[0.1], 0.1}}, {{Log[1], 1}}, {{Log[10], 10}}, Flatten[Table[{Log[j 10^i], ""}, {i, -1., 3}, {j, 2, 9}], 1]],
   Join[{{Log[0.1], 0.1}}, Table[{Log[10^i], 10^i}, {i, 0, 3}],
    Flatten[Table[{Log[j 10^i], ""}, {i, -1., 3}, {j, 2, 9}], 1]]};
```

κ=1:
[Solid = Y, Dashed = W, Short-dashed = Z, all relative to X]

```
tryκ = 1;
plot1 = Show[
  LogLogPlot[ratioY /. Nf → N / 2 /. Nm → 1 / γ N / 2 /. N → 10 000 /. subsel /. subequil /. selterm /. κ -> tryκ /. OriginBySex /.
    α → 1, {γ, 1 / 10, 10}, PlotStyle → Black, PlotRange → All, AxesOrigin → {Log[1], Log[0.05]}],
  LogLogPlot[ratioW /. Nf → N / 2 /. Nm → 1 / γ N / 2 /. N → 10 000 /. subsel /. subequil /. selterm /. κ -> tryκ /. OriginBySex /.
    α → 1, {γ, 1 / 10, 10}, PlotStyle → {Black, Dashed}, PlotRange → All], LogLogPlot[
   ratioZ /. Nf → N / 2 /. Nm → 1 / γ N / 2 /. N → 10 000 /. subsel /. subequil /. selterm /. κ -> tryκ /. OriginBySex /. α → 1,
   {γ, 1 / 10, 10}, PlotStyle → {Black, Dotted}, PlotRange → All],
  PlotRange → {{Log[1 / 10], Log[10]}, {Log[0.05], Log[300]}},
  Ticks → tickset, AxesLabel → {"Nf/Nm", "Relative establishment rate"}
 ]
```

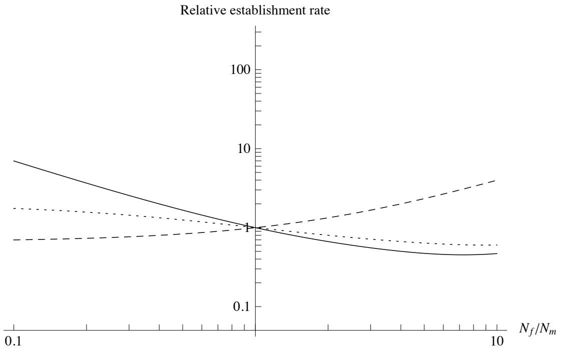

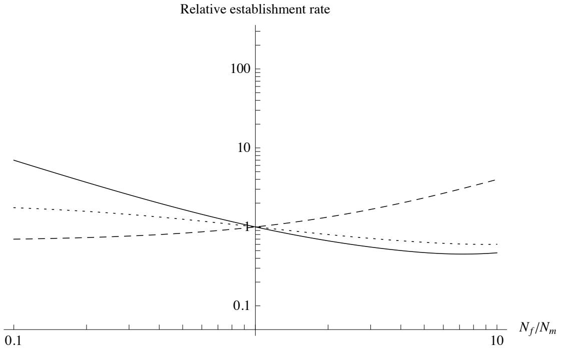

κ=5:

[Solid = Y, Dashed = W, Short-dashed = Z, all relative to X]

```
tryκ = 5;
plot1 = Show[
  LogLogPlot[ratioY /. Nf → N / 2 /. Nm → 1 / γ N / 2 /. N → 10 000 /. subsel /. subequil /. selterm /. κ -> tryκ /. OriginBySex /.
    α → 1, {γ, 1 / 10, 10}, PlotStyle → Black, PlotRange → All, AxesOrigin → {Log[1], Log[0.05]}],
  LogLogPlot[ratioW /. Nf → N / 2 /. Nm → 1 / γ N / 2 /. N → 10 000 /. subsel /. subequil /. selterm /. κ -> tryκ /. OriginBySex /.
    α → 1, {γ, 1 / 10, 10}, PlotStyle → {Black, Dashed}, PlotRange → All], LogLogPlot[
   ratioZ /. Nf → N / 2 /. Nm → 1 / γ N / 2 /. N → 10 000 /. subsel /. subequil /. selterm /. κ -> tryκ /. OriginBySex /. α → 1,
   {γ, 1 / 10, 10}, PlotStyle → {Black, Dotted}, PlotRange → All],
  PlotRange → {{Log[1 / 10], Log[10]}, {Log[0.05], Log[300]}},
  Ticks → tickset, AxesLabel → {"Nf/Nm", "Relative establishment rate"}
 ]
```

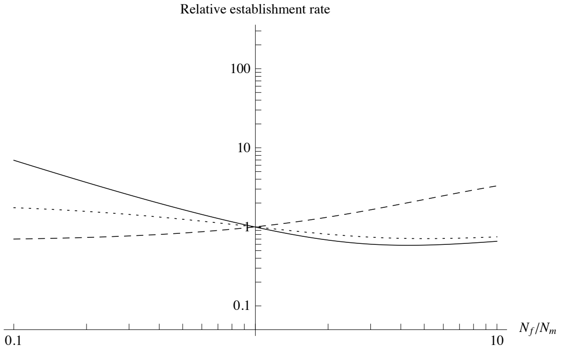

This confirms that we require $N_f < N_m$ in order for sexually-antagonistic selection to account for a Y-A fusion excess.

- **Biased sex ratios ($N_m \neq N_f$ and $\mu_m = \mu_f$) - requires $N_m > N_f$ for Y-A fusions to be more common [Unequal dominance: very similar]**

Equal dominance:

```
selterm = {hm → 0.95, sm → 0.01, hf → 0.85, sf → κ 0.01};
subsel /. selterm
```

$$\left\{s_{Y,0} \to -\frac{0.00008\,(0.0005 - 0.0085\,\kappa)\,\kappa}{(-0.009 - 0.007\,\kappa)^2},\ s_{Y,1} \to \frac{0.00008\,(-0.0095 + 0.0015\,\kappa)\,\kappa}{(-0.009 - 0.007\,\kappa)^2},\ s_{X,0} \to \frac{0.0000266667\,(0.0005 - 0.0085\,\kappa)\,\kappa}{(-0.009 - 0.007\,\kappa)^2},\right.$$
$$s_{X,1} \to -\frac{0.0000266667\,(-0.0095 + 0.0015\,\kappa)\,\kappa}{(-0.009 - 0.007\,\kappa)^2},\ s_{Z,0} \to -\frac{0.0000266667\,(0.0005 - 0.0085\,\kappa)\,\kappa}{(-0.009 - 0.007\,\kappa)^2},$$
$$\left. s_{Z,1} \to \frac{0.0000266667\,(-0.0095 + 0.0015\,\kappa)\,\kappa}{(-0.009 - 0.007\,\kappa)^2},\ s_{W,0} \to \frac{0.00008\,(0.0005 - 0.0085\,\kappa)\,\kappa}{(-0.009 - 0.007\,\kappa)^2},\ s_{W,1} \to -\frac{0.00008\,(-0.0095 + 0.0015\,\kappa)\,\kappa}{(-0.009 - 0.007\,\kappa)^2}\right\}$$

For these conditions, we must keep κ between 1/9 and 9 for a protected polymorphism to exist:

```
Reduce[{hf sf - (1 - hm) sm > 0, - (1 - hf) sf + hm sm > 0} /. selterm]
```

Reduce::ratnz : Reduce was unable to solve the system with inexact coefficients. The answer was obtained by solving a corresponding exact system and numericizing the result. ≫

$0.0588235 < \kappa < 6.33333$

The following confirms that if N_m<N_f (here N_f/10), then Y-A fusions actually establish less frequently:
[Solid = Y, Dashed = W, Short-dashed = Z, all relative to X]

```
Show[
 Plot[ratioY /. Nf → N / 2 /. Nm → 1 / γ N / 2 /. N → 10000 /. subsel /. subequil /. selterm /. OriginBySex /. α → 1 /. γ → 10,
  {κ, 0.059, 6.3}, PlotStyle → Black, PlotRange → All, AxesOrigin → {0, 0}],
 Plot[ratioW /. Nf → N / 2 /. Nm → 1 / γ N / 2 /. N → 10000 /. subsel /. subequil /. selterm /. OriginBySex /. α → 1 /. γ → 10,
  {κ, 0.059, 6.3}, PlotStyle → {Black, Dashed}, PlotRange → All],
 Plot[ratioZ /. Nf → N / 2 /. Nm → 1 / γ N / 2 /. N → 10000 /. subsel /. subequil /. selterm /. OriginBySex /. α → 1 /. γ → 10,
  {κ, 0.059, 6.3}, PlotStyle → {Black, Dotted}, PlotRange → All],
 PlotRange → {{0, 7}, {0, 10}}, AxesLabel → {"sf/sm", "Relative establishment rate"}
]
```

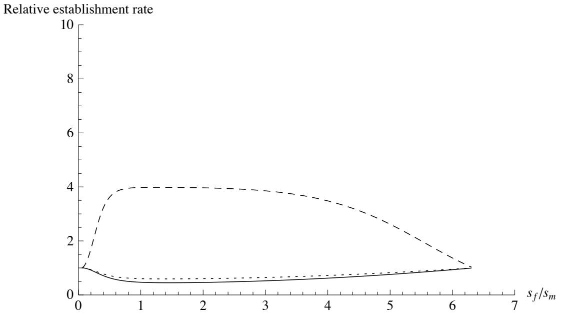

This also demonstrates that the asymmetry in selection (κ) has a relatively minor effect. Below we plot for various sex ratios using $\kappa = 1$ and $\kappa = 5$:

```
tickset =
  {Join[{{Log[0.1], 0.1}}, {{Log[1], 1}}, {{Log[10], 10}}, Flatten[Table[{Log[j 10^i], ""}, {i, -1., 3}, {j, 2, 9}], 1]],
   Join[{{Log[0.1], 0.1}}, Table[{Log[10^i], 10^i}, {i, 0, 3}],
    Flatten[Table[{Log[j 10^i], ""}, {i, -1., 3}, {j, 2, 9}], 1]]};
```

κ=1:
[Solid = Y, Dashed = W, Short-dashed = Z, all relative to X]

```
tryκ = 1;
plot1 = Show[
  LogLogPlot[ratioY /. Nf → N / 2 /. Nm → 1 / γ N / 2 /. N → 10000 /. subsel /. subequil /. selterm /. κ -> tryκ /. OriginBySex /.
    α → 1, {γ, 1 / 10, 10}, PlotStyle → Black, PlotRange → All, AxesOrigin → {Log[1], Log[0.05]}],
  LogLogPlot[ratioW /. Nf → N / 2 /. Nm → 1 / γ N / 2 /. N → 10000 /. subsel /. subequil /. selterm /. κ -> tryκ /. OriginBySex /.
    α → 1, {γ, 1 / 10, 10}, PlotStyle → {Black, Dashed}, PlotRange → All], LogLogPlot[
   ratioZ /. Nf → N / 2 /. Nm → 1 / γ N / 2 /. N → 10000 /. subsel /. subequil /. selterm /. κ -> tryκ /. OriginBySex /. α → 1,
   {γ, 1 / 10, 10}, PlotStyle → {Black, Dotted}, PlotRange → All],
  PlotRange → {{Log[1 / 10], Log[10]}, {Log[0.05], Log[300]}},
  Ticks → tickset, AxesLabel → {"Nf/Nm", "Relative establishment rate"}
 ]
```

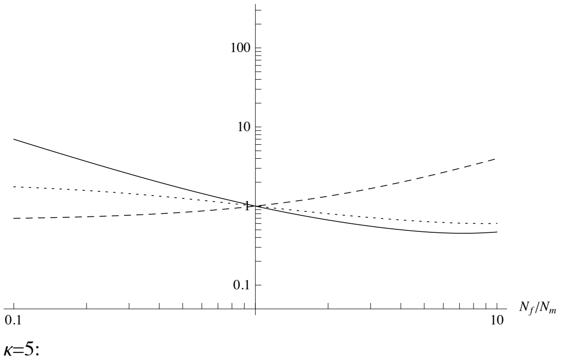

κ=5:
[Solid = Y, Dashed = W, Short-dashed = Z, all relative to X]

```
tryκ = 5;
plot1 = Show[
  LogLogPlot[ratioY /. Nf → N / 2 /. Nm → 1 / γ N / 2 /. N → 10000 /. subsel /. subequil /. selterm /. κ -> tryκ /. OriginBySex /.
     α → 1, {γ, 1 / 10, 10}, PlotStyle → Black, PlotRange → All, AxesOrigin → {Log[1], Log[0.05]}],
  LogLogPlot[ratioW /. Nf → N / 2 /. Nm → 1 / γ N / 2 /. N → 10000 /. subsel /. subequil /. selterm /. κ -> tryκ /. OriginBySex /.
     α → 1, {γ, 1 / 10, 10}, PlotStyle → {Black, Dashed}, PlotRange → All], LogLogPlot[
   ratioZ /. Nf → N / 2 /. Nm → 1 / γ N / 2 /. N → 10000 /. subsel /. subequil /. selterm /. κ -> tryκ /. OriginBySex /. α → 1,
   {γ, 1 / 10, 10}, PlotStyle → {Black, Dotted}, PlotRange → All],
  PlotRange → {{Log[1 / 10], Log[10]}, {Log[0.05], Log[300]}},
  Ticks → tickset, AxesLabel → {"Nf/Nm", "Relative establishment rate"}
 ]
```

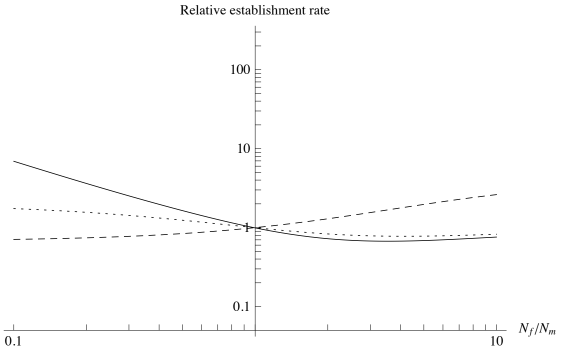

This confirms that we require $N_f < N_m$ in order for sexually-antagonistic selection to account for a Y-A fusion excess.

- **Biased origination rate (N_m=N_f and $\mu_m \neq \mu_f$) - requires $\mu_m > \mu_f$ for Y-A fusions to be more common**

Equal dominance:

```
selterm = {hm → 0.9, sm → 0.01, hf → 0.9, sf → κ 0.01};
subsel /. selterm
```

$$\left\{s_{Y,0} \to -\frac{0.00008\ (0.001 - 0.009\,\kappa)\ \kappa}{(-0.008 - 0.008\,\kappa)^2},\ s_{Y,1} \to \frac{0.00008\ (-0.009 + 0.001\,\kappa)\ \kappa}{(-0.008 - 0.008\,\kappa)^2},\ s_{X,0} \to \frac{0.0000266667\ (0.001 - 0.009\,\kappa)\ \kappa}{(-0.008 - 0.008\,\kappa)^2},\right.$$
$$s_{X,1} \to -\frac{0.0000266667\ (-0.009 + 0.001\,\kappa)\ \kappa}{(-0.008 - 0.008\,\kappa)^2},\ s_{Z,0} \to -\frac{0.0000266667\ (0.001 - 0.009\,\kappa)\ \kappa}{(-0.008 - 0.008\,\kappa)^2},$$
$$\left. s_{Z,1} \to \frac{0.0000266667\ (-0.009 + 0.001\,\kappa)\ \kappa}{(-0.008 - 0.008\,\kappa)^2},\ s_{W,0} \to \frac{0.00008\ (0.001 - 0.009\,\kappa)\ \kappa}{(-0.008 - 0.008\,\kappa)^2},\ s_{W,1} \to -\frac{0.00008\ (-0.009 + 0.001\,\kappa)\ \kappa}{(-0.008 - 0.008\,\kappa)^2}\right\}$$

For these conditions, we must keep κ between 1/9 and 9 for a protected polymorphism to exist:

```
Reduce[{hf sf - (1 - hm) sm > 0, - (1 - hf) sf + hm sm > 0} /. selterm]
```

Reduce::ratnz : Reduce was unable to solve the system with inexact
coefficients. The answer was obtained by solving a corresponding exact system and numericizing the result. ≫

$0.111111 < \kappa < 9.$

The following confirms that if N_m<N_f (here N_f/10), then Y-A fusions actually establish less frequently:
[Solid = Y, Dashed = W, Short-dashed = Z, all relative to X]

```
Show[
 Plot[ratioY /. Nf → N / 2 /. Nm → 1 / γ N / 2 /. N → 10000 /. subsel /. subequil /. selterm /. OriginBySex /. α → 10 /. γ → 1,
  {κ, 1 / 9, 9}, PlotStyle → Black, PlotRange → All, AxesOrigin → {0, 0}],
 Plot[ratioW /. Nf → N / 2 /. Nm → 1 / γ N / 2 /. N → 10000 /. subsel /. subequil /. selterm /. OriginBySex /. α → 10 /. γ → 1,
  {κ, 1 / 9, 9}, PlotStyle → {Black, Dashed}, PlotRange → All],
 Plot[ratioZ /. Nf → N / 2 /. Nm → 1 / γ N / 2 /. N → 10000 /. subsel /. subequil /. selterm /. OriginBySex /. α → 10 /. γ → 1,
  {κ, 1 / 9, 9}, PlotStyle → {Black, Dotted}, PlotRange → All],
 PlotRange → {{0, 9}, {0, 10}}, AxesLabel → {"sf/sm", "Relative establishment rate"}
]
```

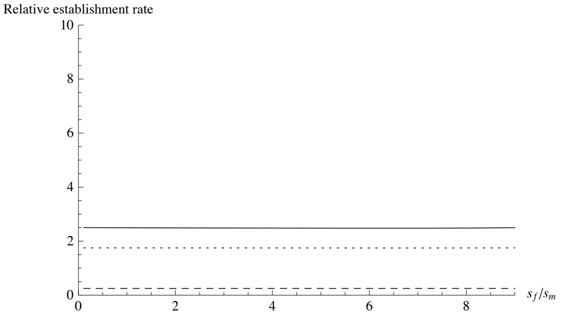

This also demonstrates that the asymmetry in selection (κ) has no effect. Below we plot for various sex ratios using $\kappa = 1$ and $\kappa = 5$:

```
tickset =
  {Join[{{Log[0.1], 0.1}}, {{Log[1], 1}}, {{Log[10], 10}}, Flatten[Table[{Log[j 10^i], ""}, {i, -1., 3}, {j, 2, 9}], 1]],
   Join[{{Log[0.1], 0.1}}, Table[{Log[10^i], 10^i}, {i, 0, 3}],
    Flatten[Table[{Log[j 10^i], ""}, {i, -1., 3}, {j, 2, 9}], 1]]};
```

κ=1:

[Solid = Y, Dashed = W, Short-dashed = Z, all relative to X]

```
tryκ = 1;
plot1 = Show[
  LogLogPlot[ratioY /. Nf → N / 2 /. Nm → 1 / γ N / 2 /. N → 10000 /. subsel /. subequil /. selterm /. κ -> tryκ /. OriginBySex /.
     γ → 1, {α, 1 / 10, 10}, PlotStyle → Black, PlotRange → All, AxesOrigin → {Log[1], Log[0.05]}],
  LogLogPlot[ratioW /. Nf → N / 2 /. Nm → 1 / γ N / 2 /. N → 10000 /. subsel /. subequil /. selterm /. κ -> tryκ /. OriginBySex /.
     γ → 1, {α, 1 / 10, 10}, PlotStyle → {Black, Dashed}, PlotRange → All], LogLogPlot[
   ratioZ /. Nf → N / 2 /. Nm → 1 / γ N / 2 /. N → 10000 /. subsel /. subequil /. selterm /. κ -> tryκ /. OriginBySex /. γ → 1,
   {α, 1 / 10, 10}, PlotStyle → {Black, Dotted}, PlotRange → All],
  PlotRange → {{Log[1 / 10], Log[10]}, {Log[0.05], Log[300]}},
  Ticks → tickset, AxesLabel → {"μm/μf", "Relative establishment rate"}
 ]
```

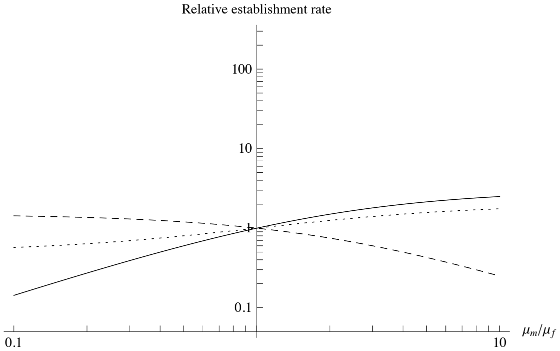

κ=5:

[Solid = Y, Dashed = W, Short-dashed = Z, all relative to X]

```
tryκ = 5;
plot1 = Show[
  LogLogPlot[ratioY /. Nf → N / 2 /. Nm → 1 / γ N / 2 /. N → 10000 /. subsel /. subequil /. selterm /. κ -> tryκ /. OriginBySex /.
     γ → 1, {α, 1 / 10, 10}, PlotStyle → Black, PlotRange → All, AxesOrigin → {Log[1], Log[0.05]}],
  LogLogPlot[ratioW /. Nf → N / 2 /. Nm → 1 / γ N / 2 /. N → 10000 /. subsel /. subequil /. selterm /. κ -> tryκ /. OriginBySex /.
     γ → 1, {α, 1 / 10, 10}, PlotStyle → {Black, Dashed}, PlotRange → All], LogLogPlot[
   ratioZ /. Nf → N / 2 /. Nm → 1 / γ N / 2 /. N → 10000 /. subsel /. subequil /. selterm /. κ -> tryκ /. OriginBySex /. γ → 1,
   {α, 1 / 10, 10}, PlotStyle → {Black, Dotted}, PlotRange → All],
  PlotRange → {{Log[1 / 10], Log[10]}, {Log[0.05], Log[300]}},
  Ticks → tickset, AxesLabel → {"μm/μf", "Relative establishment rate"}
 ]
```

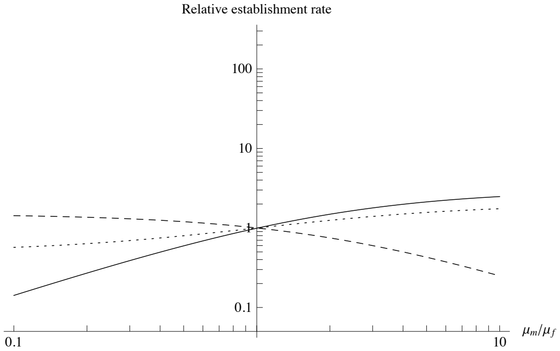

This confirms that we require $\mu_m / \mu_f > 1$ in order for sexually-antagonistic selection to account for a Y-A fusion excess.

- **Biased origination rate (N_m=N_f and $\mu_m \neq \mu_f$) - requires $\mu_m > \mu_f$ for Y-A fusions to be more common [Unequal dominance: very similar]**

Equal dominance:

```
selterm = {hm → 0.95, sm → 0.01, hf → 0.85, sf → κ 0.01};
subsel /. selterm
```

$$\left\{s_{Y,0} \to -\frac{0.00008\,(0.0005 - 0.0085\,\kappa)\,\kappa}{(-0.009 - 0.007\,\kappa)^2},\ s_{Y,1} \to \frac{0.00008\,(-0.0095 + 0.0015\,\kappa)\,\kappa}{(-0.009 - 0.007\,\kappa)^2},\ s_{X,0} \to \frac{0.0000266667\,(0.0005 - 0.0085\,\kappa)\,\kappa}{(-0.009 - 0.007\,\kappa)^2},\right.$$
$$s_{X,1} \to -\frac{0.0000266667\,(-0.0095 + 0.0015\,\kappa)\,\kappa}{(-0.009 - 0.007\,\kappa)^2},\ s_{Z,0} \to -\frac{0.0000266667\,(0.0005 - 0.0085\,\kappa)\,\kappa}{(-0.009 - 0.007\,\kappa)^2},$$
$$\left. s_{Z,1} \to \frac{0.0000266667\,(-0.0095 + 0.0015\,\kappa)\,\kappa}{(-0.009 - 0.007\,\kappa)^2},\ s_{W,0} \to \frac{0.00008\,(0.0005 - 0.0085\,\kappa)\,\kappa}{(-0.009 - 0.007\,\kappa)^2},\ s_{W,1} \to -\frac{0.00008\,(-0.0095 + 0.0015\,\kappa)\,\kappa}{(-0.009 - 0.007\,\kappa)^2}\right\}$$

For these conditions, we must keep κ between 1/9 and 9 for a protected polymorphism to exist:

```
Reduce[{hf sf - (1 - hm) sm > 0, - (1 - hf) sf + hm sm > 0} /. selterm]
```

Reduce::ratnz : Reduce was unable to solve the system with inexact coefficients. The answer was obtained by solving a corresponding exact system and numericizing the result. ≫

$0.0588235 < \kappa < 6.33333$

The following confirms that if $N_m < N_f$ (here $N_f/10$), then Y-A fusions actually establish less frequently:
[Solid = Y, Dashed = W, Short-dashed = Z, all relative to X]

```
Show[
 Plot[ratioY /. Nf → N / 2 /. Nm → 1 / γ N / 2 /. N → 10 000 /. subsel /. subequil /. selterm /. OriginBySex /. α → 10 /. γ → 1,
  {κ, 0.059, 6.3}, PlotStyle → Black, PlotRange → All, AxesOrigin → {0, 0}],
 Plot[ratioW /. Nf → N / 2 /. Nm → 1 / γ N / 2 /. N → 10 000 /. subsel /. subequil /. selterm /. OriginBySex /. α → 10 /. γ → 1,
  {κ, 0.059, 6.3}, PlotStyle → {Black, Dashed}, PlotRange → All],
 Plot[ratioZ /. Nf → N / 2 /. Nm → 1 / γ N / 2 /. N → 10 000 /. subsel /. subequil /. selterm /. OriginBySex /. α → 10 /. γ → 1,
  {κ, 0.059, 6.3}, PlotStyle → {Black, Dotted}, PlotRange → All],
 PlotRange → {{0, 7}, {0, 10}}, AxesLabel → {"sf/sm", "Relative establishment rate"}
]
```

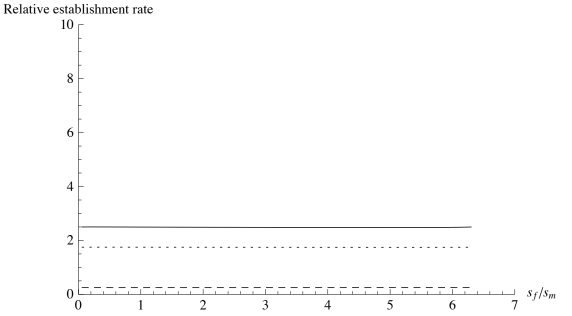

This also demonstrates that the asymmetry in selection (κ) has no effect. Below we plot for various sex ratios using $\kappa = 1$ and $\kappa = 5$:

```
tickset =
  {Join[{{Log[0.1], 0.1}}, {{Log[1], 1}}, {{Log[10], 10}}, Flatten[Table[{Log[j 10^i], ""}, {i, -1., 3}, {j, 2, 9}], 1]],
   Join[{{Log[0.1], 0.1}}, Table[{Log[10^i], 10^i}, {i, 0, 3}],
    Flatten[Table[{Log[j 10^i], ""}, {i, -1., 3}, {j, 2, 9}], 1]]};
```

κ=1:
[Solid = Y, Dashed = W, Short-dashed = Z, all relative to X]

```
tryκ = 1;
plot1 = Show[
  LogLogPlot[ratioY /. Nf → N / 2 /. Nm → 1 / γ N / 2 /. N → 10 000 /. subsel /. subequil /. selterm /. κ -> tryκ /. OriginBySex /.
    γ → 1, {α, 1 / 10, 10}, PlotStyle → Black, PlotRange → All, AxesOrigin → {Log[1], Log[0.05]}],
  LogLogPlot[ratioW /. Nf → N / 2 /. Nm → 1 / γ N / 2 /. N → 10 000 /. subsel /. subequil /. selterm /. κ -> tryκ /. OriginBySex /.
    γ → 1, {α, 1 / 10, 10}, PlotStyle → {Black, Dashed}, PlotRange → All], LogLogPlot[
   ratioZ /. Nf → N / 2 /. Nm → 1 / γ N / 2 /. N → 10 000 /. subsel /. subequil /. selterm /. κ -> tryκ /. OriginBySex /. γ → 1,
   {α, 1 / 10, 10}, PlotStyle → {Black, Dotted}, PlotRange → All],
  PlotRange → {{Log[1 / 10], Log[10]}, {Log[0.05], Log[300]}},
  Ticks → tickset, AxesLabel → {"μm/μf", "Relative establishment rate"}
 ]
```

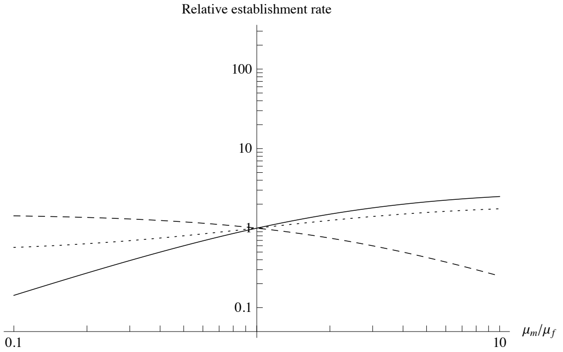

κ=5:
[Solid = Y, Dashed = W, Short-dashed = Z, all relative to X]

```
tryϰ = 5;
plot1 = Show[
  LogLogPlot[ratioY /. Nf → N / 2 /. Nm → 1 / γ N / 2 /. N → 10 000 /. subsel /. subequil /. selterm /. ϰ -> tryϰ /. OriginBySex /.
     γ → 1, {α, 1 / 10, 10}, PlotStyle → Black, PlotRange → All, AxesOrigin → {Log[1], Log[0.05]}],
  LogLogPlot[ratioW /. Nf → N / 2 /. Nm → 1 / γ N / 2 /. N → 10 000 /. subsel /. subequil /. selterm /. ϰ -> tryϰ /. OriginBySex /.
     γ → 1, {α, 1 / 10, 10}, PlotStyle → {Black, Dashed}, PlotRange → All], LogLogPlot[
   ratioZ /. Nf → N / 2 /. Nm → 1 / γ N / 2 /. N → 10 000 /. subsel /. subequil /. selterm /. ϰ -> tryϰ /. OriginBySex /. γ → 1,
   {α, 1 / 10, 10}, PlotStyle → {Black, Dotted}, PlotRange → All],
  PlotRange → {{Log[1 / 10], Log[10]}, {Log[0.05], Log[300]}},
  Ticks → tickset, AxesLabel → {"μm/μf", "Relative establishment rate"}
 ]
```

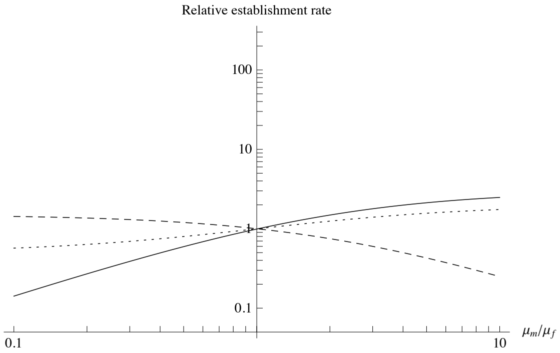

This confirms that we require $\mu_m / \mu_f > 1$ in order for sexually-antagonistic selection to account for a Y-A fusion excess.

Allowing both biased sex-ratios and sex-specific origination rates

```
tickset =
  {Join[Table[{i, i}, {i, -0.001, 0.001, 0.0005}], Flatten[Table[{Log[j 10^i], ""}, {i, -1., 3}, {j, 2, 9}], 1]], Join[
    {{Log[0.1], 0.1}}, Table[{Log[10^i], 10^i}, {i, 0, 3}], Flatten[Table[{Log[j 10^i], ""}, {i, -1., 3}, {j, 2, 9}], 1]]};
selterm = {hm → 0.9, sm → 0.01, hf → 0.9, sf → ϰ 0.01};
Manipulate[Show[
  LogPlot[ratioY /. Nf → N / 2 /. Nm → (1/γ) N / 2 /. subsel /. subequil /. selterm /. N → 10 000 /. {μX^f → μ^f, μX^m → μ^m, μY → μ^m,
     μZ^m → μ^m, μZ^f → μ^f, μW → μ^f} /. μ^m → α μ^f, {ϰ, 1 / 9, 9}, AxesOrigin → {0, Log[0.05]}, PlotStyle → Black],
  LogPlot[ratioW /. Nf → N / 2 /. Nm → (1/γ) N / 2 /. subsel /. subequil /. selterm /. N → 10 000 /.
     {μX^f → μ^f, μX^m → μ^m, μY → μ^m, μZ^m → μ^m, μZ^f → μ^f, μW → μ^f} /. μ^m → α μ^f, {ϰ, 1 / 9, 9}, PlotStyle → {Black, Dashed}],
  LogPlot[ratioZ /. Nf → N / 2 /. Nm → (1/γ) N / 2 /. subsel /. subequil /. selterm /. N → 10 000 /.
     {μX^f → μ^f, μX^m → μ^m, μY → μ^m, μZ^m → μ^m, μZ^f → μ^f, μW → μ^f} /. μ^m → α μ^f, {ϰ, 1 / 9, 9}, PlotStyle → {Black, Dotted}],
  PlotRange → {{1 / 9, 9}, {Log[0.05], Log[1000]}},
  Ticks → tickset, AxesLabel → {"sf/sm", "Relative establishment rate"}], {{α, 1}, 0.1, 10}, {{γ, 1}, 0.1, 10}]
```

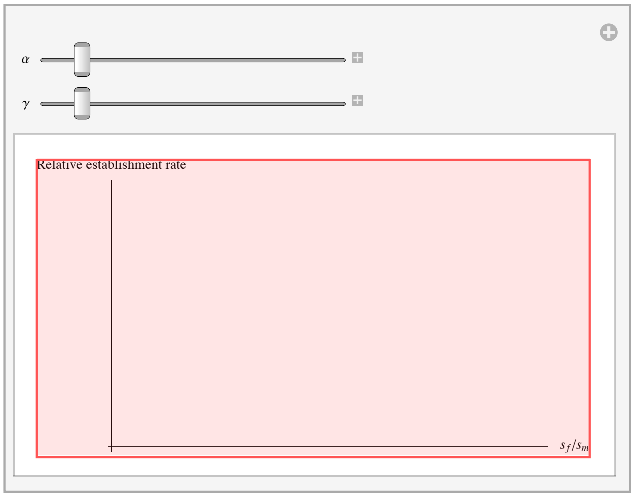

ReplaceAll::reps : {subsel} is neither a list of replacement rules nor a valid dispatch table, and so cannot be used for replacing. ≫

ReplaceAll::reps : {subequil} is neither a list of replacement rules nor a valid dispatch table, and so cannot be used for replacing. ≫

ReplaceAll::reps : {selterm} is neither a list of replacement rules nor a valid dispatch table, and so cannot be used for replacing. ≫

General::stop : Further output of ReplaceAll::reps will be suppressed during this calculation. ≫

Simulations

The simulations below were used to estimate the relative rates of establishment for Y-A and X-A fusions, as reported by the dots in

- **Code [ENTER]**
- **Recursions**

Generation: census -> gamete union -> selection -> reproduction.

```
Clear[simfullYA]
simfullYA[Nf_, Nm_, N_, sf_, sm_, maxtime_] := Block[{Njuv = N / 2},
  deaddad = If[RandomReal[] < Nm / Njuv, 0, 1];
  If[(deaddad == 0),
   lost = 0; fixed = 0; startfreq = 1 / Nm;
  ];
  If[(deaddad == 1),
   lost = 1; fixed = 0;
  ];

  freq = startfreq;
  t = 0;
  While[(t ≤ maxtime) && (lost == 0) && (fixed == 0),
   t++;
   freq = RandomInteger[BinomialDistribution[Nm, (1 + sm) freq / (1 + sm freq)]] / Nm;
   If[(freq) < 10^-12, lost = 1];
   If[(freq) > 1 - 10^-12, fixed = 1];
  ];
  {t, lost, fixed}
 ]
```

For females, we introduce a single fused chromosome. The rate of such fusions is expected to be $2\,\frac{N}{2}\,\mu_X^f$ in females and $\frac{N}{2}\,\mu_X^m$ in males. Dividing by the sum rate ($N\,\mu_X^f + \frac{N}{2}\,\mu_X^m$), gives the chance of a particular fusion appearing in a female: $\frac{2\,\frac{N}{2}\,\mu_X^f}{N\,\mu_X^f + \frac{N}{2}\,\mu_X^m} = \frac{2}{2+\alpha}$.

```
Clear[simfullXA]
simfullXA[Nf_, Nm_, N_, sf_, sm_, maxtime_, α_] := Block[{Njuv = N / 2}, (*# of juveniles of each sex*)

  inmom = RandomInteger[BernoulliDistribution[2/(2 + α)]];
  If[inmom == 0, (*First appears in a male*)
   deaddad = If[RandomReal[] < Nm / Njuv, 0, 1]; (*If 1, he fails to become a reproductive male*)
   If[(deaddad == 0),
    lost = 0; fixed = 0;
    startfreq = {0, 1 / Nm} (*Frequency of X-fusions among Xs in females and males*)
   ];
   If[(deaddad == 1),
    lost = 1; fixed = 0;
   ];
  ];

  If[inmom == 1, (*First appears in a female*)
   deadmom = If[RandomReal[] < Nf / Njuv, 0, 1]; (*If 1, she fails to become a reproductive female*)
   If[(deadmom == 0),
    lost = 0; fixed = 0;
    startfreq = {1 / (2 Nf), 0} (*Frequency of X-fusions among Xs in females and males*)
   ];
   If[(deadmom == 1),
    lost = 1; fixed = 0;
   ];
  ];

  freq = startfreq;
  t = 0;
  While[(t ≤ maxtime) && (lost == 0) && (fixed == 0),
   t++;
   newdad = RandomInteger[BinomialDistribution[Nm, ((1 + sm) (freq[[1]]))/(1 + sm (freq[[1]]))]] / Nm;
   (*Freq of X-fusions among reproducing males*)
   starthet = (freq[[1]]) * (1 - freq[[2]]) + (1 - freq[[1]]) * freq[[2]];
   (*Freq of X-fusions born in heterozygous females*)
   starthom = (freq[[1]]) * (freq[[2]]); (*Freq of X-fusions born in homozygous females*)
   homhet = {((1 + sf) starthet)/(1 + sf starthet + 2 sf starthom), ((1 + 2 sf) starthom)/(1 + sf starthet + 2 sf starthom)};
   (*Freq of het or homo X-fusions in females, after selection, assuming additive selection*)
   pvec = Chop[{1 - homhet[[1]] - homhet[[2]], homhet[[1]], homhet[[2]]}];
   newmoms = RandomInteger[MultinomialDistribution[Nf, pvec]];
   (*Number of reproducing wildtype, het, hom X-fusion females*)
   freq = {newmoms[[2]] / (2 Nf) + newmoms[[3]] / (Nf), newdad};
   If[(freq.{2 / 3, 1 / 3}) < 10^-12, lost = 1];
   If[(freq.{2 / 3, 1 / 3}) > 1 - 10^-12, fixed = 1];
  ];
  {t, lost, fixed}
 ]
```

- **Y-autosome fusion ($\gamma = 5$)**

Fixation probability for a Y-autosome fusion for the case shown in Figure 4

```
SeedRandom[93 112]

maxtrial = 1 000 000;

For[trial = 1, trial ≤ maxtrial, trial++,
 temp[trial] = simfullYA[5000, 1000, 10 000, -0.0003, -0.0003, 10^6]
]
Table[temp[i][[3]], {i, 1, maxtrial}] // Total
% / maxtrial // N
```

```
136

0.000136
```

```
For[trial = 1, trial ≤ maxtrial, trial++,
 temp[trial] = simfullYA[5000, 1000, 10 000, 0, 0, 10^6]
]
Table[temp[i][[3]], {i, 1, maxtrial}] // Total
% / maxtrial // N
```

```
184

0.000184
```

```
For[trial = 1, trial ≤ maxtrial, trial++,
 temp[trial] = simfullYA[5000, 1000, 10 000, 0.0003, 0.0003, 10^6]
]
Table[temp[i][[3]], {i, 1, maxtrial}] // Total
% / maxtrial // N
```

283

0.000283

- **Y-autosome fusion ($\gamma = 10$)**

Fixation probability for a Y-autosome fusion for the case shown in Figure 4

```
SeedRandom[98 347]
```

```
maxtrial = 1 000 000;
```

```
For[trial = 1, trial ≤ maxtrial, trial++,
 temp[trial] = simfullYA[5000, 500, 10 000, -0.0003, -0.0003, 10^6]
]
Table[temp[i][[3]], {i, 1, maxtrial}] // Total
% / maxtrial // N
```

179

0.000179

```
For[trial = 1, trial ≤ maxtrial, trial++,
 temp[trial] = simfullYA[5000, 500, 10 000, 0, 0, 10^6]
]
Table[temp[i][[3]], {i, 1, maxtrial}] // Total
% / maxtrial // N
```

185

0.000185

```
For[trial = 1, trial ≤ maxtrial, trial++,
 temp[trial] = simfullYA[5000, 500, 10 000, 0.0003, 0.0003, 10^6]
]
Table[temp[i][[3]], {i, 1, maxtrial}] // Total
% / maxtrial // N
```

230

0.00023

- **X-autosome fusion ($\gamma = 5$)**

Fixation probability for a X-autosome fusion for the case shown in Figure 4

```
SeedRandom[912 537]
```

```
maxtrial = 1 000 000;
```

```
For[trial = 1, trial ≤ maxtrial, trial++,
 temp[trial] = simfullXA[5000, 1000, 10 000, -0.0003, -0.0003, 10^6, 1]
]
Table[temp[i][[3]], {i, 1, maxtrial}] // Total
% / maxtrial // N
```

7

$7. \times 10^{-6}$

```
SeedRandom[83 471]
```

```
maxtrial = 1 000 000;
```

```
For[trial = 1, trial ≤ maxtrial, trial++,
 temp[trial] = simfullXA[5000, 1000, 10 000, 0, 0, 10^6, 1]
]
Table[temp[i][[3]], {i, 1, maxtrial}] // Total
% / maxtrial // N
```

56

0.000056

```
For[trial = 1, trial ≤ maxtrial, trial++,
 temp[trial] = simfullXA[5000, 1000, 10 000, 0.0003, 0.0003, 10^6, 1]
]
Table[temp[i][[3]], {i, 1, maxtrial}] // Total
% / maxtrial // N
```

260

0.00026

- **X-autosome fusion ($\gamma = 10$)**

Fixation probability for a X-autosome fusion for the case shown in Figure 4

```
SeedRandom[127 934]

maxtrial = 1 000 000;

For[trial = 1, trial ≤ maxtrial, trial++,
 temp[trial] = simfullXA[5000, 500, 10 000, -0.0003, -0.0003, 10^6, 1]
]
Table[temp[i][[3]], {i, 1, maxtrial}] // Total
% / maxtrial // N
```

15

0.000015

```
For[trial = 1, trial ≤ maxtrial, trial++,
 temp[trial] = simfullXA[5000, 500, 10 000, 0, 0, 10^6, 1]
]
Table[temp[i][[3]], {i, 1, maxtrial}] // Total
% / maxtrial // N
```

83

0.000083

```
For[trial = 1, trial ≤ maxtrial, trial++,
 temp[trial] = simfullXA[5000, 500, 10 000, 0.0003, 0.0003, 10^6, 1]
]
Table[temp[i][[3]], {i, 1, maxtrial}] // Total
% / maxtrial // N
```

163

0.000163

- **Y-autosome fusion ($\gamma = 1$ for use with varying α)**

Fixation probability for a Y-autosome fusion for the case shown in Figure 4

```
SeedRandom[636 411]

maxtrial = 1 000 000;

For[trial = 1, trial ≤ maxtrial, trial++,
 temp[trial] = simfullYA[5000, 5000, 10 000, -0.0003, -0.0003, 10^6]
]
Table[temp[i][[3]], {i, 1, maxtrial}] // Total
% / maxtrial // N
```

33

0.000033

```
For[trial = 1, trial ≤ maxtrial, trial++,
 temp[trial] = simfullYA[5000, 5000, 10 000, 0, 0, 10^6]
]
Table[temp[i][[3]], {i, 1, maxtrial}] // Total
% / maxtrial // N
```

211

0.000211

```
For[trial = 1, trial ≤ maxtrial, trial++,
 temp[trial] = simfullYA[5000, 5000, 10 000, 0.0003, 0.0003, 10^6]
]
Table[temp[i][[3]], {i, 1, maxtrial}] // Total
% / maxtrial // N
```

622

0.000622

- **X-autosome fusion ($\alpha = 5$)**

Fixation probability for a X-autosome fusion for the case shown in Figure 4

```
SeedRandom[934 812]

maxtrial = 1 000 000;

For[trial = 1, trial ≤ maxtrial, trial++,
 temp[trial] = simfullXA[5000, 5000, 10 000, -0.0003, -0.0003, 10^6, 5]
]
Table[temp[i][[3]], {i, 1, maxtrial}] // Total
% / maxtrial // N

0

0.

For[trial = 1, trial ≤ maxtrial, trial++,
 temp[trial] = simfullXA[5000, 5000, 10 000, 0, 0, 10^6, 5]
]
Table[temp[i][[3]], {i, 1, maxtrial}] // Total
% / maxtrial // N

62

0.000062

For[trial = 1, trial ≤ maxtrial, trial++,
 temp[trial] = simfullXA[5000, 5000, 10 000, 0.0003, 0.0003, 10^6, 5]
]
Table[temp[i][[3]], {i, 1, maxtrial}] // Total
% / maxtrial // N

588

0.000588
```

- **X-autosome fusion ($\alpha = 10$)**

Fixation probability for a X-autosome fusion for the case shown in Figure 4

```
SeedRandom[300 312]

maxtrial = 1 000 000;

For[trial = 1, trial ≤ maxtrial, trial++,
 temp[trial] = simfullXA[5000, 5000, 10 000, -0.0003, -0.0003, 10^6, 10]
]
Table[temp[i][[3]], {i, 1, maxtrial}] // Total
% / maxtrial // N

0

0.

For[trial = 1, trial ≤ maxtrial, trial++,
 temp[trial] = simfullXA[5000, 5000, 10 000, 0, 0, 10^6, 10]
]
Table[temp[i][[3]], {i, 1, maxtrial}] // Total
% / maxtrial // N

71

0.000071

For[trial = 1, trial ≤ maxtrial, trial++,
 temp[trial] = simfullXA[5000, 5000, 10 000, 0.0003, 0.0003, 10^6, 10]
]
Table[temp[i][[3]], {i, 1, maxtrial}] // Total
% / maxtrial // N

579

0.000579
```
