# Supplementary figures and images for: Y Fuse? Sex Chromosome Fusions in Fishes and Reptiles
Source: PLoS Genet. 2015 May 20;11(5):e1005237. doi: 10.1371/journal.pgen.1005237 (PMC4439076; doi:10.1371/journal.pgen.1005237)

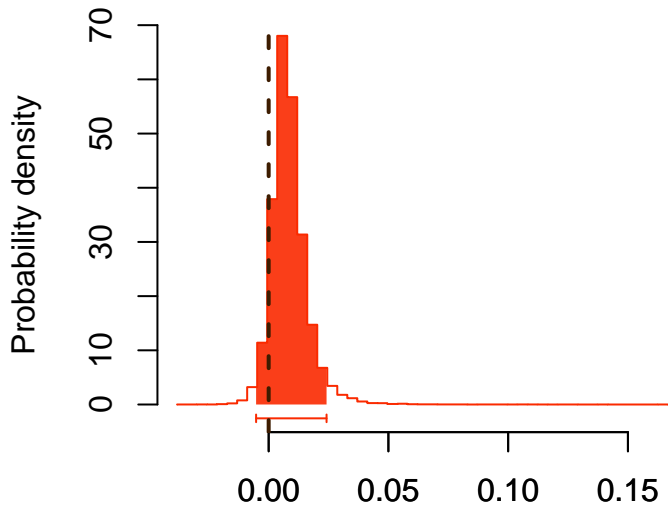

Difference between XY and ZW fusion rates

Supplement: S1 Fig — Posterior estimate of the rate difference between XY and ZW fusions (q XY.XYF—q ZW.ZWF) in squamate reptiles when we allow the fission rates q XYF.XY and q ZWF.ZW to differ is shown. (PDF) [file pgen.1005237.s004.pdf]

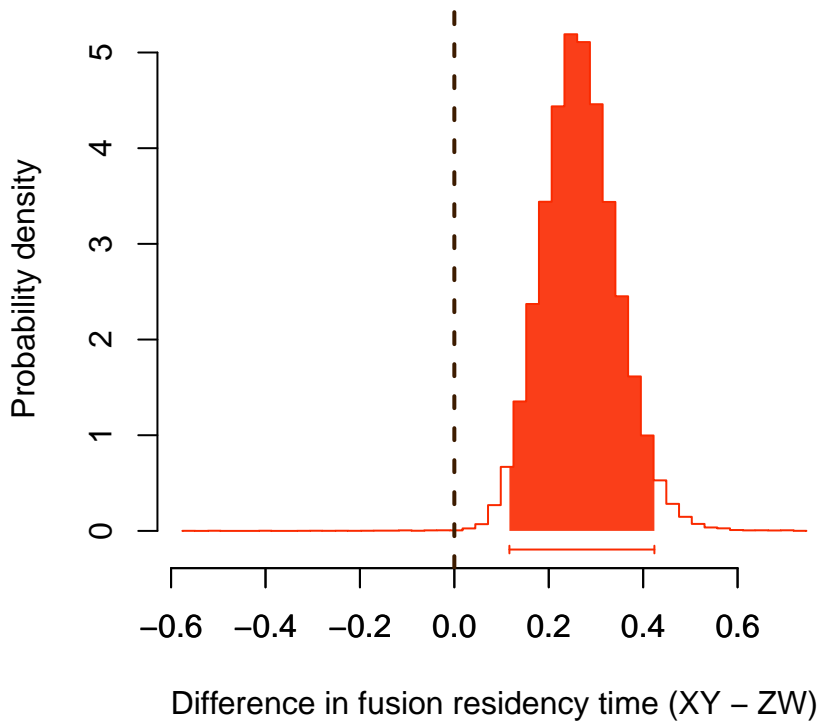

Supplement: S2 Fig — Posterior estimate of the rate difference between XY and ZW fusions (q XY.XYF—q ZW.ZWF) in squamate reptiles when we allow the fission rates q XYF.XY and q ZWF.ZW to differ is shown. (PDF) [file pgen.1005237.s005.pdf]

Probability density

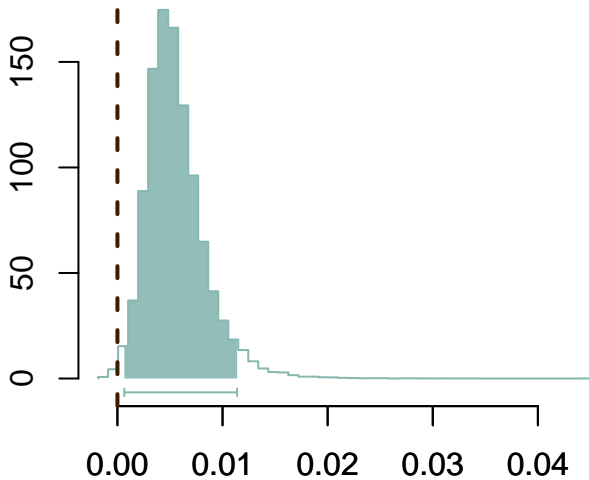

Difference between YA and XA/ZA fusion rates

Supplement: S3 Fig — Posterior estimate of the rate difference between YA and XA/ZA fusions in fish is shown. When the estimate is greater than zero, this means that the YA fusion rates are higher than those of the other chromosomes. (PDF) [file pgen.1005237.s006.pdf]

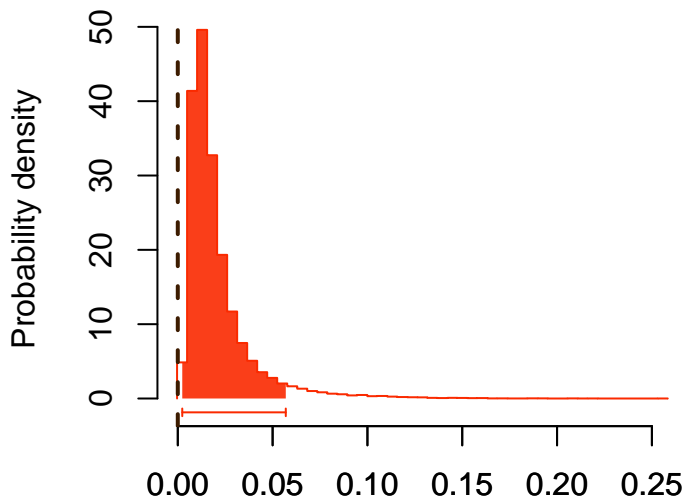

Difference between YA and WA/ZA fusion rates

Supplement: S4 Fig — Posterior estimate of the rate difference between YA and WA/ZA fusions in squamate reptiles is shown. When the estimate is greater than zero, this means that the YA fusion rates are higher than those of the other chromosomes. (PDF) [file pgen.1005237.s007.pdf]
